# Molecular Dynamics Simulations of the Cardiac Ryanodine Receptor Type 2 (RyR2) Gating Mechanism

**DOI:** 10.1021/acs.jpcb.2c03031

**Published:** 2022-11-16

**Authors:** D’Artagnan Greene, Michael Barton, Tyler Luchko, Yohannes Shiferaw

**Affiliations:** Department of Physics and Astronomy, California State University, Northridge, California 91330, United States

## Abstract

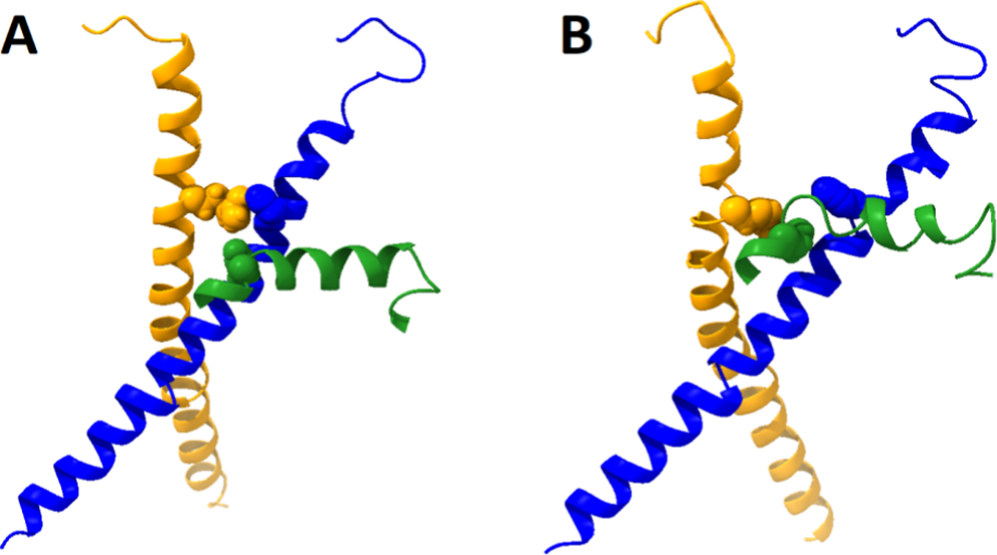

Mutations in the cardiac ryanodine receptor type 2 (RyR2) have been linked to fatal cardiac arrhythmias such as catecholaminergic polymorphic ventricular tachycardia (CPVT). While many CPVT mutations are associated with an increase in Ca^2+^ leak from the sarcoplasmic reticulum, the mechanistic details of RyR2 channel gating are not well understood, and this poses a barrier in the development of new pharmacological treatments. To address this, we explore the gating mechanism of the RyR2 using molecular dynamics (MD) simulations. We test the effect of changing the conformation of certain structural elements by constructing chimera RyR2 structures that are derived from the currently available closed and open cryo-electron microscopy (cryo-EM) structures, and we then use MD simulations to relax the system. Our key finding is that the position of the S4–S5 linker (S4S5L) on a single subunit can determine whether the channel as a whole is open or closed. Our analysis reveals that the position of the S4S5L is regulated by interactions with the U-motif on the same subunit and with the S6 helix on an adjacent subunit. We find that, in general, channel gating is crucially dependent on high percent occupancy interactions between adjacent subunits. We compare our interaction analysis to 49 CPVT1 mutations in the literature and find that 73% appear near a high percent occupancy interaction between adjacent subunits. This suggests that disruption of cooperative, high percent occupancy interactions between adjacent subunits is a primary cause of channel leak and CPVT in mutant RyR2 channels.

## Introduction

The cardiac ryanodine receptor type 2 (RyR2) is a gated channel protein that plays a central role in cardiac excitation–contraction coupling.^1,2^ During electrical excitation, a transient, local increase in cytosolic Ca^2+^ ions triggers RyR2 channels to open, releasing additional Ca^2+^ ions from the sarcoplasmic reticulum (SR). The released Ca^2+^ ions diffuse into the cell and trigger cell contraction, which is responsible for the mechanical pumping of blood in the heart.^3,4^ After opening, the RyR2 channel must close for the SR to refill before releasing Ca^2+^ ions in the next heartbeat.^5^

Defects in RyR2 channel gating have been linked to fatal cardiac arrhythmias such as catecholaminergic polymorphic ventricular tachycardia (CPVT).^6−13^ Single-cell studies reveal that many CPVT mutations are associated with a persistent leak of Ca^2+^ ions from the SR, and this has been attributed to an increase in the open probability of the RyR2.^14−16^ Further studies have shown that an increased RyR2 open probability promotes Ca^2+^ waves within cells, and these waves induce electrical excitations that can cause cardiac arrhythmias.^17,18^ However, detailed mechanisms by which the mutations lead to a higher open probability of the RyR2 are not known. The key role of the RyR2 in serious diseases such as CPVT has identified the RyR2 as a potential drug target, but the lack of a clear mechanism that describes channel gating has remained a roadblock in the development of new pharmacological agents.^19,20^

Structural biologists have been trying to understand the mechanism of RyR channel gating for over 20 years.^1,21−35^ Since 2015, several high resolution cryo-electron microscopy (cryo-EM) structures of ryanodine receptors have been made available in both the closed and open states of the channel,^29,30,32,36^ and key structural elements that may be involved in ryanodine receptor channel gating have been identified.^31−34,37^ Despite these advances, the exact mechanism of RyR2 channel gating remains unclear. One way to explore the mechanism of channel gating is to use computational analysis techniques on the known cryo-EM structures. A few recent molecular dynamics (MD) studies have focused on channel gating in the transmembrane region of the ryanodine receptor type 1 (RyR1) channel.^38−40^ However, due to the long-timescale dynamics of the open-to-closed transition, the microsecond timeframe of an all-atom MD simulation is typically not long enough to observe a gating event. To overcome this limitation, several methods have been introduced to either apply a force directly to the system, or, alternatively, recent methods were developed that change certain structural elements within the system at the onset of the MD simulation to induce the transition. After this change is introduced, unbiased MD is carried out for ∼1 μs to allow the system to fully adjust to the change.^41−48^ Our approach took inspiration from a study by Nury et al.^47^ on the channel gating of a nicotinic receptor homologue.^47^ In this study, an instantaneous change in pH was introduced to the open state of the channel protein to induce the channel to close. It was shown that there was a quick adjustment to the pore radius that effectively closed the channel within 50 ns. This was followed by a slow twist for ∼450 ns and a slow relaxation up to the 1 μs simulation run time. The results were in agreement with prior studies that had employed similar methods, and the final structure was shown to be consistent with known data on the crystal structure of the closed state of the nicotinic receptor homologue.^47^

Here, we examine the effects of conformational changes, point mutations, and the role of cooperativity on the gating mechanism of the RyR2 channel. We do this using chimera RyR2 structures that can be induced to transition between the open and closed states of the RyR2 channel using MD simulations. These chimera structures contain features of individual closed- and open-state RyR2 subunits that are instantaneously blended into a single, four-subunit channel structure at the onset of the MD simulation. MD simulations based on our chimera RyR2 models predict that changes in the conformation and positioning of the S4–S5 linker (S4S5L) within a single RyR2 subunit can lead to cooperative channel closing of the entire four-subunit channel by adjusting the radius of the central, hydrophobic gate. To model changes in the S4S5L on channel gating, we also introduced a known CPVT point mutation that is located within the S4S5L (H4762P)^6,12^ into a closed subunit within our chimera model, and we show how this single point mutation can lead to defective channel closing that is associated with channel leak and CPVT. Additionally, an analysis of our MD trajectories identified the key high percent occupancy interactions between the S4S5L, U-motif, and the S6 helix. This analysis revealed that there is a shift in key high percent occupancy interactions between adjacent subunits that are involved in cooperatively closing the RyR2 channel. We compared our high percent occupancy interaction analysis to 49 CPVT1 mutations in the literature and found that 73% of these mutations are within the range of a high percent occupancy interaction site that appears at a subunit–subunit interface. These data suggest that disrupting high percent occupancy interactions between adjacent subunits is a general mechanism that can lead to channel leak and CPVT in mutant RyR2 channels.

## Methods

### Preparation of RyR2 Systems for MD

**Figure 1:**
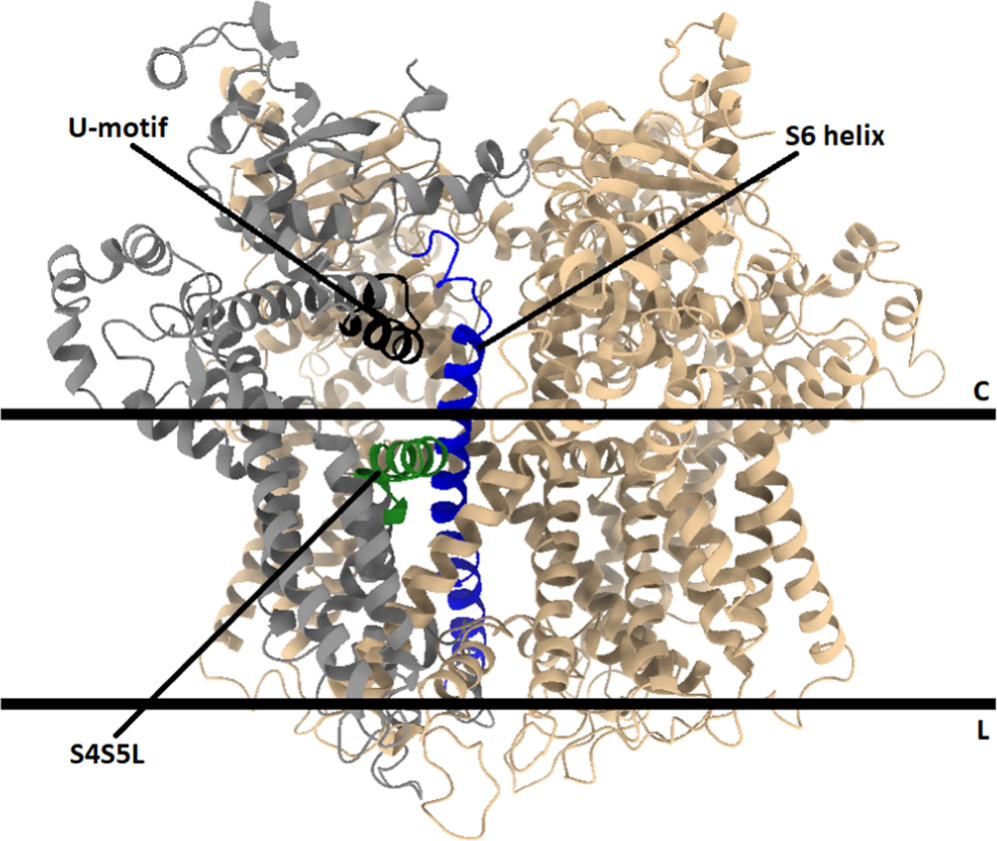
Computational model of the RyR2 system. A computational model of the closed (4C) RyR2 system containing a portion of the central domain (residues 4099–4206) and the transmembrane channel domain (residues 4485–4963) is shown. Key structural elements such as the U-motif (residues 4167–4184, black), the S4S5L (residues 4746–4766, green), and the S6 helix (residues 4839–4889, blue) are highlighted in the first subunit (gray). The extent of the membrane region is roughly indicated by two horizontal black lines, and the cytosolic and luminal regions are indicated by C and L, respectively. This image of the channel is an average structure taken over the last 100 ns of a 1 μs MD simulation. Water molecules, DPPC lipids, and counterions have been omitted from this image for clarity. The image of the RyR2 channel was produced using UCSF Chimera X 1.4.

Two RyR2 structures were downloaded from the protein data bank: RyR2 in the closed state (PDB ID: 6JI8) and RyR2 in the open state (PDB ID: 6JIY).^36,49^ These represent cryo-EM structures of the porcine RyR2 system with resolutions of 3.6 and 3.9 Å for the closed and open states, respectively. The PDB files were edited to contain residues 4099–4206, which was a portion of the cytosolic central domain that included the U-motif, and residues 4485–4963, which contained the transmembrane channel domain and a portion of the C-terminal domain, in each of the four subunits for both RyR2 systems (Figure 1). These represented the largest protein structures that we could include in our model; as also pointed out in a recent study on RyR1 by Heinz et al.,^50^ the full regulatory region of the RyR2 channel (residues 1–4098) spreads out laterally, which drastically increases the computational cost of the explicit solvation of this system. Aside from the atomic coordinates for each of the four subunits containing the sequence of amino acid residues given above, all other information in the original PDB file was deleted. We note that within the transmembrane domain (residues 4485–4963), two disordered segments (residues 4524–4556 and 4672–4694) were missing in each subunit in the original PDB files, and as a result, are also missing in our model systems. An additional residue, 4523, was missing in each subunit in the PDB file for the open state (PDB ID: 6JIY).

The residues at the terminal ends of each subunit were assigned to be standard charged amino acids. The residue ends of the internal missing sequences were connected together to form peptide bonds that were relaxed to a normal bond length by the end of the 1 μs MD simulation (Figure S1). Standard protonation states in Amber that correspond to a pH of 7 were used for all residues. We note that the aforementioned missing regions would be very difficult to model accurately due to their large size of 280 and 23–33 missing residues. As pointed out previously by Heinz et al. in their recent study on the RyR1 channel,^50^ the missing regions contain intrinsically disordered structural elements, which would explain their lack of appearance in the original cryo-EM structures. Overall, the compromises made to our models were similar to those made by Heinz et al.; in that study, a 300-residue region was omitted in the channel domain, and the ends of the missing sequence were tied together.^50^ However, it is important to note that the regions of interest that we are studying in this work are not located in the vicinity of these missing regions.

We also note that the residue numbers used throughout this paper are from the canonical human RyR2 residue numbering sequence (isoform 1, identifier: Q92736-1) provided on the Uniprot database^51^ (Figure S2), while the residue numbers listed within the original PDB files refer to a porcine residue numbering sequence and are one higher than the canonical RyR2 numbering system we use here (i.e., residue G4864 will be listed as G4865 within the PDB file and residue H4762 as H4763 within the PDB file, etc.). The total number of residues in our closed-state model system (4C) was 2124 (531 residues per subunit), while that in the open-state model system (4O) was 2120 (530 residues per subunit).

Each RyR2 system was placed inside an explicit membrane model using the CHARMM-GUI membrane builder^52^ and was converted for use with the Amber Lipid 14 force field as described in the Amber lipid membrane tutorial.^53^ For the closed 4C system, the membrane and RyR2 channel were placed in an ∼114 × 113 × 158 Å^3^ box with the protein–membrane system centered on the *z*-axis. The luminal side of the membrane contained 121 dipalmitoylphosphatidylcholine (DPPC) lipids, while the cytosolic side contained 103 DPPC lipids. DPPC lipids are a standard lipid type that is commonly used in MD simulations. For the open 4O system, an ∼126 × 126 × 153 Å^3^ box was similarly constructed. In this system, 168 DPPC lipids were added on the luminal side, and 139 DPPC lipids were added on the cytosolic side. A 0.15 M aqueous KCl solution with a TIP3P water model was used to fill the rest of the box for both systems using the CHARMM-GUI membrane builder.^52^ We note that the number of lipids and the solvent box size were the maximum values that we were able to input while still obtaining a usable structure from the CHARMM-GUI membrane builder. In particular, a limit was placed on the number of residues we could obtain while still obtaining a viable membrane model for the 4O system, and so we reduced the maximum number of residues in our 4C system to match those used in the 4O system.

To study RyR2 channel gating, we constructed several chimera RyR2 systems containing a mixture of structural elements from the closed 4C and open 4O model systems. The first chimera system consisted of replacing the first open subunit in our 4O model system with the first closed subunit from our 4C model system (1C3O). The two subunits were isolated from their respective PDB files, and an alignment of the first closed subunit from 4C to the first open subunit from 4O was performed using UCSF Chimera 1.15.^54^ The aligned first subunit from 4C was then inserted in place of the first open subunit in 4O, and this model system was used as the starting point for the 1C3O MD simulation. We note that the solvation box and lipid composition match the 4O system, as the only difference between 4O and 1C3O is the swapping of the first subunit in the RyR2 structure. While we were not free to create an arbitrarily large membrane model and solvation box size for our 4O system, we should note that any possible artifacts near the perimeter of our membrane would affect all of our systems equally, since the lipid composition and simulation box size in 4O and 1C3O were identical. We also note that the portion of the system that we were interested in was located in the central transmembrane domain, which was well within the simulation boundaries of our systems.

**Figure 2:**
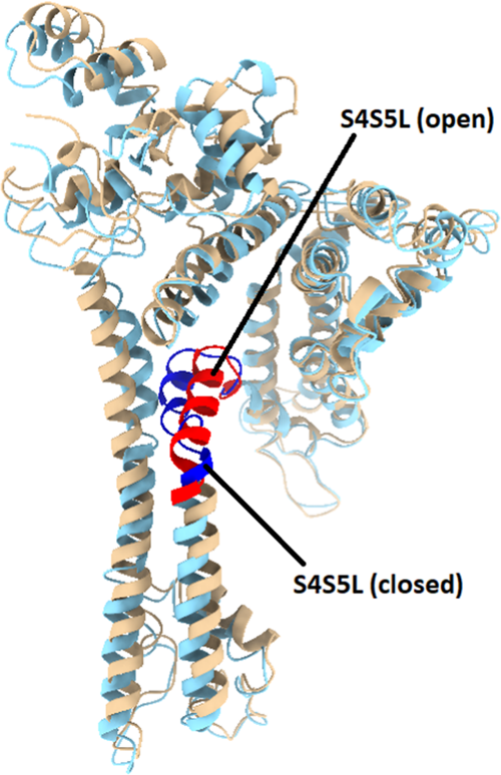
Initial alignment of the first closed RyR2 subunit with the first open RyR2 subunit. An alignment from the initial models of the first subunit in the closed 4C RyR2 model system (tan) and the first subunit in the open 4O RyR2 model system (light blue) before minimization and MD simulations were carried out. The S4S5L (residues 4746–4766) is highlighted for the closed RyR2 model system (blue) and for the open RyR2 model system (red). The alignment of the two subunits was performed using UCSF Chimera 1.15, while the images were visualized using UCSF Chimera X 1.4.

During the alignment process, we noticed that the first closed 4C subunit aligned surprisingly well with the first open 4O subunit, with an overall RMSD between the two subunits of about 3.2 Å. The biggest observable difference between the two aligned subunits occurred near the center of the pore in the transmembrane domain. This corresponded to a kink in the residue sequence 4746–4766, a sequence that included the S4S5L (Figure 2). To test the effect of changing the conformation of the S4S5L (residues 4746–4766) on the final state of the channel, we aligned the first closed RyR2 subunit with the first open RyR2 subunit as described above. Then, we deleted the residues corresponding to residues 4746–4766 in the first closed subunit from the 4C system and replaced them with residues 4746–4766 from the first open subunit in the 4O system. We avoided using modeling software at this stage because we wanted to preserve the remainder of the original conformation of the closed subunit and the original conformation of the S4S5L region from the open subunit as much as possible. To minimize potential issues using a direct substitution, we chose the extent of the S4S5L region to be long enough for the edges to be in similar positions between the closed and open states (Figure 2). The final structures after minimization were examined by a thorough manual inspection to confirm that there were no unphysical binding contacts present. This model system was used as the starting point for a MD simulation (1C3O-open-S4S5L).

We further tested for the effect of the H4762P mutation using Modeller 1.17^55,56^ to introduce the mutation into the first subunit of each of the two chimera systems described above (1C3O-H4762P and 1C3O-open-S4S5L-H4762P, respectively). One possible issue with basing our conclusions on this particular point mutation is that a change in the state of the channel might be due to more subtle issues with the positioning of protons within the histidine residue rather than a defect brought about by replacing the histidine residue as a whole with a proline residue. To check the stability of our chimera system with respect to more subtle changes made to its structure, we examined the smallest change we could make to the H4762 residue in the first subunit from the 4C structure. This was accomplished by switching the H4762 protonation state from the epsilon position to the delta position for two additional runs (1C3O-HID and 1C3O-open-HID). A summary of all eight RyR2 systems along with their abbreviated designations is provided in Table 1.

**Table 1:** RyR2 Model Systems^a^

model	initial state before the MD simulation
4C	all four subunits in the closed conformation
4O	all four subunits in the open conformation
1C3O	one subunit in the closed conformation, other three subunits open
1C3O-open-S4S5L	one subunit in the closed conformation except for the S4S5L (open conformation), other three subunits open
1C3O-H4762P	one subunit in the closed conformation except for the H4762P mutation, other three subunits open
1C3O-HID	one subunit in the closed conformation except for H4762 (HIE) changed to (HID), other three subunits open
1C3O-open-S4S5L-HID	one subunit in the closed conformation except for the S4S5L (open conformation) and H4762 (HIE) changed to (HID), other three subunits open
1C3O-open-S4S5L-H4762P	one subunit in the closed conformation except for the S4S5L (open conformation) and the H4762P mutation, other three subunits open

a A description of all eight RyR2 model systems is provided along with their abbreviated designations. The 4C model is based on a closed-state model of the RyR2 system (PDB ID: 6JI8). The closed subunit used in the chimera (1C3O) systems is the first subunit from the 4C model. The 4O model is based on the open-state cryo-EM model of the RyR2 system (PDB ID: 6JIY). The other three subunits used in the chimera systems are subunits 2–4 in the 4O model.

### MD Simulation Protocol

We employed the Amber 19 software suite^57,58^ for our MD simulations. Addition of hydrogen atoms and terminal residue designations were handled by Leap using the default residue types in Amber. We employed a standard MD protocol where the basic simulation settings for each step have been made available to the general public in the Amber Lipid Membrane Tutorial.^52^ A nonbonded cutoff of 10.0 Å was employed throughout. Before starting MD, a 10,000-step minimization was carried out using 5000 steps of steepest descent followed by 5000 steps of conjugate gradient on each system. The following MD simulations all employed a 2 fs timestep with the SHAKE algorithm^59^ applied to all hydrogen atoms. The system was heated from 0 K to ∼100 K for 5.0 ps using the Langevin thermostat in the NVT ensemble. The system was then heated from 100 to 303 K for 100 ps by employing the Langevin thermostat in the NPT ensemble while using anisotropic pressure scaling and a pressure relaxation time of 2.0 ps. Density equilibration was then carried out for 500 ps, and finally, trajectory data was collected for an additional 1 μs.

After each MD simulation was complete, 1000 equally spaced frames (1 frame/ns) from the original trajectory were retained for analysis of the RMSD as a function of time (Figures S3 and S4). For diagnostic purposes, we also looked at the minimum radius calculated using the Hole software over the course of the trajectory (Figures S5 and S6). In this new trajectory, all solvent molecules such as water, lipids, and counterions were removed, leaving only the RyR2 channel to be analyzed. For the subsequent channel pore radius and RMSD structure analysis, we analyzed an average structure produced from the last 100 frames of this new trajectory, which corresponded to an average over the last 100 ns of the 1 μs MD simulation. This was accomplished using cpptraj in the Amber software suite.^57,58^ A rms fit of all 100 frames to the first frame was first carried out to remove global translational and rotational degrees of freedom, and the atomic coordinates were subsequently averaged to produce a single average structure file. While this average structure may appear distorted in regions where the structure is highly flexible, this approach allows for a simple comparison between different ensembles of systems in terms of their well-ordered structural elements while using just a single structure to aid in the visualization process.

### Pore Radius Analysis

We analyzed the RyR2 channel pore radius profile using the Hole software.^60^ For six out of our eight model systems, we specified a cutoff radius of 10 Å when calculating the extent of the channel pore. For two of our systems (4O and 1C3O-open-S4S5L), we increased the cutoff radius to 12 Å, as the radius of the pore went just over 10 Å in a portion of the channel domain on the cytosolic end.

### RMSD Structure Analysis

We also examined the RMSD of the structures in comparison to the original closed 4C system, which was arbitrarily chosen to be our reference system. This was accomplished using Match Maker with the default settings in UCSF Chimera 1.15 to align each structure using the best aligned pair of chains with reference to the closed 4C system.^54^ The RMSD of the fit was then reported within the software. For analyzing isolated substructural RyR2 elements, a PDB file was constructed containing only the specific residue sequence on all four subunits; residues not included in the sequence from each of the four subunits in the original PDB file were deleted.

To compare the alignment of a certain residue sequence in the absence of other structural elements of the RyR2, each PDB file was first modified to contain only the listed residues in all four subunits that were being compared. We had to include all four subunits in each comparison because the area and pore radius of the hydrophobic gate within each structure determine the observed state of the channel, and this area is not defined in the comparison unless all four subunits are included. To correlate our structural comparisons with the global state of the channel, we therefore must maintain the relationship of all four subunits together in three-dimensional space in all of our comparisons. The alignment was performed using UCSF Chimera 1.15, where the best aligned pair of chains was used as the alignment criterion.^54^ After the alignment, the software reported the overall RMSD for the fit of the entire four-subunit structure.

### High Percent Occupancy Interaction Analysis

To identify the key residue–residue interactions at the interface of the S4S5L with the surrounding protein environment, we used cpptraj in Amber 19 to report the percent occupancy of heavy atom (nonhydrogen) contacts that appeared over all 100 frames covering the last 100 ns of our MD trajectory. We used a standard distance-based cutoff approach where a cutoff distance of 3.0 Å between heavy atoms was specified. This cutoff value was chosen to be close to the mean cutoff distance of hydrogen bond donor–acceptor distances in common protein secondary structures.^61^ We took the percent occupancy of each individual interaction as the number of frames where that interaction appeared with respect to the total number of frames analyzed in our trajectory. To identify the most important interactions involved in binding, we analyze only those interactions with a percent occupancy ≥30%, which we refer to as high percent occupancy interactions. This entire procedure was repeated to identify the key residue–residue interactions that appear at the subunit–subunit interface. In our analysis, we pooled data together from all four subunits for the three closed-state RyR2 systems (4C, 1C3O, and 1C3O-HID) and compared it to the pooled data from all four subunits for three open-state RyR2 systems (4O, 1C3O-open-S4S5L, and 1C3O-open-S4S5L-HID). We pooled data for the two mutant systems (1C3O-H4762P and 1C3O-open-S4S5L-H4762) separately. While the systems were not identical, the observed end state for the systems being pooled was the same, and this approach allowed us to present the high percent occupancy interaction locations in a very simple way for our analysis. The full data set for all eight model systems before we pooled the data is available in Tables S1–S4.

## Results

### All 4 S4S5L are required to be in an Open Conformation in the Open State of RyR2

**Figure 3:**
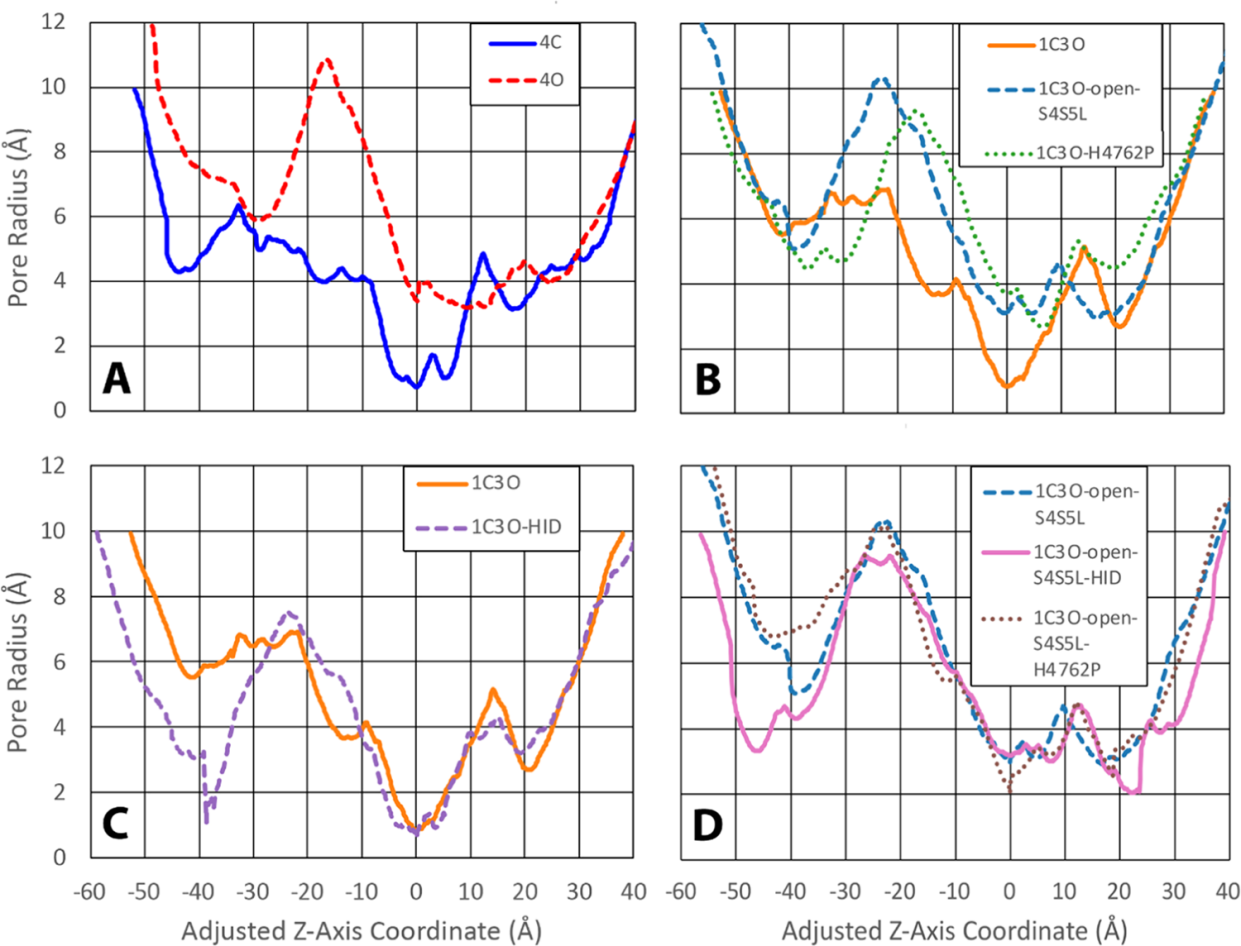
Pore radius profiles of RyR2 systems. A numerical plot of the pore radius as a function of the *z*-coordinate is provided for the structures of the eight RyR2 model systems. The single structure analyzed in these plots was obtained by averaging across 100 snapshots sampled every 1 ns over the last 100 ns of the MD simulation. The pore radius profiles of the original closed 4C and open 4O systems are given in panel (A), the pore radius profiles of the chimera systems 1C3O, 1C3O-open-S4S5L, and 1C3O-H4762P are given in panel (B), the pore radius profile of the chimera system 1C3O-HID is given in panel (C) along with the 1C3O system for ease of comparison, and the pore radius profiles of chimera systems 1C3O-open-S4S5L-HID and 1C3O-open-S4S5L-H4762P are given in panel (D) along with the 1C3O-open-S4S5L system for ease of comparison. To facilitate comparisons between the various systems, the origin of the *z*-axis was adjusted for all eight systems so that the zero point corresponded to the hydrophobic gate. Positive *z*-values indicate the luminal side of the channel and negative values appear on the cytosolic side. Visualizations of the channel and pore for these plots are available in Figures S7–S10.

We first examined the final state of the closed 4C and open 4O RyR2 model systems that were based on the original cryo-EM structure files (PDB IDs 6JI8 and 6JIY, respectively). As with the study by Nury et al.,^47^ we observed that most of the changes in the pore radius occurred by 50–400 ns. This was typically followed by a slower conformational drift for 400−900 ns and then equilibration for the last 100 ns of the 1 μs simulation (Figures S5 and S6). To assess the equilibration, RMSD plots are provided in Figures S3 and S4. It can be seen in Figures S3 and S4 that our systems had already reached a plateau in the RMSD at ∼600 to 800 ns into the simulation. Based on this observation, a single structure corresponding to an average of the fully equilibrated structure over the last 100 ns of the MD simulation was analyzed for each system. In Figure 3A, we have provided the pore radius profile of both the 4C and 4O systems using the Hole software.^60^ To facilitate comparisons between RyR2 systems, the origin of the *z*-axis in Figure 3 was adjusted so that the zero point corresponded to the hydrophobic gate. In Figure 3, positive *z*-values indicate the luminal side of the channel and negative *z*-values appear on the cytosolic side. Visualizations of both the 4C and 4O models are available in Figure S7A–D.

**Figure 4:**
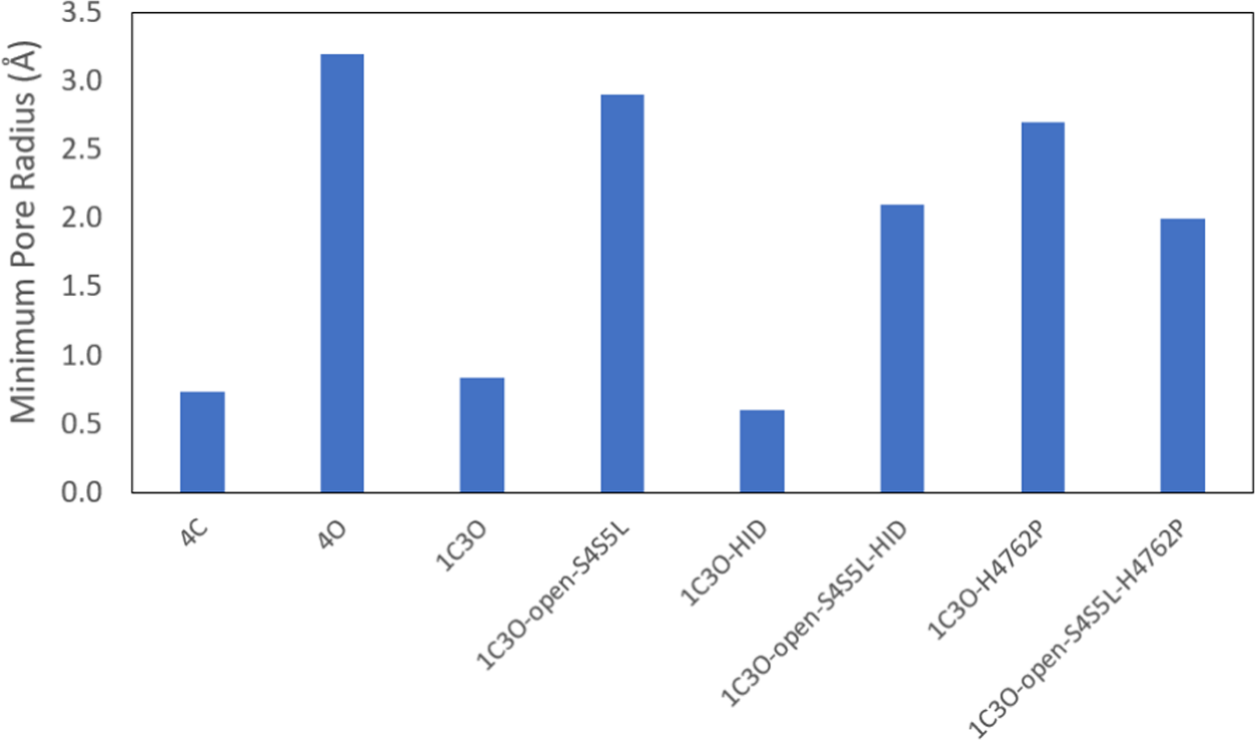
Minimum pore radius of RyR2 systems. A comparison of the minimum pore radius is provided for all eight RyR2 model systems. For the three closed-state systems (4C, 1C3O, and 1C3O-HID) and one open-state system (1C3O-open-S4S5L-H4762P), the minimum pore radius corresponded to the hydrophobic gate formed by four I4867 residues. For the remaining four open-state systems (4O, 1C3O-open-S4S5L, 1C3O-open-S4S5L-HID, and 1C3O-H4762P), the minimum pore radius is found within the luminal domain, between *z* = 0 and 25 Å (see Figure 3).

The 4C model, based on the closed structure of RyR2, remained closed at the end of the MD simulation. The 4C model had a minimum pore radius of 0.74 Å that was located at the central, hydrophobic gate of the channel (Figures 3A and 4). The 4O model, based on the open structure of RyR2, remained open at the end of the MD simulation and had a pore radius of 3.4 Å at the central hydrophobic gate of the channel (Figure 3A). For the 4O system, a minimum pore radius of 3.2 Å appeared in the luminal region, at about *z* = 9 Å (Figures 3A and 4).

The 4C and 4O models could be further distinguished from each other by a pronounced difference in their pore radius profiles in the cytosolic region, from *z* = 0 Å to about *z* = −20 Å (Figure 3A). It can be seen from Figure 3A that the 4O model maintained a positive difference above the 4C model of between 2 and 6 Å along the pore axis throughout this 20 Å region. Beyond 20 Å, the pore radius gradually diminished to about the same size as 4C by *z* = −30 Å. These distinguishing features of the open and closed RyR systems in the vicinity of the central hydrophobic gate have been described previously in the literature.^29,30,32,33^

We now examine what happened when we substituted the first subunit from the 4C model in place of the first subunit of the 4O model (1C3O) at the start of the 1 μs MD simulation. The simulation was similar to the 4C and 4O systems in that we observed that the pore radius had stopped noticeably changing by 400 ns, and the simulation was extended out to 1 μs to further relax the system. This generally remained the case for all subsequent replicas based on the 1C3O system (see Figures S3–S6). The pore radius profile of the 1C3O system is provided in Figure 3B. In comparing Figure 3B to the pore radius profiles given in Figure 3A, we see that the insertion of a single closed 4C subunit into a model containing three open-state 4O subunits closed the entire channel. The 1C3O channel is visualized in Figure S8A,B, demonstrating that the pore is constricted. In 1C3O, a minimum pore radius of 0.84 Å was found at the central hydrophobic gate (Figures 3B and 4). In Figure 3B, we also note that the pore radius for 1C3O rises to about 4 Å in the range of 0 to −12 Å analogous to the 4C state (Figure 3A). One difference we observed was that the pore radius in 1C3O rose more sharply, to about 6 Å between −12 and −30 Å, compared to the 4C state, which rose to 6 Å more gradually with distance from the hydrophobic gate. Nevertheless, the 6 Å pore radius in this region for 1C3O (Figure 3B) was still 2–3 Å smaller than the 8–11 Å pore radius observed in the 4O state between −10 and −20 Å (Figure 3A).

Having observed that inserting the first subunit of the 4C system in place of the first subunit in the 4O system could close the entire channel within a 1 μs timeframe, we now wanted to examine the role of the S4S5L on the first subunit in controlling this open-to-closed transition. To this end, we repeated our previous setup for the 1C3O system, but we now inserted residues 4746–4766 (which included the S4S5L), extracted from the first 4O subunit, in place of the 4746–4766 residues originally present in the first closed subunit (Figure 2) before carrying out the 1 μs MD simulation (1C3O-open-S4S5L). The idea was that everything in the first closed subunit would be in the same initial conformation as in the 1C3O system except for the S4S5L (residues 4746–4766), which had the same starting conformation as it had in the first subunit of the 4O system.

Figure 3B reveals that the 1C3O-open-S4S5L system remained open at the end of the 1 μs MD simulation. The 1C3O-open-S4S5L system is visualized in Figure S8C,D, showing clearly that the pore is dilated. A pore radius of 3.0 Å was observed at the hydrophobic gate of the channel. As was the case with the 4O system, this was not the global minimum; the global minimum for the 1C3O-open-S4S5L system was 2.9 Å and appeared at *z* = ∼17 Å within the luminal portion of the channel (Figures 3B and 4). The observed pore radius of 8–11 Å between −10 and −30 Å for 1C3O-open-S4S5L is comparable to the increased pore radius observed for the 4O system (Figure 3A,B). Since everything except for the S4S5L (residues 4746–4766) in the first subunit was identical to the initial configuration of the prior 1C3O system, the 1C3O-open-S4S5L result suggested that the initial conformation of the S4S5L played a critical role in determining whether or not the RyR2 channel remained open or closed at the end of our 1 μs MD simulation.

We wanted to test the sensitivity of changes made to the S4S5L in the first subunit of the 1C3O system by introducing a smaller change to the S4S5L, at the level of an individual amino acid residue. For this, we introduced a single CPVT point mutation, H4762P,^6,12^ into the first closed subunit while leaving everything else the same as in the preparation of the 1C3O system (1C3O-H4762P). In this case, the conformation of the S4S5L (residues 4746–4766) in the 1C3O-H4762P system would be similar to the initial conformation of the 1C3O system except in the near vicinity of residue 4762, where the conformation would have to adjust slightly to accommodate the point mutation.

Figure 3B reveals that the 1C3O-H4762P system remained open at the end of the 1 μs MD simulation. The 1C3O-H4762P system is visualized in Figure S8E,F, which shows the dilation of the pore. For 1C3O-H4762P, a pore radius of 3.7 Å appeared at the hydrophobic gate. Like the two previous open-state structures, this did not correspond to the global minimum, which was 2.7 Å and appeared at about *z* = 7 Å within the luminal region (Figures 3B and 4). The region between −10 and −20 Å was similar to 4O and 1C3O-open-S4S5L as the pore radius rose to a range of 8–11 Å in this region (Figure 3A,B).

We further tested the sensitivity by introducing a minimal change within the S4S5L. The histidine residue at position 4762 has two possible positions for a hydrogen atom, and one possibility is that this subtle change may be responsible for the differences we observe, as opposed to a change induced by the mutation as a whole. To examine this possibility, we changed only the position of a single hydrogen atom within the histidine residue at position 4762. The hydrogen atom was shifted from the epsilon position (HIE) to the delta position (HID) in the first subunit of the 1C3O model while initially leaving everything else identical to the 1C3O system (1C3O-HID). In this case, the change in the position of a hydrogen atom on H4762 was insufficient to change the overall outcome from the 1C3O simulation, and the channel remained closed at the end of the 1 μs MD simulation, as seen in Figure 3C. The 1C3O-HID system is visualized in Figure S9C,D, confirming that the pore was closed. The 1C3O and 1C3O-HID pore profiles were very similar from −20 to 40 Å with a minimum radius of 0.61 Å reached at the central hydrophobic gate in the latter case. One major difference can be observed between *z* = −30 and −60 Å in the central domain region containing the U-motif, where the radius in the 1C3O-HID simulation dipped down between 1 and 6 Å. This change did not appear in the vicinity of H4762, which is located within the transmembrane domain. This was well below the minimum of 6 Å observed in this region in the original 1C3O simulation. However, the pore profile from *z* = −30 to 40 Å in the transmembrane domain is comparable between 1C3O-HID and 1C3O. Due to our large system size, our ability to replicate our results in this study was limited, and so one useful result was that the similarity in our results in the transmembrane domain between our 1C3O-HID and 1C3O systems showed that the ability of the first subunit from 4C to induce closing when it replaces the first subunit of 4O was reproducible.

In our open-state chimera system, 1C3O-open-S4S5L, we tested for the effects of repositioning the hydrogen atom within residue 4762 and the H4762P mutation as we did for the closed-state 1C3O system. We first replicated the 1C3O-open-S4S5L system except for changing the hydrogen atom from epsilon to delta at position 4762 (1C3O-open-S4S5L-HID). As seen in Figure 3D, this change did not appreciably change the outcome from the 1C3O-open-S4S5L simulation as both channels remained open at the end of 1 μs. A visualization of the 1C3O-open-S4S5L-HID system is given in Figure S10C,D, confirming that the pore was dilated in comparison to the 4C, 1C3O, and 1C3O-HID systems. A pore radius of 3.2 Å was observed at the central hydrophobic gate in 1C3O-open-S4S5L-HID, and the pore radius profile was comparable with 1C3O-open-S4S5L between *z* = −40 and 40 Å. There were a few notable differences in the 1C3O-open-S4S5L-HID system compared to the 1C3O-open-S4S5L system. The 1C3O-open-S4S5L-HID system showed a dip of 2–4 Å in its pore radius between −40 and −50 Å in the central domain region containing the U-motif, in comparison to the 1C3O-open-S4S5L system, and there was a similar dip in the minimum pore radius to 2.1 Å that appeared at *z* = 24 Å (Figures 3D and 4). Neither change appeared in the vicinity of H4762, which is located within the transmembrane domain. However, the pore profile as a whole is more comparable to the open-state systems in Figure 3D than to the closed-state systems in Figure 3C. We conducted one final MD simulation where we introduced the H4762P mutation into the 1C3O-open-S4S5L system (1C3O-open-S4S5L-H4762P). The pore profile of the 1C3O-open-S4S5L-H4762P system is given in Figure 3D. The 1C3O-open-S4S5L-H4762P system is visualized in Figure S10E,F, confirming that the pore was dilated. As shown in Figure 3D, the end result was more comparable to 1C3O-open-S4S5L and 1C3O-open-S4S5L-HID than to the closed-state systems in Figure 3C. The 1C3O-open-S4S5L-H4762P had a minimum pore radius of 2.0 Å, which was located at the hydrophobic gate at *z* = 0 Å (Figures 3D and 4). This was also the smallest minimum pore radius of the five open-state systems (Figure 4). Nevertheless, the rise in the pore radius between *z* = 0 and −40 Å in the pore profile in Figure 3D was more comparable to the other open-state systems than to the closed-state systems in Figure 3C. In addition, the similarity in the transmembrane domain between these various systems shows that our results for the open-state chimera systems are also reproducible.

### S4S5L in a Closed Conformation Determines the Closed State of RyR2

In addition to analyzing the pore radius profile of each RyR2 structure, we also compared how well the conformation of each structure aligned with the conformation of the closed 4C structure. Our goal was to see which global structural elements were conformationally similar between closed-state systems when compared to the open-state systems. We note that the RMSD analysis used to determine equilibrium in Figures S3 and S4 cannot be used for this purpose as the trajectory for each system in Figures S3 and S4 is referenced to the first frame of its own trajectory and not to a common reference point. Here, we arbitrarily chose the 4C system to use as our common reference point for all structures.

To compare the alignment of a certain residue sequence in the absence of other structural elements of the RyR2, each PDB file was first modified to contain only the listed residues in all four subunits that were being compared. To correlate our structural comparisons with the global state of the channel, we must maintain the relationship of all four subunits together in three-dimensional space in all of our comparisons. The alignment was performed using UCSF Chimera 1.15, where the best aligned pair of chains was used as the alignment criteria.^54^ After the alignment, the software reported the overall RMSD for the fit of the entire four-subunit structure.

Table 2 gives the RMSD values reported for an alignment of various structural elements of the eight RyR2 models with respect to the 4C system. In Table 2, the determination of the open or closed state is clearly correlated with the conformation of residues within the S4S5L (residues 4731–4781, and in particular, within residues 4746–4766). For the larger S4S5L structure corresponding to residues 4731–4781, the two closed chimera systems had the smallest RMSD values in comparison to 4C of all of the chimera structures, with RMSD values of 1.1 and 0.5 Å for the 1C3O and 1C3O-HID systems, respectively. The next smallest RMSD belonged to the 1C3O-open-S4S5L-H4762P system that had an RMSD of 3.4 Å. When the S4S5L structure was narrowed to just the 4746–4766 residues, similar results were obtained, with the two lowest RMSD values of 0.7 and 0.4 Å again belonging to the two closed-state chimera systems. We also note in Table 2 that the correlation with the observed state of the system is much stronger for the conformation of the S4S5L structure than it is for other structural elements that we examined.

**Table 2:** RMSD Values of RyR2 Structural Features^a^

	full model	central domain	transmembrane	S4S5L		S6 helix	
	4099–4963	4099–4206	4485–4963	4731–4781	4746–4766	4839–4889	4859–4869
4C	0.0	0.0	0.0	0.0	0.0	0.0	0.0
4O	7.2	5.0	5.7	8.5	5.1	3.0	0.3
1C3O	5.0	6.1	8.0	1.1	0.7	1.5	0.3
1C3O-open-S4S5L	8.7	4.7	3.9	5.0	6.1	2.7	0.3
1C3O-H4762P	8.4	6.4	5.9	6.1	5.2	2.1	0.2
1C3O-HID	6.2	7.2	3.6	0.5	0.4	1.6	0.1
1C3O-open-S4S5L-HID	6.3	5.4	5.8	4.3	3.4	1.1	0.3
1C3O-open-S4S5L-H4762P	7.2	5.8	7.5	3.4	5.6	3.5	0.2

a The RMSD values of an alignment of various structural features in each model system with reference to the 4C system are shown. Only the stated residue sequence on each of the four RyR2 subunits was retained for each comparison; the remaining residues were removed from the PDB files before the alignment. The residue sequences correspond to: (1) our full model (residues 4099–4206 and 4485–4963), (2) the central domain/U-motif region (residues 4099–4206), (3) the transmembrane domain region (residues 4485–4963), (4) a 51-residue region that included the S4S5L (residues 4731–4781), (5) the S4S5L region that was transposed from the open to the closed state subunits when producing the 1C3O-open-S4S5L chimera models (residues 4746–4766), (6) a 51-residue region along the S6 helical bundle that included the central hydrophobic gate (residues 4839–4889), and (7) an 11-residue portion of the S6 helical bundle that included the central hydrophobic gate (4859–4869). A visual comparison of the aligned structures for the S4S5L (residues 4731–4781) is provided in Figure S11. The alignment and RMSD values were obtained using UCSF Chimera 1.15.

Figure S11 gives a visual depiction looking down from the cytosolic end of the loop formed from the S4S5L (residues 4731–4781) for each model RyR2 system with respect to the 4C system. In Figure S11, it can be observed that there is a subtle twist in the relative position of the four-subunit S4S5L gating loop that occurs upon channel opening as described previously by Peng et al.^32^ and Nury et al.^47^ This twist in Figure S11 can be used to distinguish the closed-state structures from the open-state structures, as the four helices that stick out from the central loop of the closed-state chimera structures align better with the original 4C loop in Figure S11B than with the open-state chimera structures in Figure S11C,D.

However, the subtlety of this twist in our chimera structures, and the fact that our comparisons do not isolate the key residue–residue interactions that determine the key conformational changes on each individual subunit, made it difficult to visually determine which elements of the S4S5L conformation in a given subunit were responsible for the observed differences between the various closed and open RyR2 systems. For example, we were looking for a noticeable kink induced by the H4762P mutation on the first subunit that brought the conformation of the S4S5L in the 1C3O-open-H4762P system into agreement with the 4O system, but the various chimera systems in Figure S11 all appeared very similar to each other. This similarity suggested that a more detailed analysis at the level of individual residue–residue binding interactions was required. For this, we switched to a different method that we had used previously to study residue–residue binding interactions in the RyR2-CaM system.^62^

### S4S5L Interacts with the S6 Helix and the U-Motif

**Figure 5:**
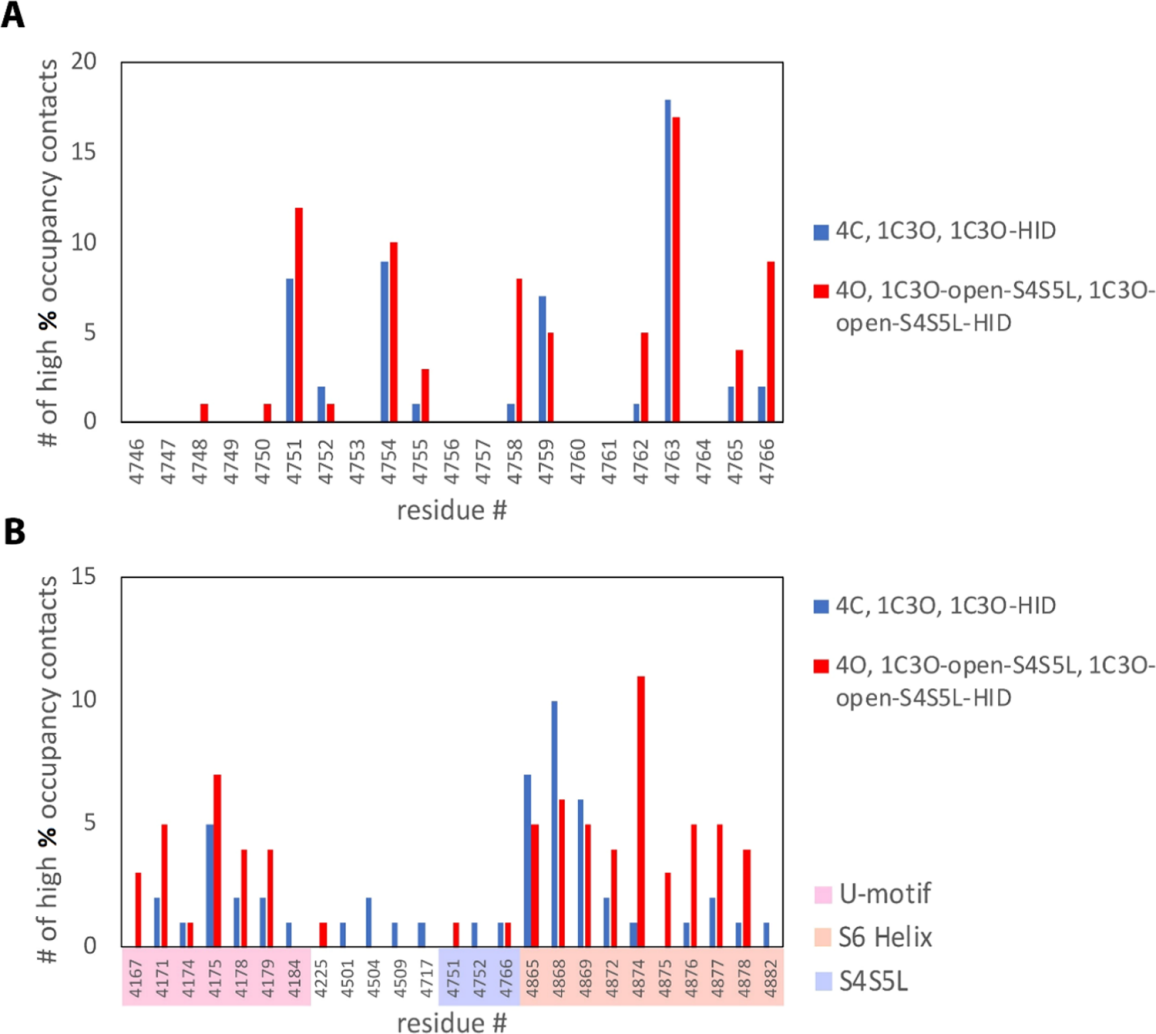
Number of S4S5L high percent occupancy interactions observed in the closed and open RyR2 systems. The number of high percent occupancy interactions involving the S4S5L (residues 4746–4766) observed by residue number within our three closed (4C, 1C3O, and 1C3O-HID) and three open (4O, 1C3O-open-S4S5L, and 1C3O-open-S4S5L-HID) RyR2 systems is given. The number of interactions at a given residue position within the S4S5L is given in panel (A), while the number of interactions at each residue position that the S4S5L interacted with is given in panel (B). In producing this figure, we pooled data from all four subunits in the three closed RyR2 systems (4C, 1C3O, and 1C3O-HID) and compared it to the pooled data from all four subunits in three open RyR2 systems (4O, 1C3O-open-S4S5L, and 1C3O-open-S4S5L-HID). In panel (B), residue numbers for regions within the U-motif, S4S5L, and the S6 helix that we focus on in our discussion are highlighted along the *x*-axis. The full set of high percent occupancy interaction data is available in Tables S1 and S2.

To provide a more detailed analysis of the interactions of the S4S5L with its surrounding environment, we located the primary residue–residue interactions involved in the binding of the S4S5L on a given subunit to its surrounding protein environment within the RyR2. We then compared these interactions in the closed- and open-state RyR2 systems. Using the last 100 ns of our 1 μs MD trajectory, for each frame, we identified each heavy atom (nonhydrogen) contact within a 3.0 Å distance cutoff between a S4S5L residue (within residues 4746–4766) and another residue within the RyR2 structure. We calculated the percent occupancy of each individual residue–residue interaction as the number of frames where that interaction appeared with respect to the total number of frames analyzed in our trajectory. To identify the most important interactions involved in binding, here we only analyze those interactions with a percent occupancy ≥30%, which we refer to as high percent occupancy interactions. In Figure 5, we pooled data together from all four subunits for the three closed-state RyR2 systems (4C, 1C3O, and 1C3O-HID) and compared it to the pooled data from all four subunits for the three open-state RyR2 systems (4O, 1C3O-open-S4S5L, and 1C3O-open-S4S5L-HID). This was done to simplify the analysis of Figure 5, as our goal here was merely to identify the key interaction locations. To allow for a deeper analysis of variations between binding interactions on the individual subunits and between the different systems used in our analysis, the full data set of the high percent occupancy interactions that were used to construct Figure 5 has been provided in Tables S1 and S2.

Figure 5A reveals that, within the S4S5L (residues 4746–4766), there were certain key residue locations where the high percent occupancy interactions were most readily found in both the open and closed RyR2 systems. The four most prominent interactions in both the closed and open states were K4751, R4754, S4759, and N4763. Additionally, S4758, H4762, and Q4766 were prominent interactions in the open state, but not in the closed state. Of these sites, the most prevalent high percent occupancy interaction site is N4763 in both the open and closed systems. We note that N4763 is located directly adjacent to H4762 in the S4S5L.

Figure 5B shows the key residues located elsewhere in the RyR2 that were bound to the S4S5L residues listed in Figure 5A. In Figure 5B, we can identify two key binding domains where the majority of high percent occupancy interactions were found in both the closed and open systems. These are residues located within the U-motif (residues 4167–4184) and within the S6 helix (residues 4865–4882). When comparing the closed and open RyR2 systems, we see that the biggest difference occurred in binding to the S6 helix. In the closed-state systems, prominent high percent occupancy interactions between the S4S5L and the S6 helix appeared between residues 4865–4869 within the S6 helix, with the most prominent being at D4868. Interactions also appeared between these residues in the open-state systems, but a noticeable increase in the number of high percent occupancy interactions between the S4S5L and the S6 helix was observed between residues 4870 and 4880 within the S6 helix in the open-state systems compared to the closed-state systems. The most prominent of these interactions was residue R4874, toward the middle of this region, which was considerably more common to find in the open systems versus the closed systems.

We note that we have omitted the two open-state mutant systems from Figure 5 (1C3O-H4762P and 1C3O-open-S4S5L-H4762P). This was done so that we could compare the raw interaction counts for each residue using the same number of systems in Figure 5 (three closed and three open). A similar analysis is provided for the two mutant systems in Figure S12. The trends of the two mutant systems are qualitatively similar to what was observed in the other three open systems. Notably, the shift toward an increased number of interactions in the S6 helix (residues 4870–4880) region in the open systems in Figure 5 is clearly present in the mutant systems in Figure S12 as well.

Additionally, we note that one limitation of pooling data for Figure 5B was that there was no longer a distinction between the S4S5L on one subunit and another residue on the same subunit (an intra-subunit interaction) and residue locations between the S4S5L on one subunit and a residue on an adjacent subunit (an inter-subunit interaction). This was why we saw a few interactions listed in Figure 5B for residues K4751, T4752, and Q4766, all of which were a part of the S4S5L sequence. In these cases, these were residues involved in interactions that took place between the S4S5L on one subunit and the S4S5L on an adjacent subunit. The same was also true in general of the shift in binding from residues 4865–4869 to the additional interactions appearing within residues 4870–4880 in the open systems. Frequently, these new interaction partners in the 4870–4880 sequence in the open-state systems were on a different subunit than the S4S5L it was partnered with. Intra- and inter-subunit interactions for each interaction pair are clearly distinguished in the data tables given in Tables S1 and S2.

**Figure 6:**
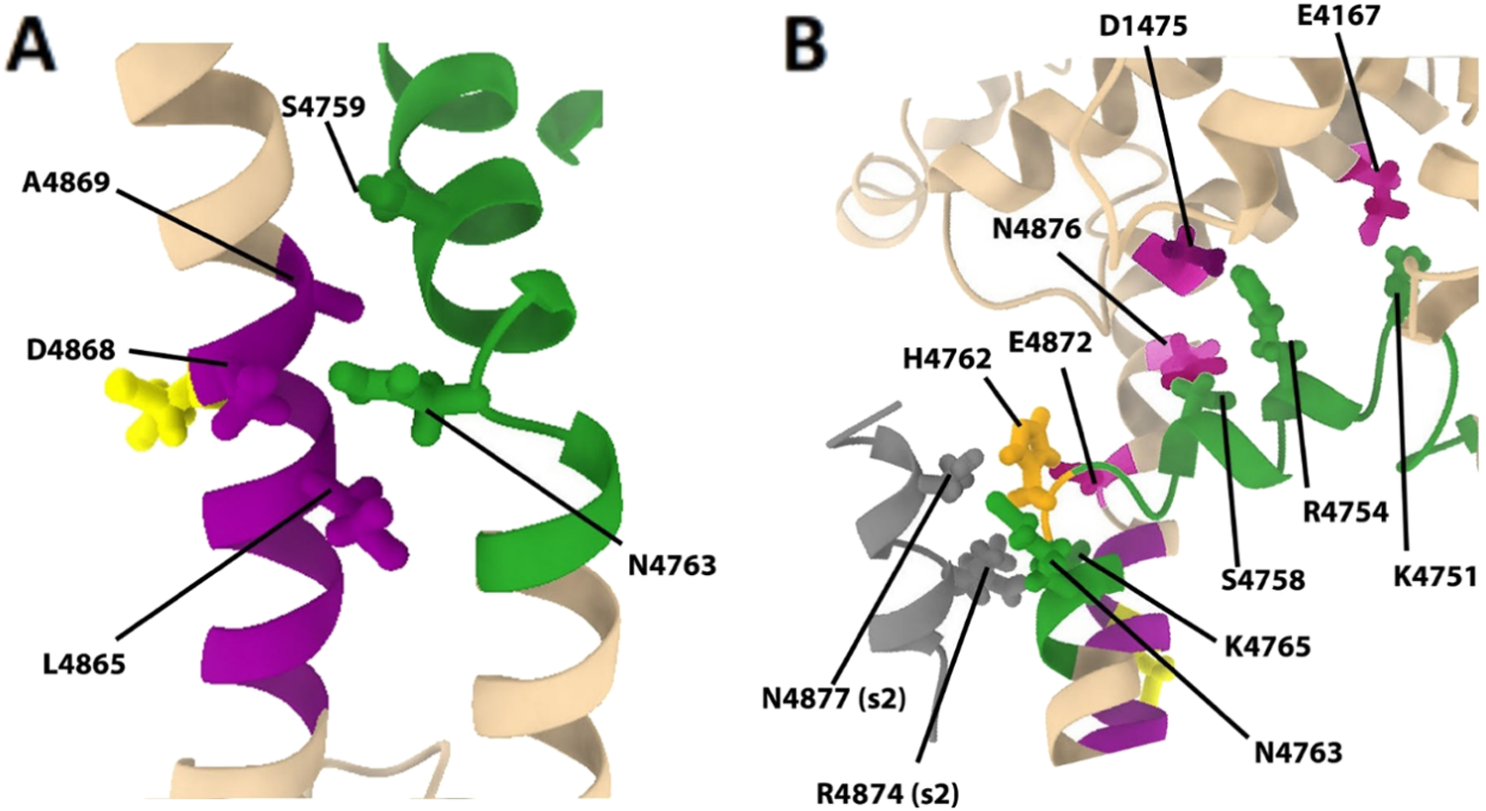
High percent occupancy S4S5L interactions in the 4C and 4O systems. S4S5L residues involved in high percent occupancy interactions are given for 4C (A) and 4O (B). The S4S5L (residues 4746–4766) is colored green, with H4762 colored orange. A portion of the S6 helix (residues 4859–4869) is colored purple, with I4867 colored yellow. Key interactions outside of these two regions are colored magenta when they appear on subunit 1. A portion of the adjacent subunit 2 (residues 4869–4879) is given in gray, while high percent occupancy interactions with subunit 2 are labeled (s2) for clarity. Both structures for 4C and 4O represent an average structure over the last 100 ns of the 1 μs MD simulation. This image was produced using UCSF Chimera X 1.4.

As an illustrative example, in Figure 6, we examine the key high percent occupancy interactions that appeared within the first subunit of the 4C system (Figure 6A) and within the first subunit of the 4O system (Figure 6B). In the first subunit of the closed 4C system, the key high percent occupancy interactions that were identified were three intra-subunit interactions between the S4S5L and the S6 helix. These were S4759–A4869, N4763–L4865, and N4763–D4868 (Figure 6A). We note that these three interactions were located in a group directly adjacent to a proposed hinge residue G4864 on the S6 helix.^35^ Of these, the only polar–polar sidechain interaction was N4763–D4868, and both of these residues were listed as very prominent interactions in Figure 5. D4868 also appeared directly adjacent to residue I4867 on the S6 helix, which is the residue that forms the hydrophobic gate. These intra-subunit high percent occupancy interactions were found on all four subunits in the 4C system, and no high percent occupancy inter-subunit interactions were identified for the 4C system (Table S1). Together, these interactions demonstrate the direct intra-subunit coupling of the S4S5L to the S6 helix in the closed 4C system.

The high percent occupancy interactions were noticeably different in the first subunit in the open 4O system. In the 4O system, we found more prominent S4S5L and S6 helix interactions in the 4870–4880 region, with intra-subunit interactions S4758–Q4876 and H4762–E4872 replacing the intra-subunit S6 helix interactions observed in the first subunit of the 4C system. In addition, the S4S5L formed two inter-subunit interactions to residues within the S6 helix (residues 4870–4880) region on the second adjacent subunit, with N4763–R4874 and K4765–Q4877 (Figure 6B). Importantly, the intra-subunit N4763–D4868 interaction in the closed 4C system had now been replaced with the inter-subunit N4763–R4874 interaction on the S6 helix of the adjacent subunit in the open 4O system.

We also note that in the first subunit of 4O, there were two additional intra-subunit high percent occupancy interactions between the S4S5L and the U-motif. These were K4751–E4167 and R4754–D4175 (Figure 6B). In general, interactions between the U-motif and S4S5L were commonly observed in both closed and open subunits as can be seen in Figure 5, but these interactions were more frequently seen in the open state as opposed to the closed state.

### Key Inter-Subunit Interactions on the S6 Helix are Disrupted in the Open State

**Figure 7:**
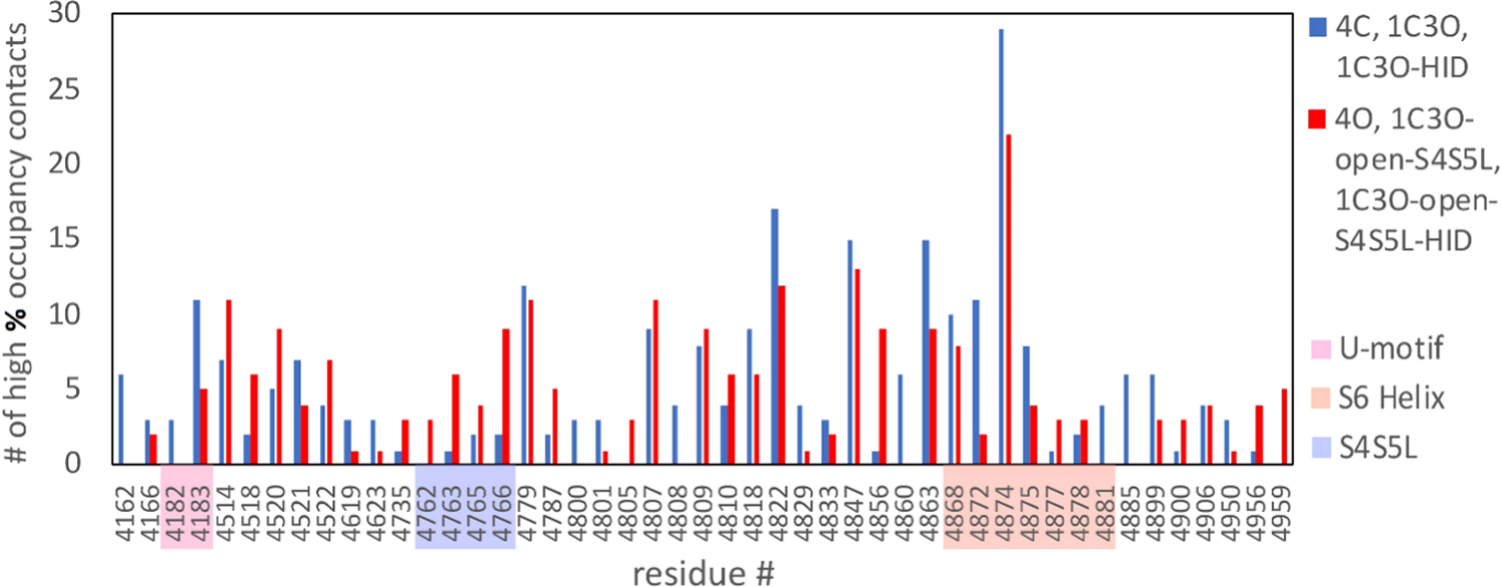
Number of inter-subunit high percent occupancy interactions observed in the closed and open RyR2 systems. The number of inter-subunit high percent occupancy interactions (>30% occupancy) listed by residue number that were observed within our three closed (4C, 1C3O, and 1C3O-HID) and three open (4O, 1C3O-open-S4S5L, and 1C3O-open-S4S5L-HID) RyR2 systems is given. To limit the number of interactions along the *x*-axis for this plot, an additional restriction was made where only residue locations where either the closed or open systems had an interaction appear at least three times were included in this plot. In producing this figure, we pooled data from all four subunits in the three closed RyR2 systems (4C, 1C3O, and 1C3O-HID) and compared it to the pooled data from all four subunits in three open RyR2 systems (4O, 1C3O-open-S4S5L, and 1C3O-open-S4S5L-HID). Residue numbers for regions within the U-motif, S4S5L, and the S6 helix that we focus on in our discussion are highlighted along the *x*-axis. The full set of high percent occupancy interaction data is available in Tables S3 and S4.

Given the observation that there were no high percent occupancy inter-subunit interactions for the 4C system between the S4S5L and its protein environment (Table S1), whereas several inter-subunit interactions appeared between the S4S5L and the S6 helix in the 4O system (Table S1), we decided to examine the high percent occupancy interactions between any residue in a given subunit and its surrounding protein environment at the subunit–subunit interface, ignoring any intra-subunit interactions that occurred within the subunit itself. As with Figure 5, in Figure 7, we pooled the high percent occupancy interaction data together from all four subunits for the three closed RyR2 systems (4C, 1C3O, and 1C3O-HID) and compared it to the pooled data from all four subunits for three open RyR2 systems (4O, 1C3O-open-S4S5L, and 1C3O-open-S4S5L-HID). A similar analysis for the two mutant systems (1C3O-S4S5L-H4762P and 1C3O-open-S4S5L-H4762P) is provided in Figure S13. To fit the high percent occupancy interactions within the limited space on the *x*-axis in Figure 7, we also imposed an additional condition where only residues that had at least three high percent occupancy interactions in either the closed or open systems were included in this plot. To examine variations between the interactions within different subunits in different systems, the full set of high percent occupancy interactions at the subunit–subunit interface is provided in Tables S3 and S4.

Comparing Figure 5 with Figure 7, we see in Figure 7 that there is a noticeable increase in inter-subunit interactions involving residues 4762–4766 within the S4S5L in the open-state systems in comparison to the closed-state systems. This was very similar to the trend seen in Figure 5A, where the open-state systems had a larger number of contacts for most residues in the 4762–4766 sequence compared to the closed-state systems. The peak heights in the two figures are different because Figure 5A reported on both intra-subunit and inter-subunit high percent occupancy contacts, whereas Figure 7 showed only inter-subunit high percent occupancy contacts. The good agreement between the two suggested that in the transition from the closed to the open state, new contacts were formed within the 4762–4766 sequence of the S4S5L and its surrounding protein environment (Figure 5A), and many of these interactions occurred between the S4S5L on one subunit and residues on an adjacent subunit (Figure 7).

Figures 5 and 7 also featured many prominent peaks that appeared on the S6 helix (within residues 4860–4890). However, whereas Figure 5B showed a noticeable increase in the number of high percent occupancy interactions between the S4S5L and the S6 helix (residues 4860–4890) in the open-state systems in comparison to the closed-state systems, Figure 7 shows a noticeable net decrease in inter-subunit high percent occupancy interactions in the S6 helix (residues 4860–4890) for the open-state systems in comparison to the closed-state systems. This implied that when new S4S5L contacts were formed with the S6 helix (residues 4860–4890) in the open-state systems, many inter-subunit contacts in the closed-state systems that involved the S6 helix (residues 4860–4890) were disrupted, leading to a net decrease in inter-subunit interactions for the S6 helix region in the open-state systems when compared to the closed-state systems.

## Discussion

### S4S5L Plays a Critical Role in RyR2 Channel Gating

Using chimera structures that contain elements of the currently available closed and open RyR2 cryo-EM structures, we have tested the effect of making changes to certain structural elements within a single subunit of the RyR2 tetramer on the state of the channel as a whole. In our 1C3O model, the replacement of an open-state subunit with a single closed-state subunit was able to induce the other three open-state subunits to form a closed channel structure (Figures 3 and 4). This suggests a high degree of cooperativity between subunits in channel closing. Our models further predict that changes in the conformation of the S4S5L in a single subunit are critical for determining if the channel is open or closed.

Previous groups have suggested that the S4S5L plays a prominent role in ryanodine receptor channel gating. Ramachandran et al. proposed that the S4S5L directly controls channel gating in the skeletal muscle ryanodine receptor type 1 (RyR1) channel.^28^ It had been well documented that the S4S5L was critical to channel gating for voltage-gated potassium (K^+^) channels,^63−67^ and the authors suggested that RyR channels shared a conserved ion channel gating mechanism with both the sodium (Na^+^) and K^+^ channels.^28^ However, at that time, the highest-resolution cryo-EM RyR1 structure was limited to ∼10 Å, which was too low to observe the fine details of the channel pore. Our MD simulations directly support this notion that the S4S5L is a critical component in RyR2 channel gating, and the use of higher-resolution cryo-EM structures allows us to further examine the key structural elements involved in the gating process.

### Residues 4758–4766 in the S4S5L Alter Binding to the S6 Helix in Channel Gating

Figures 5–7 show that there is a general shift in the binding within the S4S5L (residues 4758–4766) toward the central S6 helix in the open state versus the closed state. In the closed state, residues on the S4S5L generally bind to the S6 helix within residues 4865–4869 on the same subunit, but residues 4758–4766 in the S4S5L increasingly bind to the S6 helix (residues 4870–4880) on an adjacent subunit in the open state. In the process where the S4S5L forms new contacts with the S6 helix in the open state, some of the inter-subunit contacts in the S6 helix (residues 4870−4880) that were present in the closed state are broken, opening the channel. The evidence for this is that there is a net loss of contacts in the 4870–4880 region of the S6 helix in the open state in comparison to the closed state (Figure 7). The importance of S6 helix residues near and within this sequence (D4868, E4872, R4874, and E4878) to channel gating of RyR2 was established experimentally in mutation studies by Peng et al.^32^

The use of MD simulations has allowed us to identify the most important residue–residue interactions involved between the S4S5L and the S6 helix in the closed and open RyR2 systems (Tables S1 and S2). The most prominent of these were interactions involving residues N4763 or Q4766 and residues D4868 and N4874 on the S6 helix (Figure 5). The N4763–D4868 interaction most commonly appeared as an intra-subunit contact between the S4S5L and the S6 helix (Figure 6A), and this interaction was frequently present in both the open and closed systems (Tables S1 and S2). Given that the D4868 residue is near the hydrophobic gate (I4867) and gating hinge (G4864) residues, it seems feasible that this interaction is the primary interaction coupling changes in the conformation of the S4S5L to the hydrophobic gate and the gating hinge.

In contrast, in the open-state systems, the N4763–R4874 (Figure 6B) or Q4766–R4874 interactions most commonly appear as inter-subunit interactions between the S4S5L on one subunit and the S6 helix on a second subunit (Figure 5). It appears that a shift in the position of the S4S5L relative to the S6 helix, which allows N4763 or Q4766 to bind to R4874 on an adjacent subunit, is a primary mechanism that distinguishes the open state from the closed state (Tables S1 and S2). This interaction between adjacent subunits is likely crucial for cooperative channel closing, as was observed with the 1C3O system.

### N4763 Can Act as a Switch to Control RyR2 Channel Gating

**Figure 8:**
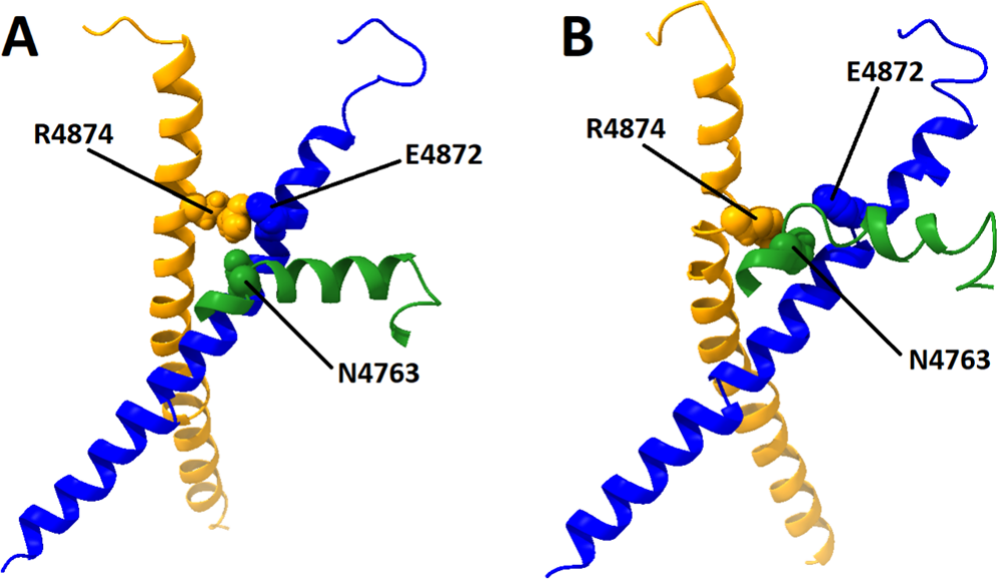
Residue N4763 on the S4S5L can act as a switch that controls RyR2 channel gating. N4763 (green spheres) within the S4S5L (residues 4746–4766, green ribbons) in subunit 1 is normally out of position in the closed 4C system in panel (A) to interfere with an inter-subunit interaction between E4872 (blue spheres) on the S6 helix in subunit 1 (residues 4839–4889, blue ribbons) and R4874 (orange spheres) on the S6 helix in subunit 2 (residues 4839–4889, orange ribbons). In the open 4O system in panel (B), a shift in the position of the S4S5L allows N4763 to interact with R4874, replacing the previous inter-subunit interaction between R4874 and E4872. Both structures for 4C and 4O represent an average structure over the last 100 ns of the 1 μs MD simulation. This image was produced using UCSF Chimera X 1.4.

It had been previously suggested by Peng et al.,^32^ based on a structural comparison of the closed and open RyR2 systems, that N4763 plays a key role in RyR2 channel gating. In Figure 8, we illustrate a plausible mechanism for how N4763 can act as a switch to control channel gating. In the closed 4C system (Figure 8A), N4763 (green spheres) within the S4S5L (residues 4746–4766, green ribbons) in subunit 1 is normally out of position to interfere with an inter-subunit interaction between E4872 (blue spheres) on the S6 helix in subunit 1 (residues 4839–4889, blue ribbons) and R4874 (orange spheres) on the S6 helix in subunit 2 (residues 4839–4889, orange ribbons). In all three of our closed-state systems (4C, 1C3O, and 1C3O-HID), this inter-subunit E4872–R4874 interaction is observed as a high percent occupancy interaction between residues on subunits 1 and 2 (Tables S3 and S4).

In the open 4O system in Figure 8B, a shift in the conformation and position of the S4S5L helix allows N4763 in subunit 1 to interact with R4874 in subunit 2, abolishing the previous inter-subunit interaction between E4872 and R4874. As a result, the distance between residues E4872 and R4874 on adjacent S6 helices at the cytosolic end of the channel is noticeably increased, an effect associated with channel opening. In all three nonmutant open-state systems, the N4763–R4874 interaction appears much more frequently at the subunit 1–2 interface than the E4872–R4874 interaction (Tables S3 and S4). The situation for the H4762P mutant systems is more complicated as neither the E4872–R4874 interaction nor the N4763–R4874 interaction appear as frequently at the inter-subunit interface. In place of N4763–R4874, the V4768–R4874 interaction is much more commonly observed. This is perhaps not too surprising given that the location of the H4762P mutant is directly adjacent to the N4763 residue on the S4S5L, and this may have had a direct impact on its ability to bind to R4874.

### Residues 4751 and 4754 Link the S4S5L to the U-Motif

In addition to residues 4758–4766 in the S4S5L binding to the S6 helix, the S4S5L has a noticeable number of high percent occupancy interactions between residues 4748–4755 (Figure 5A) and residues 4167–4184 in the U-motif (Figure 5B). The most prominent interactions that were seen within this region involved residues K4751 and R4754 within the S4S5L. The importance of K4751 in RyR2 channel gating was recently demonstrated by Uehara et al., who showed that the single point mutation K4751Q was associated with extensive RyR2 channel leak.^68^ Two examples of interactions are the K4751–E1467 and R4754–D4175 interactions, which are both visible in Figure 6B. While high percent occupancy interactions within the U-motif at position D4175 were common in both the closed and open-state systems, high percent occupancy interactions involving residue E1467 were only observed in the open-state systems. In fact, a greater number of high percent occupancy interactions were observed throughout the U-motif in the open state as opposed to the closed state in general (Figure 5B). These data suggest that the U-motif may play a role in shifting the S4S5L from its position in the closed state to the altered position of the S4S5L that is observed in the open state.

**Figure 9:**
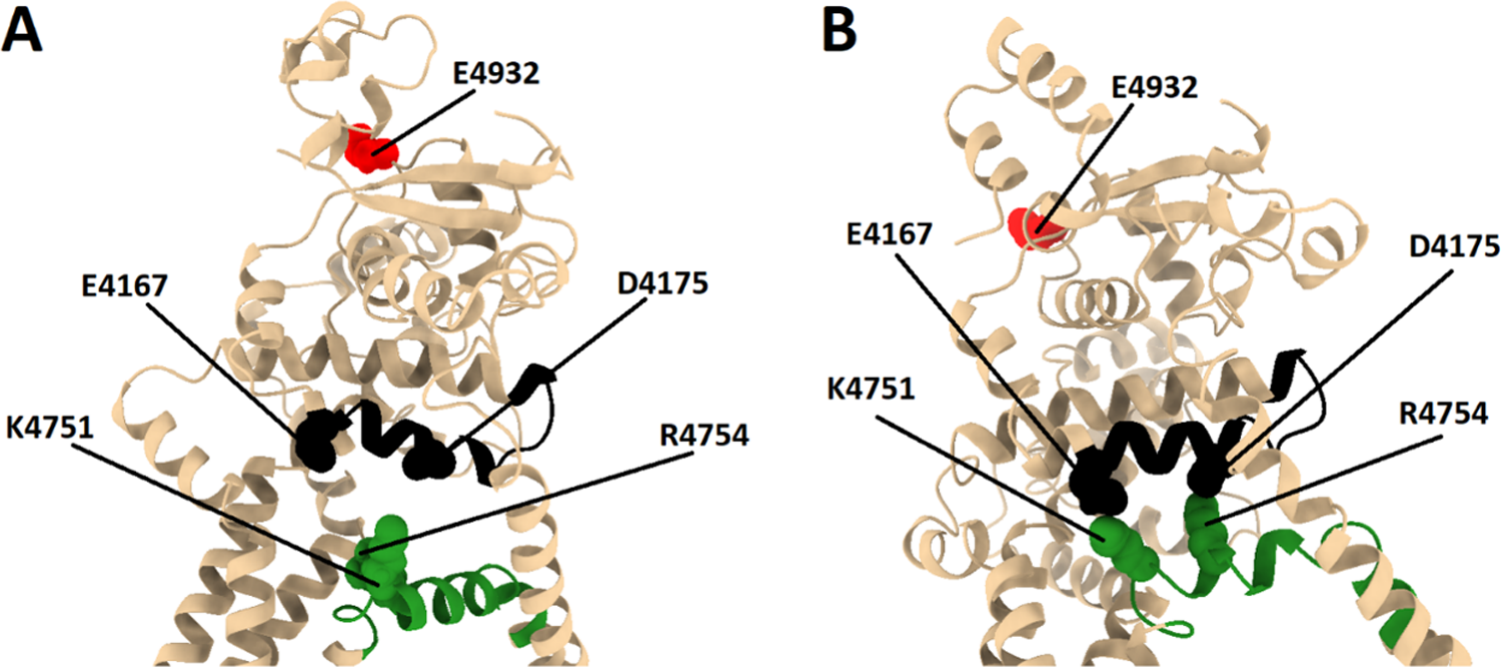
Key high percent occupancy interactions between the S4S5L and the U-motif. A closeup of the key high percent occupancy interacting residues (solid spheres) between the S4S5L (residues 4746–4766, green) and the U-motif (residues 4167–4184, black) is provided for the closed 4C system in panel (A) and the open 4O system in panel (B). Residue E4932, which is one of a trio of residues that coordinate to Ca^2+^ at the calcium activation site, is shown with red spheres. Both structures for 4C and 4O represent an average structure over the last 100 ns of the 1 μs MD simulation. This image was produced using UCSF Chimera X 1.4.

Indeed, an adjacent domain above the U-motif contains one of the three Ca^2+^ binding residues (E4932) that are believed to form the Ca^2+^ activation site in the RyR2. This observation suggests an extended mechanism in which the binding of Ca^2+^ to residue E4932 can shift the position of the nearby U-motif to interact with the S4S5L at positions K4751 and R4754. This interaction would then move the S4S5L upward toward the U-motif, altering its interactions with the S6 helix. In turn, the altered S4S5L–S6 helix interactions would lead to the breaking of inter-subunit interactions in the closed-state S6 helix, opening the channel. The removal of Ca^2+^ could then shift the U-motif away from the S4S5L, reversing the mechanism and restoring the closed state. A closeup of the S4S5L and U-motif in both the closed and open states highlighting these residues is provided in Figure 9.

We note that a mechanism for RyR2 channel opening was also suggested by Peng et al.^32^ in which a portion of the central domain including the U-motif interacts directly with the S6 helix to open the RyR2 channel. We must make it clear that our results do not rule out this possibility. Our results indicate that the position and conformation of the S4S5L is indeed critical to the mechanism of RyR2 channel gating as previously suggested by Ramachandran et al. for the RyR1 channel,^28^ and we also observe many high percent occupancy interactions between the U-motif and the S4S5L. However, it must be kept in mind that the full physiological allosteric mechanism will involve several other structural changes in nearby structural elements such as the S6 helix and other regulatory domains within the RyR2 that can affect channel gating as well.

In addition, we should point out that we observe structural disorder in the central domain of the channel pore profiles between −40 and −60 Å (Figure 3), when we compare 1C3O and 1C3O-HID for instance, that had no observable effect on the overall state of the channel. We also did not see a clear correlation in our RMSD analysis in Table 2 between the central domain and the state of the channel. These results are seemingly in contradiction to our key interaction analysis (Figures 5 and 7), which shows a clear correlation to the state of the channel involving certain key interactions within the central domain that link the U-motif to the S4S5L. However, it is entirely possible that the flexibility observed in the central domain as a whole is independent of the well-defined interactions that are taking place between the U-motif and the S4S5L locally. We note that allosteric regulatory regions on proteins are typically disordered, and yet, they are involved in a predictable change of state despite the high degree of disorder observed in the regulatory region. This can occur because the change in state is brought about by a small local change within the disordered region, as when binding to a small regulatory molecule, for instance. In this case, we should consider that the residues in the U-motif that interact with the S4S5L comprise a very small portion of the rather large central domain that is being observed in Figure 3 and the RMSD analysis.

Another possibility is that we may have altered distant allosteric mechanisms using a structure that is not complete enough in the region surrounding the central domain, or the computational method we are using to induce the channel to close may be overriding the effects of allostery in this system. Therefore, a definitive role for the U-motif is not clearly established within the scope of our present study. Further computational studies using larger, more complete systems, where additional conformational changes are made directly to the U-motif and to many other nearby structural elements such as the S6 helix, must be carried out before the full allosteric mechanism of RyR2 channel gating can be clearly established using this methodology. Nevertheless, our current models reveal that there are key high percent occupancy interactions between the U-motif and the S4S5L, and this suggests that a direct coupling between these two domains may play an important role in the gating mechanism.

### Cooperative Effects Induce the S4S5L at Three Inter-Subunit Interfaces to Adopt a Closed Conformation in the 1C3O Chimera System

In the previous section, we proposed a general mechanism for RyR2 channel gating (Figures 8 and 9) that was based on the identification of key high percent occupancy interactions (Figures 5 and 7) that best differentiated our three closed-state systems (4C, 1C3O, and 1C3O-HID) from our three open-state systems (4O, 1C3O-open-S4S5L, 1C3O-open-S4S5L-HID). One question that has yet to be addressed is the degree of cooperativity between the conformations of the S4S5L on different subunits in channel gating. As our pore profile results indicated (Figure 3), the substitution of only one subunit from the 4C system, with the S4S5L initially in a closed conformation, into the open 4O system, with three subunits initially in an open conformation, was able to close the entire channel in both the 1C3O and 1C3O-HID model systems. That a single subunit in a closed conformation can induce the entire four-subunit system to close suggests that channel gating in the RyR2 is highly cooperative.

What has not been addressed to this point is the degree to which the conformation of the S4S5L in the other three open-state subunits changed after the subunit containing an S4S5L in a closed conformation was inserted into the open-state system in our chimera models, 1C3O and 1C3O-HID. There are two extreme cases to consider. The first possibility is that the transition to the closed state observed in Figure 8 occurs at all four inter-subunit interfaces, and not just at the inter-subunit interface where the initial substitution took place. This implies that when we examine each inter-subunit interface in our chimera systems (1C3O and 1C3O-HID) at the end of our MD simulations, the key high percent occupancy interactions in the S4S5L that we observe at each interface should resemble the closed-state 4C system by the end of the MD simulation. The second possibility is that the transition to the closed state observed in Figure 8 only occurs at one inter-subunit interface, at the location where the initial mismatch occurred. This implies that when we examine each inter-subunit interface in our chimera systems, the change in the key high percent occupancy interactions in the S4S5L that we observe should appear only at the interface where the substitution took place, while the interactions at the other three inter-subunit interfaces should still resemble those of the open-state system.

We can examine the predictions made in these two scenarios using the key interactions identified in our high percent occupancy data. Pooling our data together in Figures 5 and 7 makes an analysis of what is taking place at each inter-subunit interface difficult, but this analysis can be made more explicit by examining the raw data in Tables S1–S4, which provides a full listing of each high percent occupancy interaction for every subunit. As an explicit example, we focus on the high percent occupancy interactions involving residue R4874, which can be seen in Figure 5 to be the most prominent residue that distinguishes the closed state from the open state. In particular, we focus on interaction N4763–R4874, which was highlighted as a representative key interaction in the proposed mechanism shown in Figure 8. As can be verified using Tables S1 and S2, the N4763–R4874 interaction appeared only once across 12 subunits in the three closed 4C, 1C3O, and 1C3O-HID systems. Other interactions involving residue R4874, or possibly involving nearby residues L4873 or D4875, were not observed at all in the closed-state systems. This observation is in agreement with the representative conformation of the closed state we show in Figure 8A where an interaction between residues N4763 and R4874 is not observed.

In contrast, the N4763–R4874 interaction appears six times in the three open-state systems 4O, 1C3O-open-S4S5L, and 1C3O-open-S4S5L-HID, while close structural variants such as Q4766–R4874 and H4762–R4874 appeared three and two times, respectively. Altogether, this makes 11 very similar interactions involving residue R4874 that were observed across 12 subunits in the three open state 4O, 1C3O-open-S4S5L, and 1C3O-open-S4S5L-HID systems. In the case of 1C3O-open-S4S5L and 1C3O-open-S4S5L-HID systems, other similar variant interactions that involved nearby residues L4873 and D4875 were also observed in addition to R4874. These observations are in good agreement with the presence of the N4763–R4874 interaction in Figure 8B, which was used as a representative key interaction for the open state.

Hence, our data would seem to suggest that the first possibility outlined in the discussion above better describes the cooperative channel closing we observe in the RyR2. If the second possibility was taking place, we would expect up to six of our subunits in the 1C3O and 1C3O-HID, which were initially in the open-state conformation, to frequently display either the N4763–R4874 interaction or at least to display other close structural variants involving residue R4874 or other nearby residues. As can be verified in Tables S1 and S2, only one of the six subunits in the 1C3O and 1C3O-HID systems still retains an interaction with R4874, and this happens to be a N4763–R4874 interaction. Nevertheless, the presence of this one interaction at one inter-subunit interface in a closed system indicates that an all-or-nothing cooperativity mechanism that requires all four S4S5L to adopt a closed-state conformation might be too restrictive. Our key interaction data for R4874 suggests that channel closing may still be possible when three subunits are in the closed S4S5L conformation, while the S4S5L at one interface remains in the open-state conformation. However, due to the presence of so many structural variants in the vicinity of R4874 that may play compensating structural roles (Tables S1 and S2), it is difficult to say with certainty if this is the case based on an analysis of our high percent occupancy interactions alone.

**Figure 10:**
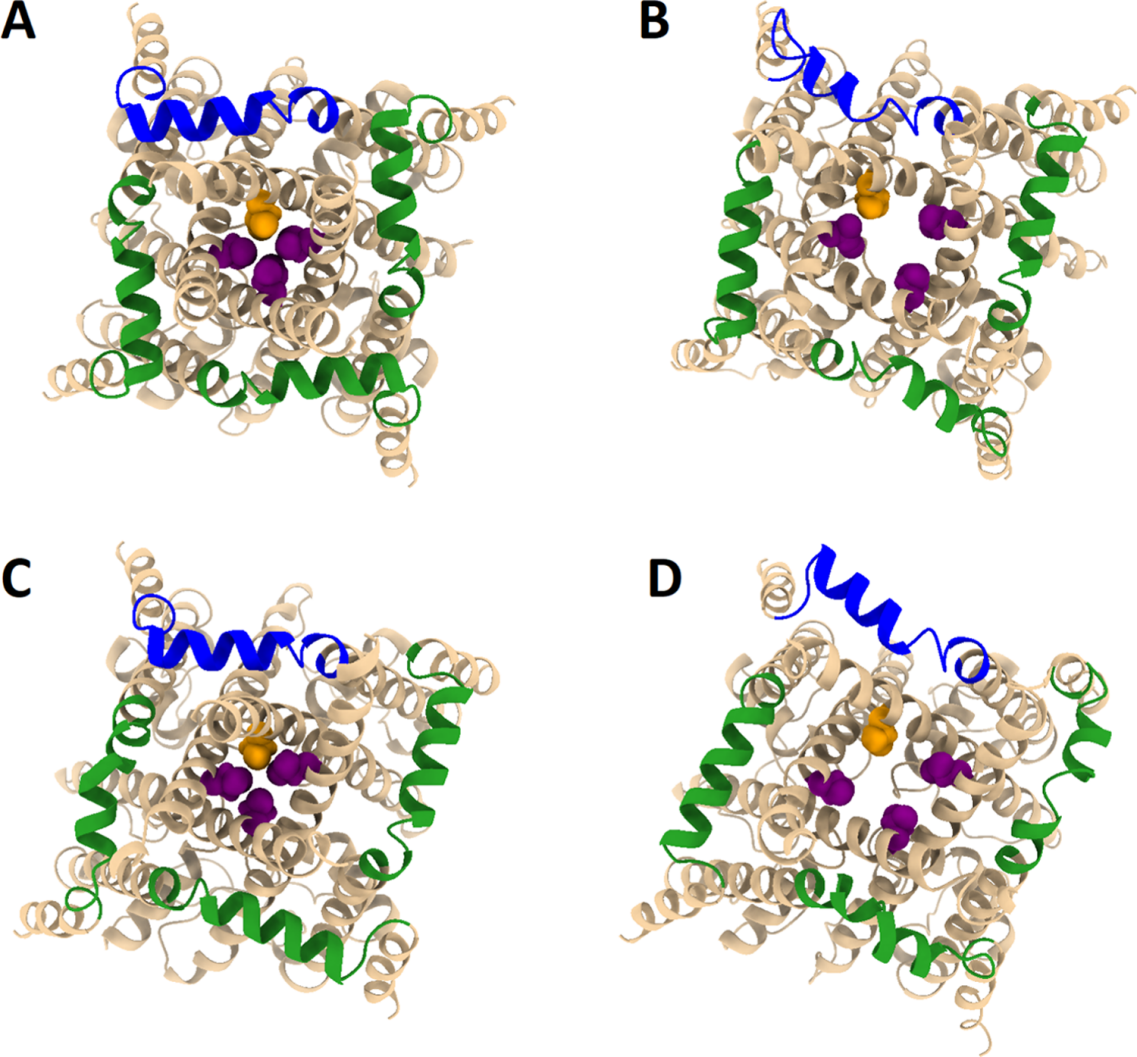
Conformation of the four-subunit S4S5L structure in the 1C3O chimera RyR2 system indicates cooperative channel closing. The structure in the vicinity of the hydrophobic gate in the transmembrane domain of the RyR2 is shown for the closed 4C system (A), the open 4O system (B), the closed 1C3O chimera system (C), and the 1C3O-open-S4S5L chimera system (D). The isoleucine residue that forms the hydrophobic center of the channel pore (I4867) is depicted using orange VDW spheres for the first subunit and purple VDW spheres for the other three subunits. The S4S5L (residues 4746–4766) is colored blue in the first subunit of each system while the S4S5L on the other three subunits is colored green. All images represent an average structure over the last 100 ns of the 1 μs MD simulation. This image was produced using UCSF Chimera X 1.4.

A visual examination of the global four-subunit S4S5L conformations in our final MD systems is provided in Figure 10. Figure 10 qualitatively supports the suggestion that S4S5L conformational changes at only three out of the four inter-subunit interfaces may be sufficient to close the channel. We see that the overall conformation of the four-subunit S4S5L in the 1C3O chimera system in Figure 10C is somewhere between the conformation of the four-subunit S4S5L closed 4C system in Figure 10A and the conformation of the four-subunit S4S5L in the two open-state systems, 4O and 1C3O-open-S4S5L in Figure 10B,D, respectively. A careful examination of the four-subunit S4S5L structure in the 1C3O system in Figure 10C reveals that three out of the four S4S5L have formed a tighter arrangement at the corners, which most closely resembles the very tightly packed 4C system in Figure 10A. However, it can also be seen that one of the four S4S5L linkers in Figure 10C is more similar to an open-state conformation, which suggests that a full transition to the closed-state conformation of the four-subunit S4S5L configuration in 1C3O may not be complete within the 1 μs MD simulation.

Nevertheless, we note that this change in 1C3O was able to close the channel as indicated by a collapse of the central four isoleucine residues that form the hydrophobic gate at the center of the channel pore (Figure 10C). This close arrangement of the central isoleucine residues in 1C3O is clearly comparable to the arrangement of the four central isoleucine residues in the closed 4C system (Figure 10A). In contrast, the four central isoleucine residues remain in an open configuration in the 1C3O-open-S4S5L system (Figure 10D), which closely resembles the open 4O system (Figure 10B). Since the only initial difference between the two chimera systems was that 1C3O started with the S4S5L on subunit 1 (blue) in the closed conformation while 1C3O-open-S4S5L started with the S4S5L on subunit 1 (blue) in the open conformation, we see that the closed conformation of the S4S5L in only one subunit can affect the packing of at least three of the S4S5Ls around the central hydrophobic gate and that this conformational change in the four-subunit S4S5L configuration is capable of closing the central hydrophobic gate.

Figure 10 also suggests a qualitative explanation for how the insertion of one subunit can lead to the collapse of an entire four-subunit S4S5L structure based on the observed symmetry of the structures. The four-subunit S4S5L structures from the wild type cryo-EM structures both adopt regular symmetrical shapes, with the closed 4C system displaying a square shape in Figure 10A, and the open 4O system displaying a rhombus shape in Figure 10B. This difference in conformation between the two S4S5L shapes can be traced back to the conformational change in the S4S5L between the closed and open states depicted in Figure 2. In Figure 10A,B, this conformational change in the S4S5L appears in all four subunits simultaneously. When a subunit with the S4S5L in a closed conformation is inserted into the open state, as in 1C3O, the symmetry of the rhombus is broken, and the overall four-subunit structure collapses, which closes the channel pore, as observed in Figure 10C. This collapse does not occur in 1C3O-open-S4S5L. When the closed-state subunit was inserted in 1C3O-open-S4S5L, it still contained the S4S5L in an open conformation, so the rhombus shape needed to maintain the open state was still preserved at the end of our MD simulations, as seen in Figure 10D.

Overall, our results suggest that channel closing of the RyR2 is cooperative, and substitution of only one subunit with the S4S5L in a closed-state conformation into an open-state system induces the S4S5L in three out of the four subunits to adopt a closed conformation. Figure 10 qualitatively suggests that this cooperativity ultimately emerges from the need to maintain a rhombus shape in all four subunits in the S4S5L to keep the central four isoleucine residues from collapsing inward and closing the channel. However, we note that this latter observation is qualitative, and a further study that explores the geometry of the four-subunit configuration of the S4S5L in more detail is needed. We also note that our present study of cooperativity leaves several additional open questions that must be examined more thoroughly in further studies. Although our data suggest that three inter-subunit interfaces underwent a transition from the open to closed state, the minimum number of inter-subunit interfaces that must undergo a transition from an open-state conformation to a closed-state conformation to effectively close the channel still remains an open question. We have addressed this topic further using a different computational approach in a separate study [manuscript under review at the time of this publication]. In addition, the time course of the cooperative S4S5L inter-subunit transitions still remains to be addressed. For example, we do not know from our end-state analysis in Figure 10 if the conformational changes in the S4S5L at the various inter-subunit interfaces are occurring sequentially or if the relevant changes occurred simultaneously, or somewhere in between these two extremes. As pointed out previously by Nury et al.,^47^ the time course of channel closing occurs over two very different timescales, with a rapid initial decrease in the size of the minimum pore radius followed by very slow adjustments to the minimum pore radius that occur over the course of the entire 1 μs MD simulation (Figures S3–S6). The initial rapid partial pore closing, and the subsequent slow adjustments to the pore that occur over the entirety of the MD simulation, make it very difficult to clearly differentiate between these two possibilities using our present methodological approach. A closer examination of the time course of channel gating using more advanced computational techniques such as time–structure independent component analysis or tICA^69^ would be an attractive avenue for a future study.

### Inter-Subunit High Percent Occupancy Interactions Correlate with CPVT/LQTS Hotspots

One of the mysteries about RyR2 channel function involves the observation that many different single amino acid substitutions are capable of causing CPVT and/or LQTS symptoms. A listing of 63 possible CPVT1-associated RyR2 mutations was provided by Medeiros-Domingo et al.^6^ Of these, 49 fall within the residue sequences included in the eight RyR2 models in our present study (Figure S2 and Table S5). In our 1C3O-H4762P model system, we found that a single point mutation in a single subunit could prevent the channel from closing. The H4762 residue was identified in our high percent occupancy interaction analysis for both the S4S5L bound to the surrounding protein environment (Figure 5) and in our inter-subunit interaction analysis (Figure 7).

Additionally, we note that the H4762 residue appears in sequence next to another high percent occupancy interaction site, N4763. A comparison of the CPVT1 mutations listed by Medeiros-Domingo et al.^6^ shows that this is not a unique situation. Out of the 49 mutant residue locations that appear in our models, 73% (36) of them were located within five residues of a residue listed as a high percent occupancy interaction in our inter-subunit interaction analysis of the three closed-state systems (4C, 1C3O, and 1C3O-HID), while 65% (32) of them fall within three residues of a residue listed as a high percent occupancy interaction (Table S5). The correlation between high percent occupancy interactions and mutation hotspots may even be stronger than these numbers suggest, as of the 27% of residues that fall outside this range, many are close in sequence to regions of our models that are missing residues in comparison to the full-size RyR2 (Table S5). Hence, overall, there appears to be a strong predictive correlation between inter-subunit high percent occupancy interaction sites identified in our MD simulations and nearby mutations that lead to CPVT or LQTS.

In previous sections, we have described a mechanism for RyR2 channel opening that involved the breaking of inter-subunit high percent occupancy interactions in the closed state. This appears to be carried out in a controlled way in the wild type; the position of residue N4763 on the S4S5L is shifted such that it can bind to R4874, which then breaks the inter-subunit interaction to E4872 associated with the closed state (Figures 5, 7, and 8). The strong correlation between inter-subunit high percent occupancy interactions and known CPVT and LQTS mutations suggests that, in general, disruption of inter-subunit high percent occupancy interactions may be a common mechanism of many point mutations that lead to channel leak and CPVT in the RyR2 system. This notion is consistent not only with the mutations listed in the study by Medeiros-Domingo et al.^6^ but is also consistent with the disruption of high percent occupancy inter-subunit interactions at positions 4902, 4950, and 4955 that was recently reported in association with defective channel closing by Guo et al.,^70^ it is consistent with the report of massive channel leak at position 4751 by Uehara et al.,^68^ and it is consistent with the results of mutation studies for residues D4868, E4872, R4874, and E4878 reported by Peng et al.^32^ These results collectively suggest that maintaining high percent occupancy interactions between adjacent subunits at key locations within the RyR2 closed-state system is crucial to preventing channel leak and CPVT or LQTS.

### Study Limitations, Advances, and Future Directions

The computational methods we used in the present study were directly based on a protocol developed by Nury et al.^47^ where the authors used a 1 μs, single-trajectory MD simulation to study the gating properties of the nicotinic receptor homologue. The authors were able to show that an instantaneous change in pH can be introduced to the open state of the channel to induce the channel to close, and it was shown that there was a quick adjustment to the pore radius that effectively closed the channel within 50 ns. This was followed by a twist of the channel for 450 ns and a slow relaxation of the channel up to 1 μs. The authors experimentally validated their protocol by comparing their end-state model to the known structure of the closed-state channel.^47^

Our present study confirms that the 1 μs, single-trajectory protocol developed by Nury et al. can be used to induce gating transitions from the open to the closed state of a large membrane protein system. In our study of the RyR2 receptor, we observed a very similar short timescale closure of the channel within 50–400 ns followed by a slower relaxation period for 500 ns. This was then followed by equilibration of the channel for ∼100 ns (Figures S3–S6). We calculated the key interactions involved in channel gating from our end-state models, and we validated our model by comparing our calculated key interactions with those previously identified in the experimental literature.

Nevertheless, we note that there are many limitations in our present methodology that can be improved upon in future studies. In particular, one of the major limitations of using single-trajectory MD simulations is that they do not provide a way to assess the variation or error in the results from our MD trajectories. Changing from a single-trajectory approach to using a multitrajectory approach, where several replica systems are produced for each individual system, would directly address this issue.

The downside of using a multitrajectory approach is the greatly increased computational cost. For studies where the overall system size is small, the total simulation time is relatively short, and when the number of systems being studied in small, this increase in computational cost might also be small, making the multitrajectory approach a better alternative to using the single-trajectory approach. For this study, the overall system size is very large, we have a long 1 μs total simulation run time, and eight different unique systems are analyzed. All of these factors come with a high computational cost, which is a roadblock to transitioning the method directly into a multitrajectory study.

Modifications to our method can be made in future studies to help alleviate some of this additional cost. For example, according to our RMSD data, total simulation times can be lowered well below the 1 μs run time used by Nury et al., as we show that most of our systems started to equilibrate at about 600 ns (Figures S3–S6). In addition, the number of systems that are analyzed can be lowered once the effect of the S4S5L substitution, for instance, is established by single-trajectory runs. Advances in technology may also make the multitrajectory approach more viable for large systems like this in the future. Taking this into account, a multitrajectory approach to assess variation within each system should become an attractive avenue for a future study of this system.

There are other areas of the method that can also be improved albeit also with an additional computational cost. The introduction of instantaneous structural changes to induce the channel to close leads to an abrupt, direct, rapid closing of the channel within 50–400 ns. This rapid transition may not be truly representative of the conformational ensemble of closing pathways available in the natural state of the channel protein. Other more sophisticated methods such as targeted MD may provide for a more gradual transition of channel closing.^71^

As with the study by Nury et al.,^47^ we focus on the closing of the open state of the RyR2 channel. The inverse process, the opening of the closed-state channel would pose a greater challenge to study using our present approach. The central channel pore of the closed-state 4C channel is much more compact overall than the open-state 4O structure (Figure S7). This leaves less room for introducing structural changes to the 4C system as compared with introducing changes into the 4O system. Substituting in smaller portions of the 4O subunit into the 4C system, as opposed to the entire subunit, may be one way to address this issue. A comprehensive understanding of channel gating will eventually require the study of both closing and opening processes, especially in situations where the mechanism for channel gating is not reversible.

In this study, we followed the established single-trajectory MD protocol, which included providing a global RMSD analysis of each structure compared to its initial structure file over the 1 μs timeframe of the MD trajectory (Figures S3 and S4). All structures exhibited a shift of 5–7 Å from their initial structure file after our structures had started to equilibrate by around 600 ns. Although we cannot guarantee that additional changes in the structures will not occur if the simulations were run longer than 1 μs, we can say that no major transitions were observed over the last 400 ns of the 1 μs MD simulations in any of our eight different systems.

The final RMSD values between 5 and 8 Å at equilibrium do indicate that noticeable changes take place between the initial and final structures during the course of the MD simulation in all cases. We note that global RMSD values for the final average systems in Table 2 are also about the same size (5–8 Å) when compared to the final average 4C structure. We additionally observe in Table 2 that RMSD values tend to be much smaller and correlate more closely with the final state of the channel, when we examine smaller substructural elements of the channel, such as the S4S5L or the S6 helix, instead of larger substructures of the models. The data in Table 2 suggests that the magnitude of RMSD values might depend on the size of the structures involved in a given comparison, but a further study would be required to fully establish this notion. Our RMSD analysis can also be expanded in a future study to include comparisons of individual subunits or substructural elements involved in cooperative channel opening.

For our residue–residue interaction analysis, we pooled our data together from all four subunits for all three closed systems (4C, 1C3O, and 1C3O-HID) and for three open systems (4O, 1C3O-open-S4S5L, and 1C3O-open-S4S5L-HID) in our analysis in Figures 5 and 7. We did it this way because pooling our data was the best solution we could find to combine data from 24 different subunits and six different models into a single figure that would fit on the printed page. However, the downside to this approach is that each system also has clear variational differences within each subunit and between models that make each system unique in many ways. While we do not provide this information in Figures 5 or 7, we have provided the full data set that was used to construct Figures 5 and 7 in Tables S1–S4. A detailed understanding of all subunit–subunit interactions would have to take into account not only the locations of key interactions but also how time-dependent changes in the interaction patterns at the individual subunit level can affect the cooperative transition from the open to closed state. This is a long-term goal that should be a central focus in future studies.

We note that the use of MD simulations provided us with a way to examine the role of the S4S5L in channel gating by allowing us to make changes to the original structure files. It also allowed us to identify and rank key interactions in a quantitative manner using our high percent occupancy interaction data as opposed to using a qualitative visual analysis from a static PDB structure. This has an advantage in that our structural studies and high percent occupancy interactions do not rely on human intuition to make predictions, potentially making them more accurate and comprehensive than a traditional analysis based on a visual inspection of the structure. Once a key structural element or a high percent occupancy interaction is identified, we can return to a visual inspection of the structure to confirm the biophysical validity of the result. However, one drawback is that the MD approach is obviously much more expensive and labor intensive than a visual analysis. It should also be considered that the need to adopt an artificial method to induce the channel to close within a 1 μs timeframe and the incomplete cryo-EM structures can potentially introduce structural artifacts that may affect the gating transition. Improved MD studies that use alternative channel closing strategies and more complete cryo-EM structures would help address these issues, and in all cases, additional experimental studies will be necessary to test any new predictions made with each MD model.

Using our present methodology, we have shown that several of our key interaction results are in agreement with those documented previously in the literature. For example, our most prominent key interaction in the S4S5L was N4763, which was previously identified from a visual inspection by Peng et al.,^32^ while residue K4751 was identified separately by Uehara et al.^68^ The computational approach outlined here provided both key interaction hotspot locations together without the need for a visual analysis. In addition to these, our results also predict novel hotspot locations that have not yet been studied. For example, our results predict that Q4766 in the S4S5L should play a prominent role in the open to closed transition, but at present, we have not found an experiment to substantiate this prediction. This then makes Q4766 a possible mutation target for a future study. Finally, we emphasize that the agreement between the key interactions in our calculated results and in experimental results from several different groups serves to validate our method and gives us confidence that our main results are sound, even if the MD methods that we employed can be expanded and improved upon.

## Conclusions

In this study, we have found, using MD simulations of chimera structures, that the conformation of the S4S5L on a single subunit is critical to controlling the cooperative gating mechanism of the wild-type RyR2 channel. Our models suggest that the mechanism involves establishing new inter-subunit high percent occupancy interactions between the S4S5L on one subunit and key residues along the S6 helix on an adjacent subunit, while breaking key inter-subunit S6 helix interactions that were present in the closed state. Our models also suggest that RyR2 channel closing is highly cooperative, as a change in the conformation of the S4S5L on only one subunit was able to induce the entire four-subunit channel to close. This was despite the other three subunits starting in the open conformation.

Further, when comparing our inter-subunit high percent occupancy interaction analysis with the literature, we found that 73% of mutations identified with CPVT1 occur within close proximity to a high percent occupancy interaction site identified in the closed-state systems in this study. Thus, channel leak associated with CPVT appears to be associated with a general mechanism of inter-subunit interaction disruption at key locations within the subunit–subunit interface of the closed state. The loss of these key inter-subunit interactions allows the subunits to move apart from each other, effectively leading to the same sort of channel opening that is induced in a controlled way when the repositioning of the S4S5L breaks key inter-subunit S6 helix interactions in the wild type. If this proposition is found to be generally true in future CPVT-related studies, it suggests that one possible treatment option would be to design drugs that intercalate between subunits in the RyR2 system, effectively increasing the inter-subunit association back to a level more resembling the wild type. It might even be possible to design a drug that improves subunit–subunit association irrespective of the particular mutation within the channel domain that initially led to a loss of high percent occupancy interactions and CPVT symptoms.

## Supporting Information Available

Peptide bonds between the ends of missing sequences, amino acid sequence in a subunit in the RyR2 model, RMSD plots of model RyR2 systems, minimum radius plots of all model RyR2 systems, side view and top-down view structures of all eight RyR2 model systems, alignment of the S4S5L gating loop for all eight model systems compared to the 4C model, S4S5L and inter-subunit high percent occupancy interactions observed in the H4762P mutant RyR2 systems, and full data set of S4S5L and inter-subunit high percent occupancy interactions (PDF)

## References

[ref1] Meissner G. The Structural Basis of Ryanodine Receptor Ion Channel Function. J. Gen. Physiol. 2017, 149, 1065–1089. 10.1085/jgp.201711878. 29122978 PMC5715910

[ref2] Yamaguchi N. Molecular Insights into Calcium Dependent Regulation of Ryanodine Receptor Calcium Release Channels. Adv. Exp. Med. Biol. 2020, 1131, 321–336. 10.1007/978-3-030-12457-1_13. 31646516

[ref3] Fabiato A. Calcium-Induced Release of Calcium from the Cardiac Sarcoplasmic Reticulum. Am. J. Physiol. – Cell Physiol. 1983, 245, C1–C14. 10.1152/ajpcell.1983.245.1.C1. 6346892

[ref4] Li M. X.; Hwang P. M. Structure and Function of Cardiac Troponin C (TNNC1): Implications for Heart Failure, Cardiomyopathies, and Troponin Modulating Drugs. Gene 2015, 571, 153–166. 10.1016/j.gene.2015.07.074. 26232335 PMC4567495

[ref5] Szentesi P.; Pignier C.; Egger M.; Kranias E. G.; Niggli E. Sarcoplasmic Reticulum Ca2+ Refilling Controls Recovery from Ca2+-Induced Ca2+ Release Refractoriness in Heart Muscle. Circ. Res. 2004, 95, 807–813. 10.1161/01.RES.0000146029.80463.7d. 15388639

[ref6] Medeiros-Domingo A.; Bhuiyan Z. A.; Tester D. J.; Hofman N.; Bikker H.; van Tintelen J. P.; Mannens M. M.; Wilde A. A.; Ackerman M. J. The RYR2-Encoded Ryanodine Receptor/Calcium Release Channel in Patients Diagnosed Previously with Either Catecholaminergic Polymorphic Ventricular Tachycardia or Genotype Negative, Exercise-Induced Long QT Syndrome: A Comprehensive Open Reading Frame Mutational Analysis. J. Am. Coll. Cardiol. 2009, 54, 2065–2074. 10.1016/j.jacc.2009.08.022. 19926015 PMC2880864

[ref7] Fu D. G. Cardiac Arrhythmias: Diagnosis, Symptoms, and Treatments. Cell Biochem. Biophys. 2015, 73, 291–296. 10.1007/s12013-015-0626-4. 25737133

[ref8] Liu N.; Colombi B.; Raytcheva-Buono E. V.; Bloise R.; Priori S. G. Catecholaminergic polymorphic ventricular tachycardia. Herz 2007, 32, 212–217. 10.1007/s00059-007-2975-2. 17497254

[ref9] Landstrom A. P.; Dobrev D.; Wehrens X. H. T. Calcium Signaling and Cardiac Arrhythmias. Circ. Res. 2017, 120, 1969–1993. 10.1161/CIRCRESAHA.117.310083. 28596175 PMC5607780

[ref10] Priori S. G.; Napolitano C.; Tiso N.; Memmi M.; Vignati G.; Bloise R.; Sorrentino V.; Danieli G. A. Mutations in the Cardiac Ryanodine Receptor Gene (hRyR2) Underlie Catecholaminergic Polymorphic Ventricular Tachycardia. Circulation 2001, 103, 196–200. 10.1161/01.CIR.103.2.196. 11208676

[ref11] George C. H.; Jundi H.; Thomas N. L.; Fry D. L.; Lai F. A. Ryanodine Receptors and Ventricular Arrhythmias: Emerging Trends in Mutations, Mechanisms and Therapies. J. Mol. Cell. Cardiol. 2007, 42, 34–50. 10.1016/j.yjmcc.2006.08.115. 17081562

[ref12] Postma A. V.; Denjoy I.; Kamblock J.; Alders M.; Lupoglazoff J. M.; Vaksmann G.; Dubosq-Bidot L.; Sebillon P.; Mannens M. M.; Guicheney P.; et al. Catecholaminergic Polymorphic Ventricular Tachycardia: RYR2 Mutations, Bradycardia, and Follow up of the Patients. J. Med. Genet. 2005, 42, 863–870. 10.1136/jmg.2004.028993. 16272262 PMC1735955

[ref13] Venetucci L.; Denegri M.; Napolitano C.; Priori S. G. Inherited Calcium Channelopathies in the Pathophysiology of Arrhythmias. Nat. Rev. Cardiol. 2012, 9, 561–575. 10.1038/nrcardio.2012.93. 22733215

[ref14] Marks A. R.; Priori S.; Memmi M.; Kontula K.; Laitinen P. J. Involvement of the Cardiac Ryanodine Receptor/Calcium Release Channel in Catecholaminergic Polymorphic Ventricular Tachycardia. J. Cell. Physiol. 2002, 190, 1–6. 10.1002/jcp.10031. 11807805

[ref15] Lehnart S. E.; Terrenoire C.; Reiken S.; Wehrens X. H.; Song L. S.; Tillman E. J.; Mancarella S.; Coromilas J.; Lederer W. J.; et al. Stabilization of Cardiac Ryanodine Receptor Prevents Intracellular Calcium Leak and Arrhythmias. Proc. Natl. Acad. Sci. U.S.A. 2006, 103, 7906–7910. 10.1073/pnas.0602133103. 16672364 PMC1472543

[ref16] Francis J.; Sankar V.; Nair V. K.; Priori S. G. Catecholaminergic Polymorphic Ventricular Tachycardia. Heart Rhythm 2005, 2, 550–554. 10.1016/j.hrthm.2005.01.024. 15840485

[ref17] Liu N.; Denegri M.; Dun W.; Boncompagni S.; Lodola F.; Protasi F.; Napolitano C.; Boyden P. A.; Priori S. G. Abnormal Propagation of Calcium Waves and Ultrastructural Remodeling in Recessive Catecholaminergic Polymorphic Ventricular Tachycardia. Circ. Res. 2013, 113, 142–152. 10.1161/CIRCRESAHA.113.301783. 23674379

[ref18] Herron T. J.; Milstein M. L.; Anumonwo J.; Priori S. G.; Jalife J. Purkinje Cell Calcium Dysregulation is the Cellular Mechanism that Underlies Catecholaminergic Polymorphic Ventricular Tachycardia. Heart Rhythm 2010, 7, 1122–1128. 10.1016/j.hrthm.2010.06.010. 20538074 PMC2910215

[ref19] Dulhunty A. F.; Casarotto M. G.; Beard N. A. The Ryanodine Receptor: A Pivotal Ca2+ Regulatory Protein and Potential Therapeutic Drug Target. Curr. Drug Targets 2011, 12, 709–723. 10.2174/138945011795378595. 21291389

[ref20] McCauley M. D.; Wehrens X. H. Targeting Ryanodine Receptors for Anti-Arrhythmic Therapy. Acta Pharmacol. Sin. 2011, 32, 749–757. 10.1038/aps.2011.44. 21642946 PMC4009959

[ref21] Serysheva I. I.; Orlova E. V.; Chiu W.; Sherman M. B.; Hamilton S. L.; van Heel M. Electron Cryomicroscopy and Angular Reconstitution Used to Visualize the Skeletal Muscle Calcium Release Channel. Nat. Struct. Biol. 1995, 2, 18–24. 10.1038/nsb0195-18. 7719847

[ref22] Serysheva I. I.; Schatz M.; van Heel M.; Chiu W.; Hamilton S. L. Structure of the Skeletal Muscle Calcium Release Channel Activated with Ca2+ and AMP-PCP. Biophys. J. 1999, 77, 1936–1944. 10.1016/S0006-3495(99)77035-9. 10512814 PMC1300475

[ref23] Serysheva I. I.; Hamilton S. L.; Chiu W.; Ludtke S. J. Structure of Ca2+ Release Channel at 14 Å Resolution. J. Mol. Biol. 2005, 345, 427–431. 10.1016/j.jmb.2004.10.073. 15581887 PMC2978512

[ref24] Ludtke S. J.; Serysheva I. I.; Hamilton S. L.; Chiu W. The Pore Structure of the Closed RyR1 Channel. Structure 2005, 13, 1203–1211. 10.1016/j.str.2005.06.005. 16084392 PMC2983469

[ref25] Samsó M.; Wagenknecht T.; Allen P. D. Internal Structure and Visualization of Transmembrane Domains of the RyR1 Calcium Release Channel by Cryo-EM. Nat. Struct. Mol. Biol. 2005, 12, 539–544. 10.1038/nsmb938. 15908964 PMC1925259

[ref26] Welch W.; Rheault S.; West D. J.; Williams A. J. A Model of the Putative Pore Region of the Cardiac Ryanodine Receptor Channel. Biophys. J. 2004, 87, 2335–2351. 10.1529/biophysj.104.044180. 15454434 PMC1304657

[ref27] Samsó M.; Feng W.; Pessah I. N.; Allen P. D. Coordinated Movement of Cytoplasmic and Transmembrane Domains of RyR1 Upon Gating. PLoS Biol. 2009, 7, e1000085 10.1371/journal.pbio.1000085. 19402748 PMC2672603

[ref28] Ramachandran S.; Chakraborty A.; Xu L.; Mei Y.; Samso M.; Dokholyan N. V.; Meissner G. Structural Determinants of Skeletal Muscle Ryanodine Receptor Gating. J. Biol. Chem. 2013, 288, 6154–6165. 10.1074/jbc.M112.433789. 23319589 PMC3585052

[ref29] Zalk R.; Clarke O. B.; des Georges A.; Grassucci R. A.; Reiken S.; Mancia F.; Hendrickson W. A.; Frank J.; Marks A. R. Structure of a Mammalian Ryanodine Receptor. Nature 2015, 517, 44–49. 10.1038/nature13950. 25470061 PMC4300236

[ref30] Yan Z.; Bai X.; Yan C.; Wu J.; Li Z.; Xie T.; Peng W.; Yin C.; Li X.; Scheres S. H. W.; et al. Structure of the Rabbit Ryanodine Receptor RyR1 at Near-Atomic Resolution. Nature 2015, 517, 50–55. 10.1038/nature14063. 25517095 PMC4338550

[ref31] Zheng W. Toward Decrypting the Allosteric Mechanism of the Ryanodine Receptor Based on Coarse-Grained Structural and Dynamic Modeling. Proteins 2015, 83, 2307–2318. 10.1002/prot.24951. 26492335

[ref32] Peng W.; Shen H.; Wu J.; Guo W.; Pan X.; Wang R.; Chen S. R.; Yan N. Structural Basis for the Gating Mechanism of the Type 2 Ryanodine Receptor RyR2. Science 2016, 354, eaah5324 10.1126/science.aah5324. 27708056

[ref33] des Georges A.; Clarke O. B.; Zalk R.; Yuan Q.; Condon K. J.; Grassucci R. A.; Hendrickson W. A.; Marks A. R.; Frank J. Structural Basis for Gating and Activation of RyR1. Cell 2016, 167, 145–157.e17. 10.1016/j.cell.2016.08.075. 27662087 PMC5142848

[ref34] Bai X. C.; Yan Z.; Wu J.; Li Z.; Yan N. The Central Domain of RyR1 is the Transducer for Long-Range Allosteric Gating of Channel Opening. Cell Res. 2016, 26, 995–1006. 10.1038/cr.2016.89. 27468892 PMC5034110

[ref35] Samsó M. A Guide to the 3D Structure of the Ryanodine Receptor Type 1 by CryoEM. Protein Sci. 2017, 26, 52–68. 10.1002/pro.3052. 27671094 PMC5192967

[ref36] Gong D.; Chi X.; Wei J.; Zhou G.; Huang G.; Zhang L.; Wang R.; Lei J.; Chen S. R. W.; Yan N. Modulation of Cardiac Ryanodine Receptor 2 by Calmodulin. Nature 2019, 572, 347–351. 10.1038/s41586-019-1377-y. 31278385

[ref37] Ogawa H.; Kurebayashi N.; Yamazawa T.; Murayama T. Regulatory Mechanisms of Ryanodine Receptor/Ca(2+) Release Channel Revealed by Recent Advancements in Structural Studies. J. Muscle Res. Cell Motil. 2021, 42, 291–304. 10.1007/s10974-020-09575-6. 32040690 PMC8332584

[ref38] Mowrey D. D.; Xu L.; Mei Y.; Pasek D. A.; Meissner G.; Dokholyan N. V. Ion-Pulling Simulations Provide Insights into the Mechanisms of Channel Opening of the Skeletal Muscle Ryanodine Receptor. J. Biol. Chem. 2017, 292, 12947–12958. 10.1074/jbc.M116.760199. 28584051 PMC5546034

[ref39] Schilling R.; Fink R. H.; Fischer W. B. MD Simulations of the Central Pore of Ryanodine Receptors and Sequence Comparison with 2B Protein from Coxsackie Virus. Biochim. Biophys. Acta 2014, 1838, 1122–1131. 10.1016/j.bbamem.2013.12.008. 24365119

[ref40] Shirvanyants D.; Ramachandran S.; Mei Y.; Xu L.; Meissner G.; Dokholyan N. V. Pore Dynamics and Conductance of RyR1 Transmembrane Domain. Biophys. J. 2014, 106, 2375–2384. 10.1016/j.bpj.2014.04.023. 24896116 PMC4052289

[ref41] Isralewitz B.; Gao M.; Schulten K. Steered Molecular Dynamics and Mechanical Functions of Proteins. Curr. Opin. Struct. Biol. 2001, 11, 224–230. 10.1016/S0959-440X(00)00194-9. 11297932

[ref42] Do P. C.; Lee E. H.; Le L. Steered Molecular Dynamics Simulation in Rational Drug Design. J. Chem. Inf. Model. 2018, 58, 1473–1482. 10.1021/acs.jcim.8b00261. 29975531

[ref43] Musgaard M.; Biggin P. C. Steered Molecular Dynamics Simulations Predict Conformational Stability of Glutamate Receptors. J. Chem. Inf. Model. 2016, 56, 1787–1797. 10.1021/acs.jcim.6b00297. 27482759

[ref44] Gunnoo M.; Cazade P. A.; Orlowski A.; Chwastyk M.; Liu H.; Ta D. T.; Cieplak M.; Nash M.; Thompson D. Steered Molecular Dynamics Simulations Reveal the Role of Ca(2+) in Regulating Mechanostability of Cellulose-Binding Proteins. Phys. Chem. Chem. Phys. 2018, 20, 22674–22680. 10.1039/C8CP00925B. 30132772

[ref45] Patel J. S.; Berteotti A.; Ronsisvalle S.; Rocchia W.; Cavalli A. Steered Molecular Dynamics Simulations for Studying Protein-Ligand Interaction in Cyclin-Dependent Kinase 5. J. Chem. Inf. Model. 2014, 54, 470–480. 10.1021/ci4003574. 24437446

[ref46] Xiao B. L.; Ning Y. N.; Niu N. N.; Li D.; Moosavi-Movahedi A. A.; Sheibani N.; Hong J. Steered Molecular Dynamic Simulations of Conformational Lock of Cu, Zn-Superoxide Dismutase. Sci. Rep. 2019, 9, 4353 10.1038/s41598-019-40892-0. 30867507 PMC6416402

[ref47] Nury H.; Poitevin F.; Van Renterghem C.; Changeux J. P.; Corringer P. J.; Delarue M.; Baaden M. One-Microsecond Molecular Dynamics Simulation of Channel Gating in a Nicotinic Receptor Homologue. Proc. Natl. Acad. Sci. U.S.A. 2010, 107, 6275–6280. 10.1073/pnas.1001832107. 20308576 PMC2852019

[ref48] Bjelkmar P.; Niemela P. S.; Vattulainen I.; Lindahl E. Conformational Changes and Slow Dynamics Through Microsecond Polarized Atomistic Molecular Simulation of an Integral Kv1.2 Ion Channel. PLoS Comput. Biol. 2009, 5, e1000289 10.1371/journal.pcbi.1000289. 19229308 PMC2632863

[ref49] Burley S. K.; Berman H. M.; Bhikadiya C.; Bi C.; Chen L.; Di Costanzo L.; Christie C.; Dalenberg K.; Duarte J. M.; Dutta S.; et al. RCSB Protein Data Bank: Biological Macromolecular Structures Enabling Research and Education in Fundamental Biology, Biomedicine, Biotechnology and Energy. Nucleic Acids Res. 2019, 47, D464–D474. 10.1093/nar/gky1004. 30357411 PMC6324064

[ref50] Heinz L. P.; Kopec W.; de Groot B. L.; Fink R. H. A. In Silico Assessment of the Conduction Mechanism of the Ryanodine Receptor 1 Reveals Previously Unknown Exit Pathways. Sci. Rep. 2018, 8, 6886 10.1038/s41598-018-25061-z. 29720700 PMC5932038

[ref51] Apweiler R.; Bairoch A.; Wu C. H.; Barker W. C.; Boeckmann B.; Ferro S.; Gasteiger E.; Huang H.; Lopez R.; Magrane M.; et al. UniProt: the Universal Protein Knowledgebase. Nucleic Acids Res. 2004, 32, D115–D119. 10.1093/nar/gkh131. 14681372 PMC308865

[ref52] Wu E. L.; Cheng X.; Jo S.; Rui H.; Song K. C.; Davila-Contreras E. M.; Qi Y.; Lee J.; Monje-Galvan V.; Venable R. M.; et al. CHARMM-GUI Membrane Builder Toward Realistic Biological Membrane Simulations. J. Comput. Chem. 2014, 35, 1997–2004. 10.1002/jcc.23702. 25130509 PMC4165794

[ref53] Madej B.; Walker R. An Amber Lipid Force Field Tutorial: Lipid 14 Edition. http://ambermd.org/tutorials/advanced/tutorial16/ (accessed July 11, 2022).

[ref54] Pettersen E. F.; Goddard T. D.; Huang C. C.; Couch G. S.; Greenblatt D. M.; Meng E. C.; Ferrin T. E. UCSF Chimera--A Visualization System for Exploratory Research and Analysis. J. Comput. Chem. 2004, 25, 1605–1612. 10.1002/jcc.20084. 15264254

[ref55] Fiser A.; Sali A. Modeller: Generation and Refinement of Homology-Based Protein Structure Models. Methods Enzymol. 2003, 374, 461–491. 10.1016/S0076-6879(03)74020-8. 14696385

[ref56] Webb B.; Sali A. Comparative Protein Structure Modeling Using MODELLER. Curr. Protoc. Bioinform. 2016, 54, 5.6.1–5.6.37. 10.1002/cpbi.3. PMC5031415 27322406

[ref57] Case D. A.; Cheatham T. E. 3rd; Darden T.; Gohlke H.; Luo R.; Merz K. M. Jr.; Onufriev A.; Simmerling C.; Wang B.; Woods R. J. The Amber Biomolecular Simulation Programs. J. Comput. Chem. 2005, 26, 1668–1688. 10.1002/jcc.20290. 16200636 PMC1989667

[ref58] Case D. A.; Ben-Shalom I. Y.; Brozell S. R.; Cerutti D. S.; Cheatham T. E. 3rd; Cruzeiro V. W. D.; Darden T. A.; Duke R. E.; Ghoreishi D.; Gilson M. K. AMBER 19; University of California: San Francisco, 2019.

[ref59] Götz A. W.; Williamson M. J.; Xu D.; Poole D.; Le Grand S.; Walker R. C. Routine Microsecond Molecular Dynamics Simulations with AMBER on GPUs. 1. Generalized Born. J. Chem. Theory Comput. 2012, 8, 1542–1555. 10.1021/ct200909j. 22582031 PMC3348677

[ref60] Smart O. S.; Goodfellow J. M.; Wallace B. A. The Pore Dimensions of Gramicidin A. Biophys. J. 1993, 65, 2455–2460. 10.1016/S0006-3495(93)81293-1. 7508762 PMC1225986

[ref61] Jeffrey G. A. An Introduction to Hydrogen Bonding; Oxford University Press: Oxford, U.K., 1997.

[ref62] Greene D.; Barton M.; Luchko T.; Shiferaw Y. Computational Analysis of Binding Interactions between the Ryanodine Receptor Type 2 and Calmodulin. J. Phys. Chem. B 2021, 125, 10720–10735. 10.1021/acs.jpcb.1c03896. 34533024

[ref63] Chen J.; Mitcheson J. S.; Tristani-Firouzi M.; Lin M.; Sanguinetti M. C. The S4-S5 Linker Couples Voltage Sensing and Activation of Pacemaker Channels. Proc. Natl. Acad. Sci. U.S.A. 2001, 98, 11277–11282. 10.1073/pnas.201250598. 11553787 PMC58720

[ref64] Tristani-Firouzi M.; Chen J.; Sanguinetti M. C. Interactions Between S4-S5 Linker and S6 Transmembrane Domain Modulate Gating of HERG K+ Channels. J. Biol. Chem. 2002, 277, 18994–19000. 10.1074/jbc.M200410200. 11864984

[ref65] Lu Z.; Klem A. M.; Ramu Y. Coupling Between Voltage Sensors and Activation Gate in Voltage-Gated K+ Channels. J. Gen. Physiol. 2002, 120, 663–676. 10.1085/jgp.20028696. 12407078 PMC2229552

[ref66] Decher N.; Chen J.; Sanguinetti M. C. Voltage-Dependent Gating of Hyperpolarization-Activated, Cyclic Nucleotide-Gated Pacemaker Channels: Molecular Coupling Between the S4-S5 and C-linkers. J. Biol. Chem. 2004, 279, 13859–13865. 10.1074/jbc.M313704200. 14726518

[ref67] Ferrer T.; Rupp J.; Piper D. R.; Tristani-Firouzi M. The S4-S5 Linker Directly Couples Voltage Sensor Movement to the Activation Gate in the Human Ether-A’-Go-Go-Related Gene (hERG) K+ Channel. J. Biol. Chem. 2006, 281, 12858–12864. 10.1074/jbc.M513518200. 16524878

[ref68] Uehara A.; Murayama T.; Yasukochi M.; Fill M.; Horie M.; Okamoto T.; Matsuura Y.; Uehara K.; Fujimoto T.; Sakurai T.; Kurebayashi N. Extensive Ca2+ Leak Through K4750Q Cardiac Ryanodine Receptors Caused by Cytosolic and Luminal Ca2+ Hypersensitivity. J. Gen. Physiol. 2017, 149, 199–218. 10.1085/jgp.201611624. 28082361 PMC5299618

[ref69] Naritomi Y.; Fuchigami S. Slow dynamics of a protein backbone in molecular dynamics simulation revealed by time-structure based independent component analysis. J. Chem. Phys. 2013, 139, 215102 10.1063/1.4834695. 24320404

[ref70] Guo W.; Wei J.; Estillore J. P.; Zhang L.; Wang R.; Sun B.; Chen S. R. W. RyR2 Disease Mutations at the C-terminal Domain Intersubunit Interface Alter Closed-State Stability and Channel Activation. J. Biol. Chem. 2021, 297, 100808 10.1016/j.jbc.2021.100808. 34022226 PMC8214192

[ref71] Ovchinnikov V.; Karplus M. Analysis and Elimination of a Bias in Targeted Molecular Dynamics Simulations of Conformational Transitions: Application to Calmodulin. J. Phys. Chem. B 2012, 116, 8584–8603. 10.1021/jp212634z. 22409258 PMC3406239

