# Supplementary material for: Molecular Dynamics Simulations of the Cardiac Ryanodine Receptor Type 2 (RyR2) Gating Mechanism
Source: J Phys Chem B. 2022 Nov 16;126(47):9790–809. doi: 10.1021/acs.jpcb.2c03031 (PMC9720719; doi:10.1021/acs.jpcb.2c03031)
Supplement: Supplementary file 1 — jp2c03031_si_001.pdf [file jp2c03031_si_001.pdf]

Supporting Information

Molecular Dynamics Simulations of the Cardiac Ryanodine Receptor Type 2 (RyR2) Gating Mechanism

D'Artagnan Greene*, Michael Barton, Tyler Luchko, and Yohannes Shiferaw

Department of Physics and Astronomy,
California State University, Northridge, CA 91330, United States of America

* Please send correspondence to: dartagnan.greene@csun.edu

FIGURES

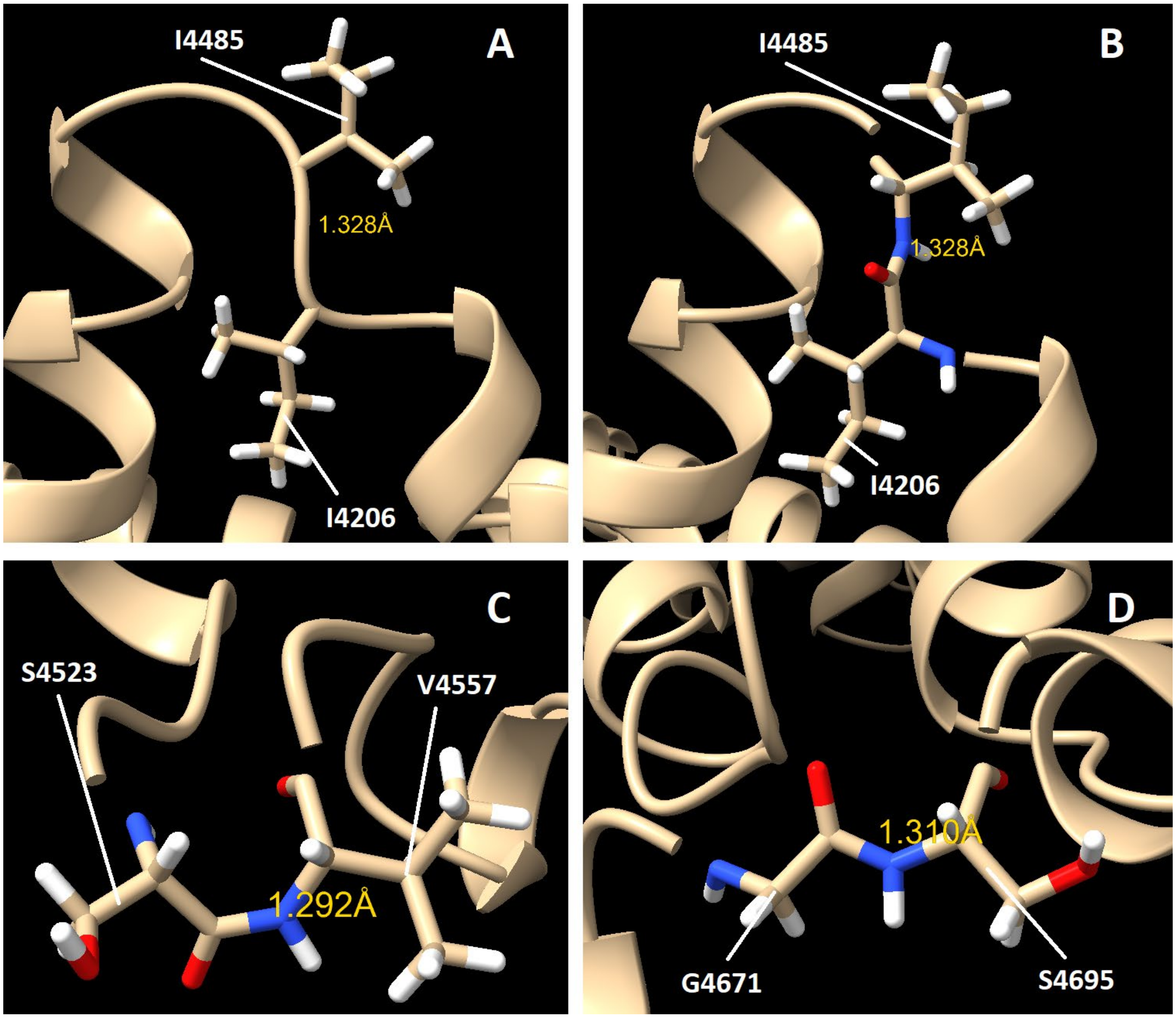

FIGURE S1. Peptide bonds formed between the ends of missing sequences within the RyR2 model systems. Peptide bonds between the end residues of missing sequences are shown from the last frame of the 4C structure at the end of the 1 µs MD simulation. In (A) and (B), I4206 is connected by a peptide bond to I4485. In (B), the loop connecting this region was hidden to allow the connection between backbone atoms to be seen explicitly. In (C), S4523 is shown connected by a peptide bond to V4557, and in (D), G4671 is connected by a peptide bond to S4695. In (C) and (D), the loop connecting this region was hidden to allow the connection between backbone atoms to be seen explicitly. Peptide bond distances between the nitrogen in NH and carbon in CO were measured using UCSF Chimera X 1.4.

A

```
                                                 4100
                                                   AV
       4110       4120       4130       4140       4150
LLTNLSEHMP NDTRLQTFLE LAESVLNYFQ PFLGRIEIMG SAKRIERVYF
       4160       4170       4180       4190       4200
EISESSRTQW EKPQVKESKR QFIFDVVNEG GEKEKMELFV NFCEDTIFEM
       4210
QLAAQI
```

B

```
                                      4490       4500
                                    IAYQQK LLNYFARNFY
       4510       4520       4530       4540       4550
NMRMLALFVA FAINFILLFY KVSTSSVVEG KELPTRSSSE NAKVTSLDSS
       4560       4570       4580       4590       4600
SHRIIAVHYV LEESSGYMEP TLRILAILHT VISFFCIIGY YCLKVPLVIF
       4610       4620       4630       4640       4650
KREKEVARKL EFDGLYITEQ PSEDDIKGQW DRLVINTQSF PNNYWDKFVK
       4660       4670       4680       4690       4700
RKVMDKYGEF YGRDRISELL GMDKAALDFS DAREKKKPKK DSSLSAVLNS
       4710       4720       4730       4740       4750
IDVKYQMWKL GVVFTDNSFL YLAWYMTMSV LGHYNNFFFA AHLLDIAMGF
       4760       4770       4780       4790       4800
KTLRTILSSV THNGKQLVLT VGLLAVVVYL YTVVAFNFFR KFYNKSEDGD
       4810       4820       4830       4840       4850
TPDMKCDDML TCYMFHMYVG VRAGGGIGDE IEDPAGDEYE IYRIIFDITF
       4860       4870       4880       4890       4900
FFFVIVILLA IIQGLIIDAF GELRDQQEQV KEDMETKCFI CGIGNDYFDT
       4910       4920       4930       4940       4950
VPHGFETHTL QEHNLANYLF FLMYLINKDE TEHTGQESYV WKMYQERCWE
       4960
FFPAGDCFRK QYE
```

FIGURE S2. Sequence of amino acid residues for a single subunit within the RyR2 model systems. The sequence of amino acid residues for a single subunit within the RyR2 model systems is provided above. Residues 4099-4206, containing a portion of the cytosolic domain that includes the U-motif, are given in (A) while residues 4485-4963, containing the transmembrane channel domain and a portion of the C-terminal domain, are given in (B). Missing disordered segments in the channel domain, corresponding to residues 4524-4556 and 4672-4694, are underlined in red while residue 4523, which is only missing in the open system subunits, is underlined in blue. The S4S5L (residues 4746-4766) is underlined in green, and the location of the H4762P mutation is underlined in orange. The central portion of the S6 helix (residues 4859-4869), is underlined in purple. The location of the isoleucine residue from the hydrophobic gate (I4867) is underlined in yellow. Residue numbers correspond to the canonical human RyR2 numbering sequence as depicted in the Uniprot database (isoform 1, identifier: Q92736-1).

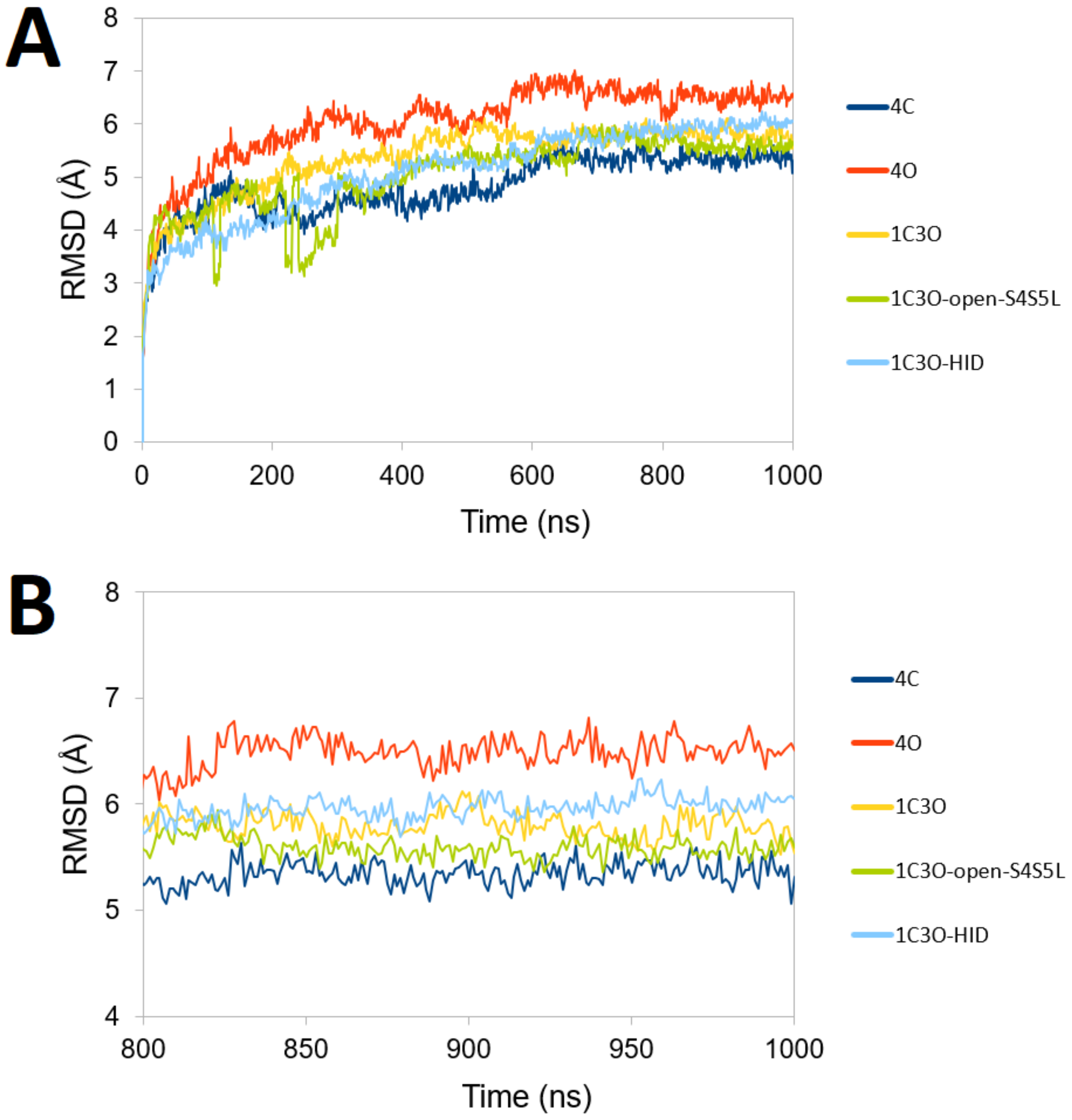

FIGURE S3. RMSD plots of model RyR2 systems (part 1). RMSD plots for the full 1 µs MD trajectory are provided in (A) for 4C (blue), 4O (orange), 1C3O (yellow), 1C3O-open-S4S5L (light green), and 1C3O-HID (light blue). A closeup of the last 200 ns is provided in (B). The RMSD was calculated with respect to the RyR2 backbone carbon atoms (CA) in the initial frame of the MD trajectory for each system using all 1,000 frames sampled at even intervals over 1 µs.

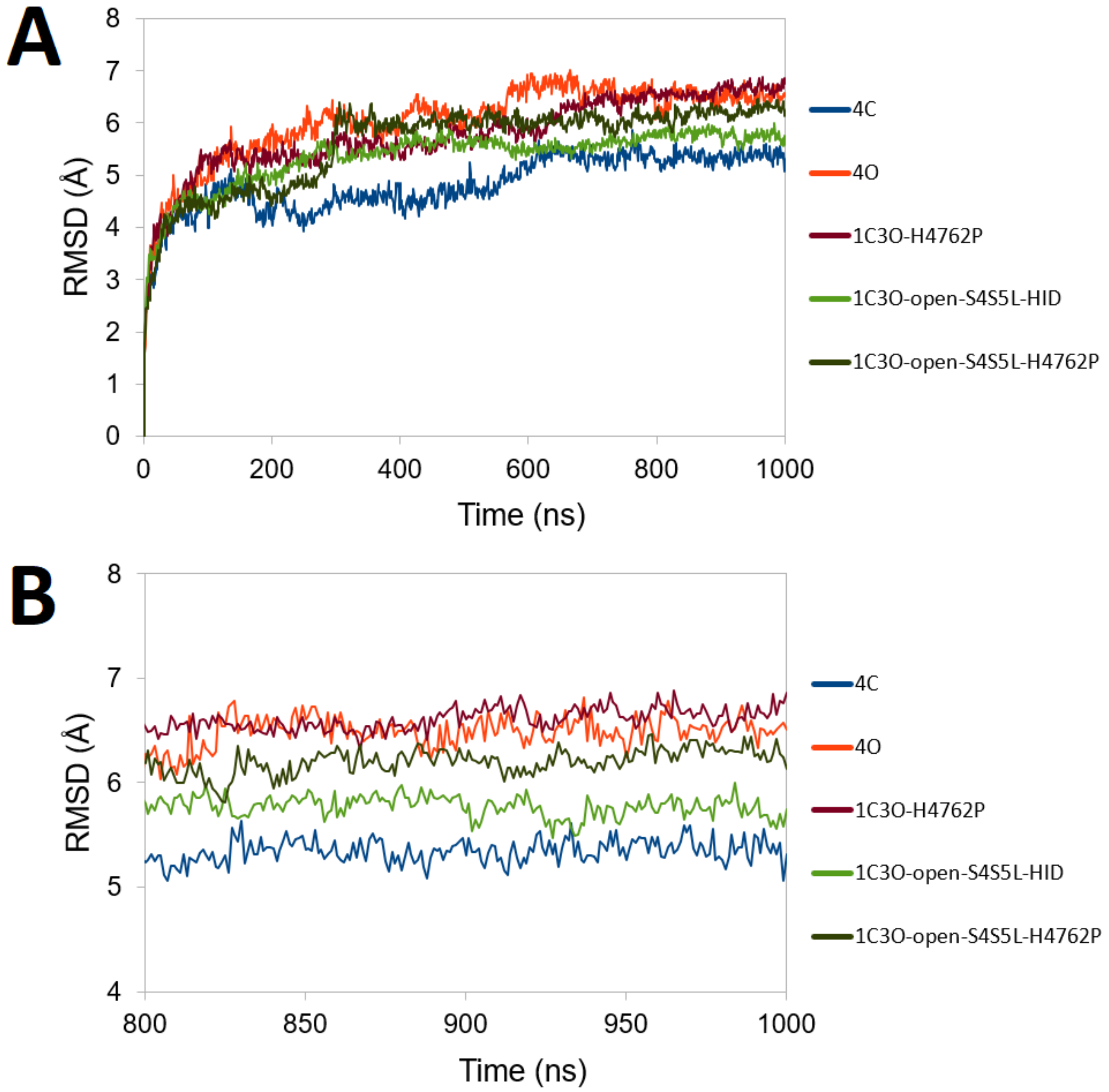

FIGURE S4. RMSD plots of model RyR2 systems (part 2). RMSD plots for the full 1 µs MD trajectory are provided in (A) for 4C (blue), 4O (orange), 1C3O-H4762P (dark red), 1C3O-open-S4S5L-HID (green), and 1C3O-open-S4S5L-H4762P (dark green). A close up of the last 200 ns is provided in (B). The RMSD was calculated with respect to the RyR2 backbone carbon atoms (CA) in the initial frame of the MD trajectory for each system using all 1,000 frames sampled at even intervals over 1 µs.

A

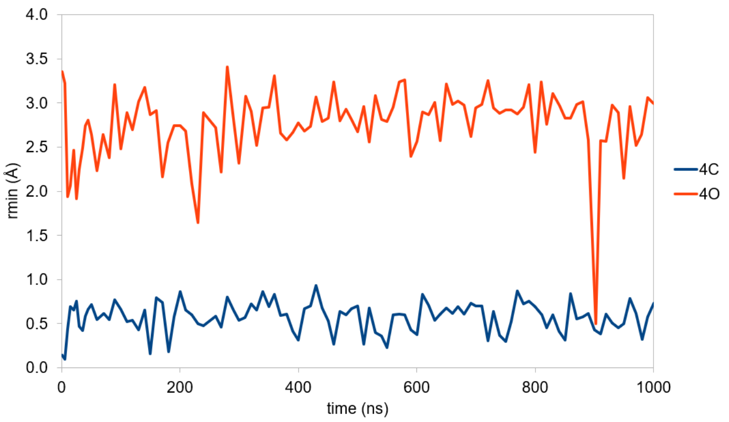

B

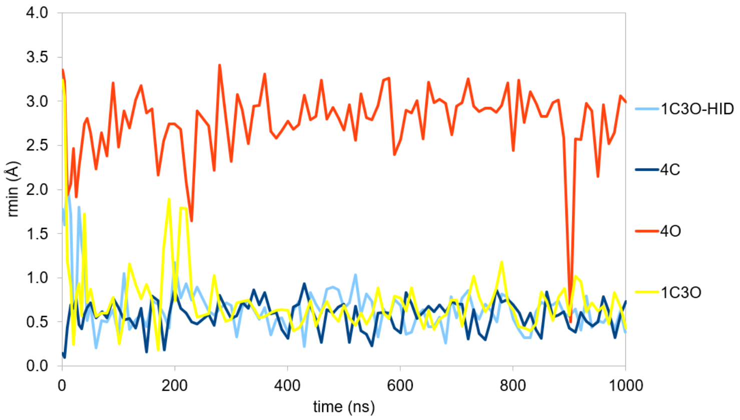

FIGURE S5. Plots of the calculated minimum radius for RyR2 systems (part 1). Results of the minimum radius calculated using the Hole software for RyR2 systems across the full 1 μs trajectory are provided above. A maximum cutoff radius of 10 Å was specified in these calculations. In order to improve the legibility of these plots, we report data every 5 ns until the 50 ns mark, and then sample data at 10 ns intervals afterwards. To reduce the instances of temporary noise being reported in the sampling at these 10 ns intervals, we replaced any data point with a minimum pore radius of < 0.3 Å with the value given at the following ns. This noise is due to the Hole calculation identifying a transient crevice as being a part of the pore. In instances where this occurs, the minimum radius value sharply drops towards 0 Å for 1-2 ns, but the general trend returns immediately afterwards. This happens quite frequently in both the closed and open systems, but the effect is much more noticeable in our open state systems because the baseline value of the minimum radius is much higher than it is for the closed state systems. We note that the minimum radius does not always represent the true radius of the hydrophobic gate as we discussed in our results (see **FIGURES 3** and **4**). We also note that the instances of noise that appear do not reflect any noticeable structural instability in the RyR2 channel, as both the channel and the hydrophobic gate were confirmed to be stable using a visual inspection of the trajectory. The global stability of each system is also very evident in our RMSD analysis given in **FIGURES S3** and **S4**.

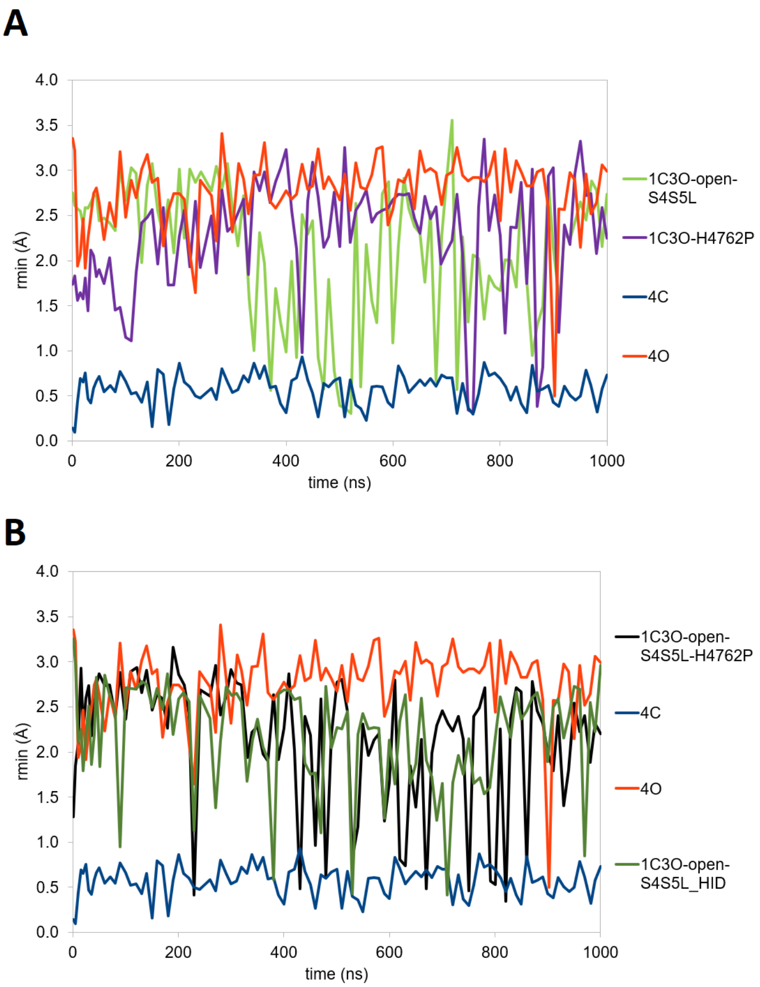

FIGURE S6. Plots of the calculated minimum radius for RyR2 systems (part 2). Results of the minimum radius calculated using the Hole software for RyR2 systems across the full 1 µs trajectory are provided above. A maximum cutoff radius of 10 Å was specified in these calculations. In order to improve the legibility of these plots, we report data every 5 ns until the 50 ns mark, and then sample data at 10 ns intervals afterwards. To reduce the instances of temporary noise being reported in the sampling at these 10 ns intervals, we replaced any data point with a minimum pore radius of < 0.3 Å with the value given at the following ns. This noise is due to the Hole calculation identifying a transient crevice as being a part of the pore. In instances where this occurs, the minimum radius value sharply drops towards 0 Å for 1-2 ns, but the general trend returns immediately afterwards. This happens quite frequently in both the closed and open systems, but the effect is much more noticeable in our open state systems because the baseline value of the minimum radius is much higher than it is for the closed state systems. We note that the minimum radius does not always represent the true radius of the hydrophobic gate as we discussed in our results (see **FIGURES 3** and **4**). We also note that the instances of noise that appear do not reflect any noticeable structural instability in the RyR2 channel, as both the channel and the hydrophobic gate were confirmed to be stable using a visual inspection of the trajectory. The global stability of each system is also very evident in our RMSD analysis given in **FIGURES S3** and **S4**.

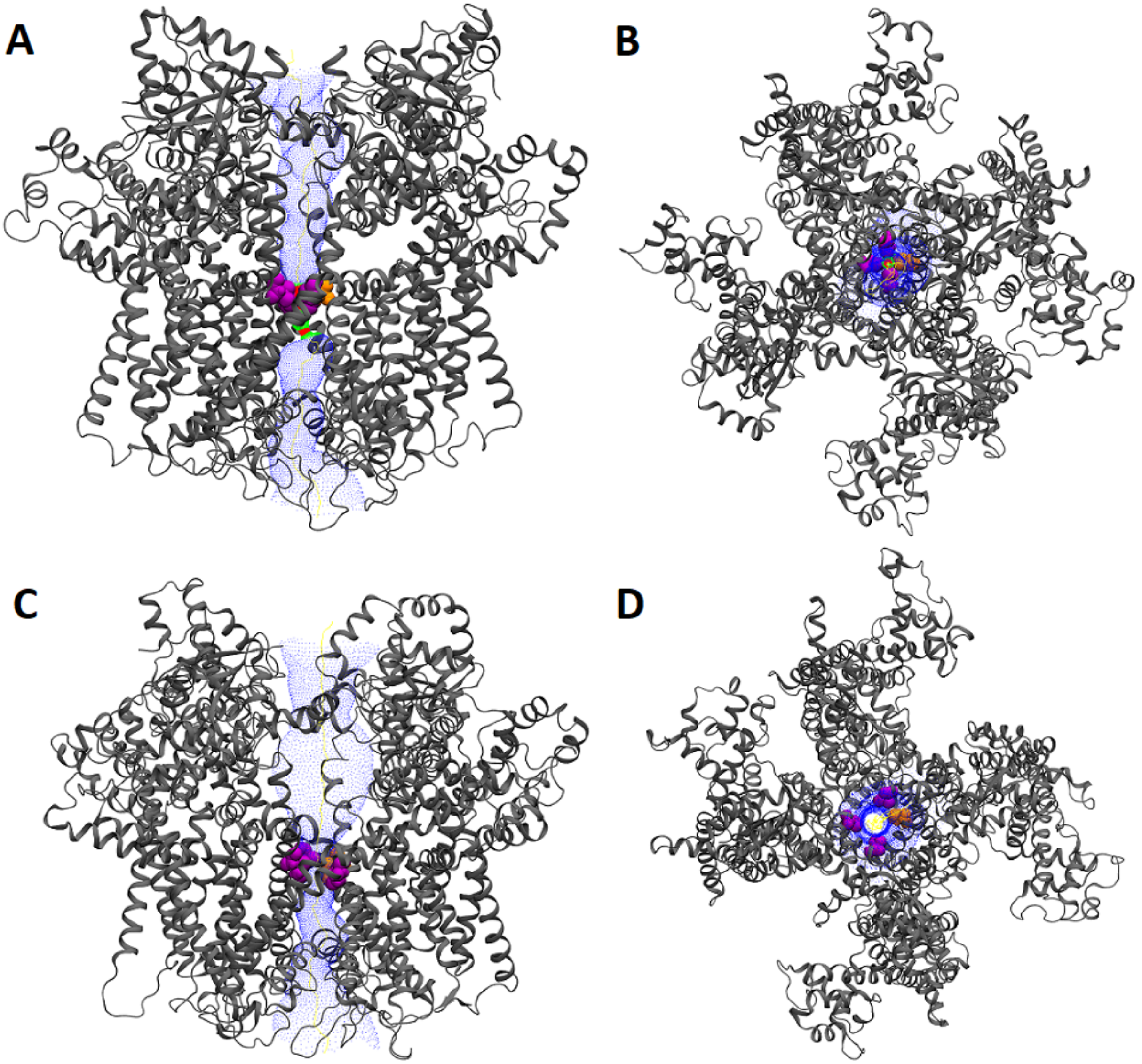

FIGURE S7. Structures of the closed and open RyR2 systems. The structure of the closed 4C RyR2 system is shown in a side view in (A). The cytosolic end of the channel appears at the top, and the luminal end is at the bottom. A top-down view looking down from the cytosolic end of the channel in shown in (B). Similarly, the structure of the open 4O RyR2 system is shown in a side view in (C) and a top-down view in (D). The hydrophobic center of the closed channel pore (I4867), is depicted using orange VDW spheres for the first subunit and purple VDW spheres for the other three subunits. The channel pore is visualized down the center of each channel with a colored dot surface using the Hole software. The color of the dot surface indicates the size of the pore radius, with red being the most constricted portion and blue the least.

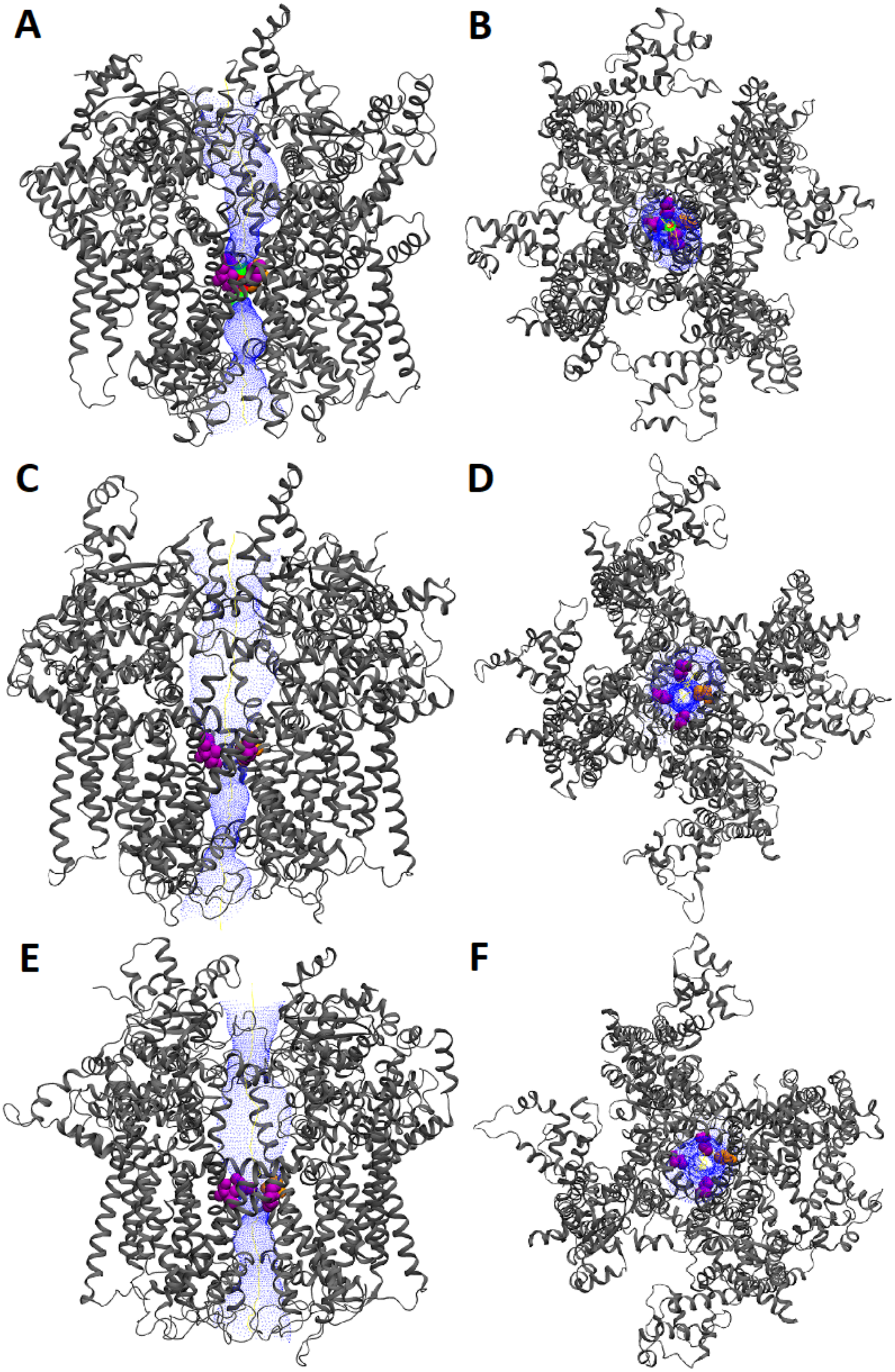
A
B
C
D
E
F

FIGURE S8. Structures of the chimera RyR2 systems (prior page). The structure for the chimera RyR2 system (1C3O), where initially the first subunit was in the closed conformation and the other three subunits were in the open conformation is shown in a side view in (A). In (A), the cytosolic end of the channel appears at the top, and a top-down view from the cytosolic end is given in (B). The structure of a variant of the 1C3O chimera system where the S4S5L (residues 4746-4766) within the closed subunit was replaced with the S4S5L from the open subunit (1C3O-open-S4S5L) is shown in (C) and (D). The structure of a variant 1C3O system where a single point mutation was made to the closed subunit (1C3O-H4762P) is shown in (E) and (F). The hydrophobic center of the closed channel pore (I4867) is depicted using orange VDW spheres on the first subunit and purple VDW spheres on the other three subunits. The channel pore is visualized down the center of each channel with a colored dot surface using the Hole software. The color of the dot surface indicates the size of the pore radius, with red being the most constricted portion and blue the least.

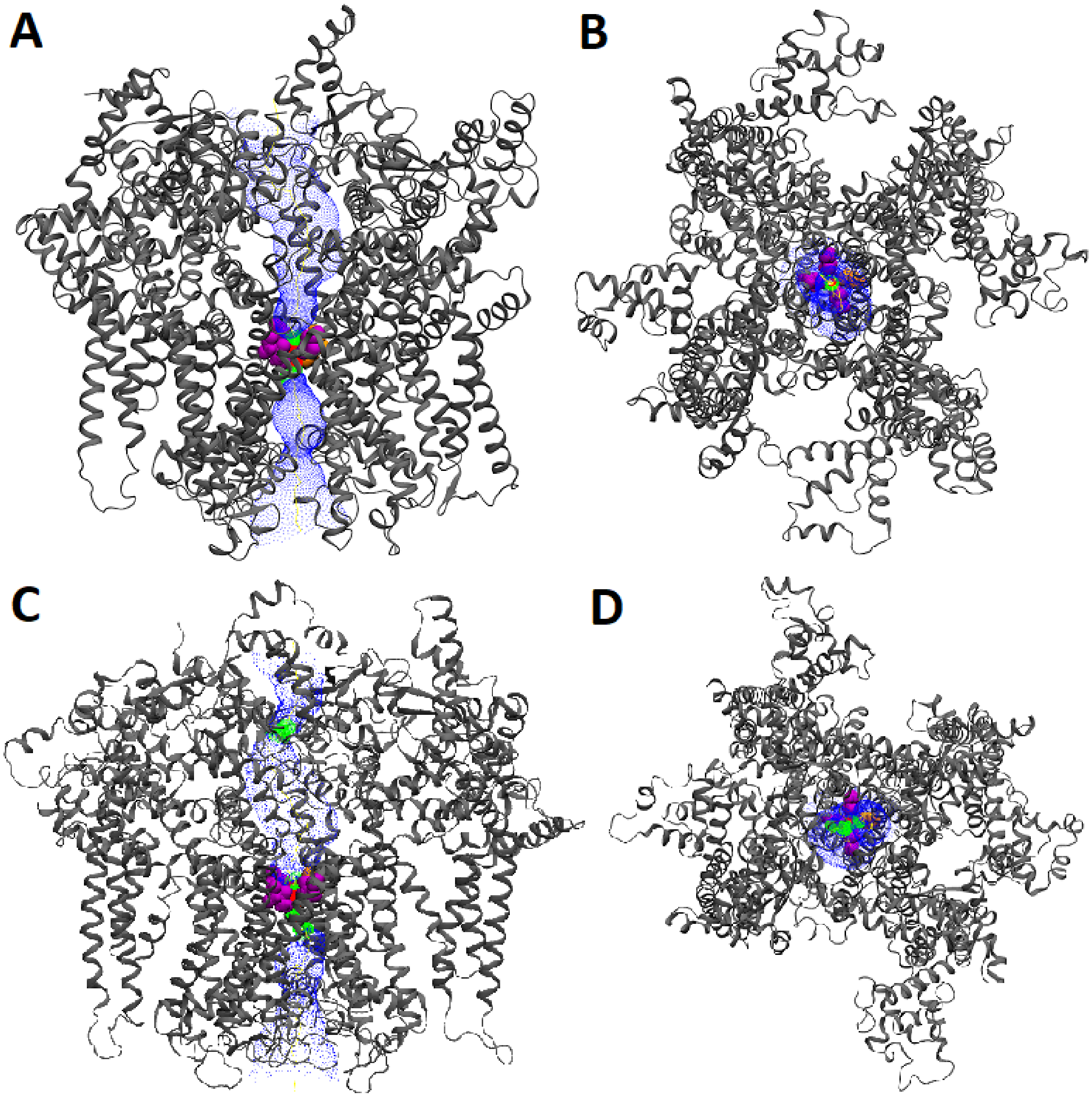

FIGURE S9. Structures of closed state chimera RyR2 systems. The structure of the 1C3O chimera RyR2 system, where initially the first subunit was in the closed conformation and the other three subunits were in the open conformation, is shown in a side view in (A) and a top down view in (B), with the cytosolic end of the channel at the top. A replica run (1C3O-HID), where the only change from the prior run was changing the position of a hydrogen atom on histidine residue 4762 from epsilon (HIE) to delta (HID), is shown in (C) and (D). The hydrophobic center of the closed channel pore is depicted using orange VDW spheres for the I4867 residue on the first subunit and purple VDW spheres on the other three subunits. The channel pore is visualized down the center of each channel with a colored dot surface using the Hole software. The color of the dot surface indicates the size of the pore radius, with red being the most constricted portion and blue the least.

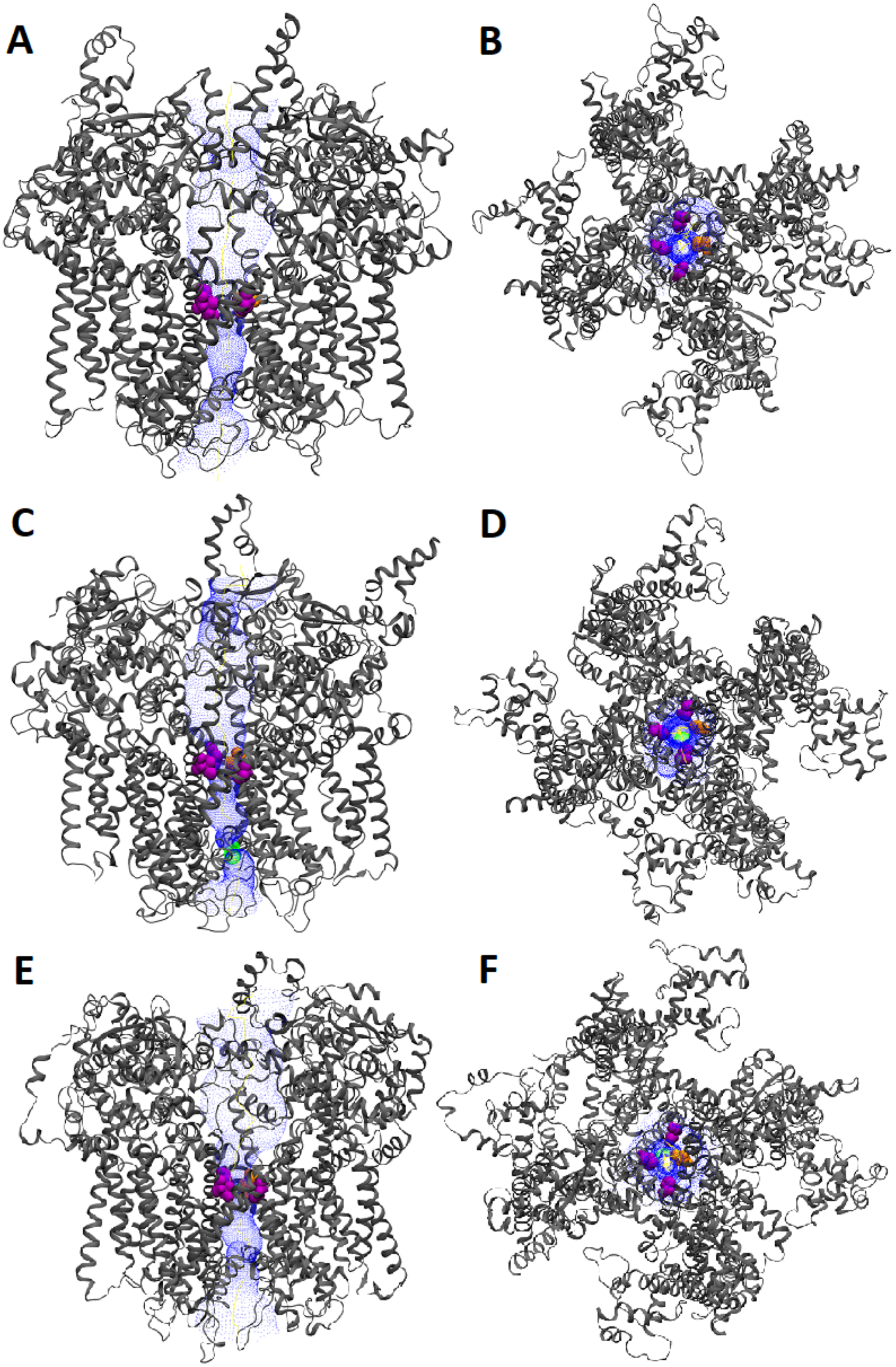
A
B
C
D
E
F

FIGURE S10. Structures of open state chimera RyR2 systems (prior page). The structure of the 1C3O system where the S4S5L (residues 4746-4766) within the closed subunit was replaced with the S4S5L from the open subunit (1C3O-open-S4S5L) is shown in (A) and (B). In (A), the cytosolic end of the channel is shown at the top while (B) gives a top-down view from the cytosolic end. A replica run (1C3O-open-S4S5L-HID), where the only change from the prior run was changing the position of a hydrogen atom on histidine residue 4762 from epsilon (HIE) to delta (HID), is shown in (C) and (D). An additional run, where the H4762P mutation was made in the S4S5L linker as it was transposed into the closed subunit (1C3O-open-S4S5L-H4762P), is shown in (E) and (F). The hydrophobic center of the closed channel pore is depicted using orange VDW spheres for the I4867 residue on the first subunit and purple VDW spheres on the other three subunits. The channel pore is visualized down the center of each channel with a colored dot surface using the Hole software. The color of the dot surface indicates the size of the pore radius, with red being the most constricted portion and blue the least.

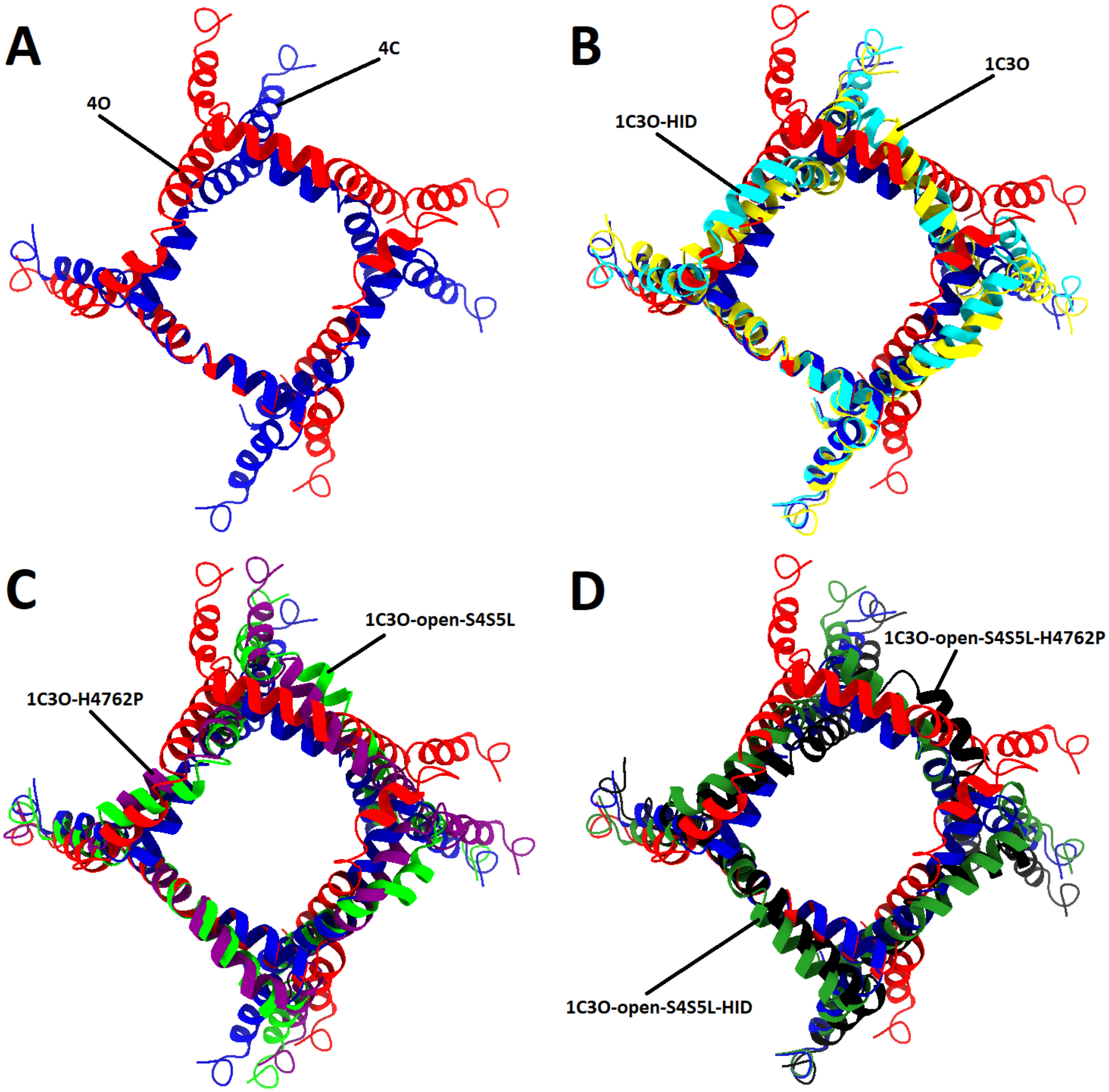

FIGURE S11. Alignment of the S4S5L gating loop for each model RyR2 system with respect to the 4C system. In A), the 4-subunit S4S5L gating loop structures (residues 4731-4781) from the 4C (blue) and 4O (red) systems are shown aligned with the 4C system. In B), the same residues for the closed state chimera systems 1C3O (yellow) and 1C3O-HID (cyan) are shown aligned with the 4C system (blue) while the 4O system (red) is also present for comparison. In C), the same residues for the 1C3O-open-S4S5L (green) and 1C3O-H4762P (magenta) systems are shown aligned with the 4C system (blue) while the 4O system (red) is also present for comparison. In D), the same residues for the 1C3O-open-S4S5L-HID (dark green) and 1C3O-open-S4S5L-H4762P (black) systems are shown aligned with the 4C system (blue) while the 4O system (red) is also present for comparison. The alignments were produced with Match Maker in UCSF Chimera 1.15 using the best aligned pair of chains with reference to the 4C structure.

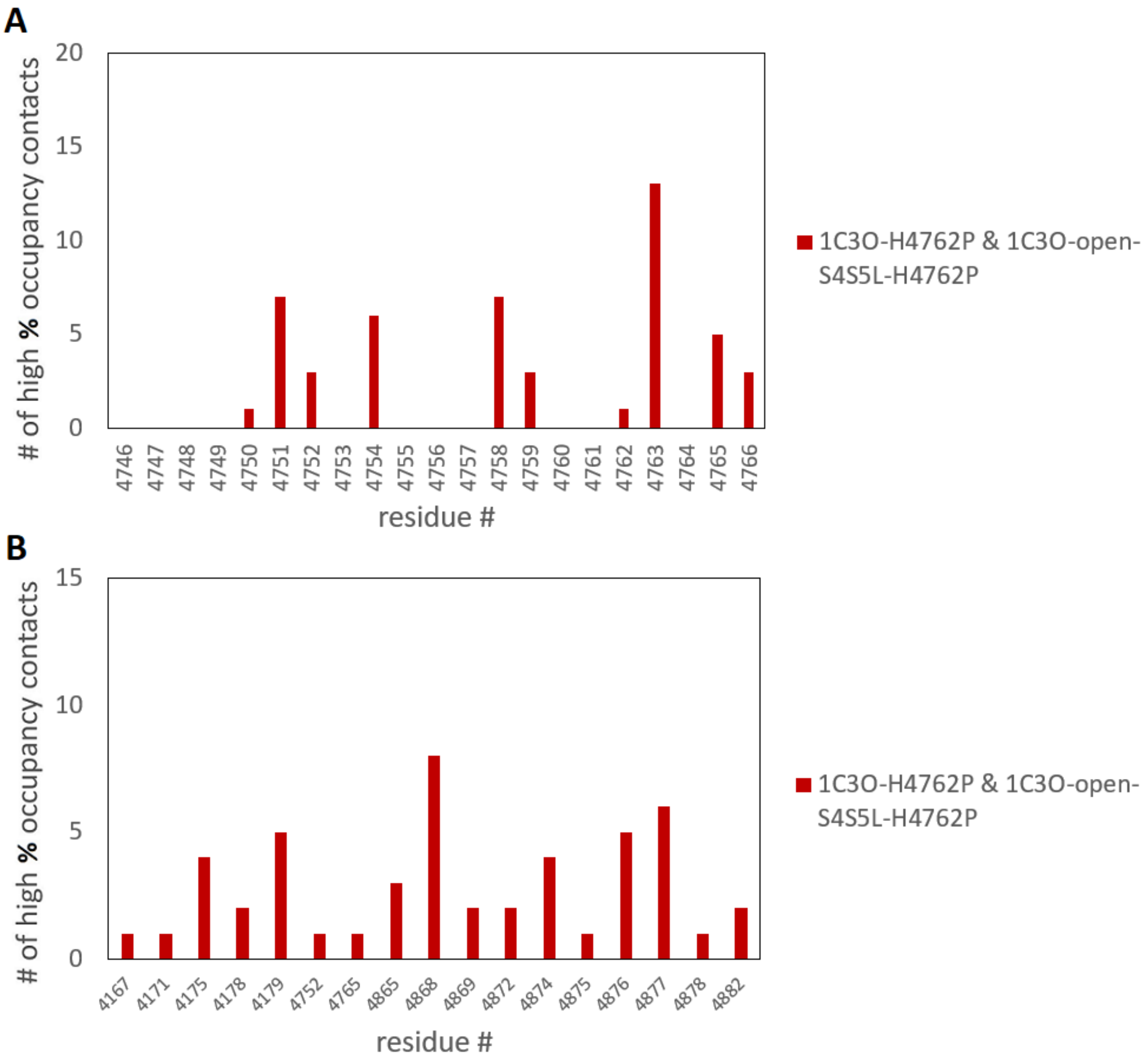

FIGURE S12. Number of S4S5L high percent occupancy interactions observed in the H4762P mutant RyR2 systems. The number of high percent occupancy interactions involving the S4S5L (residues 4746-4766) observed by residue number within the two mutant (1C3O-H4762P and 1C3O-open-S4S5L-H4762P) RyR2 systems is given above. The interactions at a given residue position within the S4S5L are given in (A) while the residues that the S4S5L was bound to are given in (B). In producing this figure, we pooled data from all four subunits for the 2 open mutant RyR2 systems (1C3O-H4762P and 1C3O-open-S4S5L-H4762P).

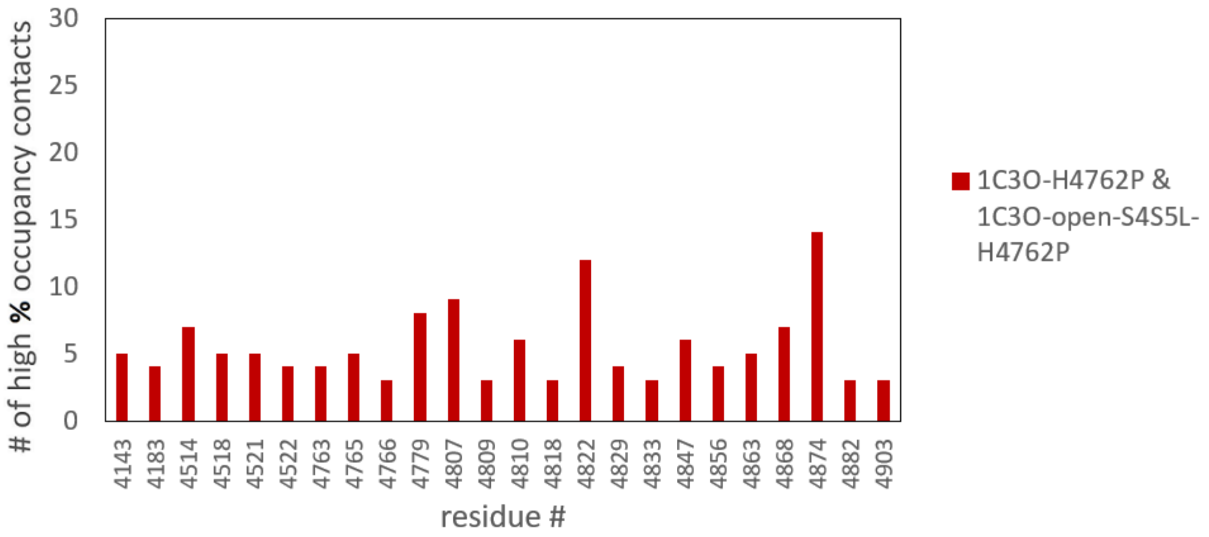

FIGURE S13. Number of inter-subunit high percent occupancy interactions observed in the H4762P mutant RyR2 systems. The number of high percent occupancy inter-subunit interactions observed by residue number within the two mutant (1C3O-H4762P and 1C3O-open-S4S5L-H4762P) RyR2 systems is given above. We pooled data from all four subunits for the 2 open mutant RyR2 systems (1C3O-H4762P and 1C3O-open-S4S5L-H4762P). To keep the x-axis at a reasonable length, we only included those interactions with at least 3 high percent occupancy contacts. The full high percent occupancy contact data set is available in **TABLES S3** and **S4**.

TABLES

4C			4O			1C3O			1C3O-open-S4S5L		
res. #1	res. #2	% occ.	res. #1	res. #2	% occ.	res. #1	res. #2	% occ.	res. #1	res. #2	% occ.
s1.4759	s1.4869	53	s1.4751	s1.4167	51	s1.4751	s1.4175	45	s1.4751	s1.4174	65
s1.4763	s1.4865	58	s1.4754	s1.4175	48	s1.4752	s4.4766	75	s1.4751	s1.4175	88
s1.4763	s1.4868	31	s1.4758	s1.4876	60	s1.4759	s1.4869	52	s1.4754	s1.4178	57
s2.4759	s2.4869	40	s1.4762	s1.4872	31	s1.4763	s1.4865	68	s1.4755	s1.4179	42
s2.4763	s2.4865	68	s1.4763	s2.4874	61	s1.4763	s1.4868	57	s1.4758	s1.4876	37
s2.4763	s2.4868	57	s1.4765	s2.4877	32	s2.4751	s2.4171	48	s1.4762	s2.4874	54
s3.4754	s3.4501	45	s2.4759	s2.4869	42	s2.4754	s2.4717	43	s1.4763	s1.4865	45
s3.4755	s3.4504	32	s2.4763	s2.4865	75	s3.4754	s3.4175	39	s1.4763	s1.4868	31
s3.4759	s3.4869	56	s2.4763	s2.4868	38	s3.4758	s3.4876	32	s1.4763	s2.4874	51
s3.4763	s3.4865	81	s2.4763	s3.4874	57	s3.4763	s3.4868	60	s2.4751	s2.4175	52
s3.4763	s3.4868	53	s2.4765	s3.4877	36	s3.4765	s4.4878	52	s2.4755	s2.4178	56
s4.4751	s4.4504	51	s2.4766	s3.4877	46	s3.4766	s4.4877	61	s2.4758	s2.4179	43
s4.4751	s4.4509	49	s3.4750	s3.4171	76	s4.4751	s4.4174	90	s2.4763	s3.4874	47
s4.4759	s4.4869	72	s3.4751	s3.4877	35	s4.4751	s4.4178	79	s2.4766	s3.4875	56
s4.4763	s4.4865	66	s3.4758	s3.4178	47	s4.4752	s4.4877	82	s2.4766	s3.4874	36
s4.4763	s4.4868	53	s3.4758	s3.4876	86	s4.4754	s4.4179	58	s3.4751	s3.4171	87
			s3.4763	s3.4865	63	s4.4759	s4.4872	70	s3.4752	s3.4877	81
			s3.4763	s3.4868	51	s4.4763	s4.4868	89	s3.4754	s3.4175	63
			s3.4763	s4.4874	31	s4.4766	s1.4752	75	s3.4754	s3.4179	44
			s3.4766	s4.4878	41				s3.4758	s3.4178	57
			s3.4766	s4.4751	73				s3.4763	s3.4868	36
			s4.4751	s3.4766	73				s3.4765	s4.4878	46
			s4.4751	s4.4878	57				s3.4766	s4.4874	52
			s4.4754	s4.4876	53				s4.4751	s4.4171	42
			s4.4758	s4.4872	55				s4.4754	s4.4179	38
			s4.4763	s4.4868	58				s4.4755	s4.4876	83
									s4.4758	s4.4872	53
									s4.4759	s4.4869	76
									s4.4762	s4.4872	41
									s4.4762	s1.4874	69

TABLE S1. High percent occupancy interactions involving the S4S5L. High percent occupancy interactions are provided for the 4C, 4O, 1C3O, and 1C3O-open-S4S5L systems. The S4S5L residue is indicated by “res. #1” while its binding partner is indicated by “res. #2”. The subunit that the residue belongs to is indicated using a prefix such that s3.4868 refers to residue 4868 on subunit 3, for instance. The percent occupancy over the last 100 frames (100 ns) of the 1 µs MD trajectory is given. High percent occupancy interactions are defined as those interactions which had a percent occupancy ≥ 30%. Interactions with a percent occupancy < 30% were omitted from the table.

1C3O-HID			1C3O-open-S4S5L-HID			1C3O-H4762P			1C3O-open-S4S5L-H4762P		
res. #1	res. #2	% occ.	res. #1	res. #2	% occ.	res. #1	res. #2	% occ.	res. #1	res. #2	% occ.
s1.4759	s1.4869	68	s1.4748	s1.4225	71	s1.4751	s1.4175	67	s1.4751	s1.4171	34
s1.4763	s1.4865	73	s1.4751	s1.4167	42	s1.4752	s4.4765	87	s1.4751	s1.4175	72
s1.4763	s1.4868	75	s1.4754	s1.4175	52	s1.4752	s1.4167	59	s1.4754	s1.4179	62
s2.4751	s2.4171	78	s1.4762	s2.4878	55	s1.4759	s1.4869	79	s1.4758	s1.4178	35
s2.4751	s2.4175	53	s1.4763	s1.4865	41	s2.4751	s2.4877	40	s1.4763	s1.4865	51
s2.4754	s2.4184	58	s1.4763	s2.4874	84	s2.4758	s2.4179	31	s1.4763	s1.4868	33
s2.4765	s3.4882	31	s1.4766	s2.4874	35	s2.4758	s2.4876	35	s1.4765	s2.4875	32
s3.4754	s3.4175	59	s2.4751	s2.4167	43	s2.4759	s2.4869	38	s1.4765	s2.4876	30
s3.4754	s3.4179	49	s2.4759	s2.4869	60	s2.4763	s2.4868	30	s2.4763	s2.4865	46
s3.4754	s3.4178	34	s2.4763	s2.4865	37	s2.4763	s3.4874	43	s2.4763	s2.4868	43
s3.4758	s3.4876	25	s2.4763	s2.4868	33	s2.4765	s3.4878	38	s2.4763	s3.4874	43
s3.4762	s3.4872	45	s2.4765	s3.4875	35	s2.4766	s3.4874	58	s3.4754	s3.4175	50
s3.4763	s3.4868	39	s2.4766	s3.4873	54	s3.4750	s3.4877	45	s3.4754	s3.4179	60
s3.4763	s4.4874	42	s2.4766	s3.4875	33	s3.4751	s3.4876	34	s3.4758	s3.4876	31
s4.4754	s4.4175	32	s3.4754	s3.4175	46	s3.4752	s3.4877	31	s3.4762	s4.4882	39
s4.4763	s4.4865	54	s3.4759	s3.4869	84	s3.4758	s3.4876	65	s3.4763	s3.4865	46
s4.4763	s4.4868	40	s4.4751	s4.4171	49	s3.4763	s3.4868	64	s3.4763	s3.4868	71
			s4.4754	s4.4171	82	s4.4751	s4.4175	46	s3.4765	s4.4882	65
			s4.4754	s4.4175	54	s4.4754	s4.4178	49	s3.4766	s4.4874	83
			s4.4759	s4.4869	46	s4.4754	s4.4179	41	s4.4751	s4.4877	66
						s4.4758	s4.4872	43	s4.4754	s4.4179	41
						s4.4759	s4.4868	75	s4.4758	s4.4872	60
						s4.4763	s4.4868	58	s4.4763	s4.4868	85
						s4.4765	s1.4752	87	s4.4763	s1.4877	37
						s4.4766	s1.4877	33			

TABLE S2. High percent occupancy interactions involving the S4S5L. High percent occupancy interactions are provided for the 1C3O-HID, 1C3O-open-S4S5L-HID, 1C3O-H4762P, and 1C3O-open-S4S5L-H4762P systems. The S4S5L residue is indicated by “res. #1” while its binding partner is indicated by “res. #2”. The subunit that the residue belongs to is indicated using a prefix such that s3.4868 refers to residue 4868 on subunit 3, for instance. The percent occupancy over the last 100 frames (100 ns) of the 1 μs MD trajectory is given. High percent occupancy interactions are defined as those interactions which had a percent occupancy ≥ 30%. Interactions with a percent occupancy < 30% were omitted from the table.

4C			4O			1C3O			1C3O-open-S4S5L		
**res. #1**	**res. #2**	**% occ.**	**res. #1**	**res. #2**	**% occ.**	**res. #1**	**res. #2**	**% occ.**	**res. #1**	**res. #2**	**% occ.**
s1.4183	s2.4885	51	s1.4763	s2.4874	61	s1.4779	s2.4548	63	s1.4141	s2.4113	31
s1.4183	s2.4906	56	s1.4765	s2.4877	32	s1.4787	s2.4736	39	s1.4141	s2.4116	30
s1.4623	s2.4162	39	s1.4779	s2.4514	72	s1.4807	s2.4556	60	s1.4183	s2.4906	47
s1.4779	s2.4514	86	s1.4807	s2.4522	45	s1.4809	s2.4554	43	s1.4762	s2.4874	54
s1.4807	s2.4522	59	s1.4809	s2.4520	42	s1.4810	s2.4552	42	s1.4763	s2.4874	51
s1.4807	s2.4521	40	s1.4810	s2.4518	31	s1.4825	s2.4822	38	s1.4779	s2.4514	66
s1.4809	s2.4520	53	s1.4829	s2.4822	74	s1.4847	s2.4818	54	s1.4787	s2.4558	44
s1.4833	s2.4822	66	s1.4847	s2.4818	99	s1.4847	s2.4822	72	s1.4807	s2.4522	44
s1.4847	s2.4818	63	s1.4847	s2.4822	96	s1.4860	s2.4863	70	s1.4807	s2.4521	30
s1.4868	s2.4874	51	s1.4856	s2.4863	79	s1.4868	s2.4874	99	s1.4810	s2.4518	68
s1.4872	s2.4874	47	s1.4868	s2.4874	43	s1.4872	s2.4874	65	s1.4835	s2.4799	42
s1.4875	s2.4874	42	s1.4514	s4.4779	72	s1.4875	s2.4874	68	s1.4847	s2.4822	64
s1.4950	s2.4906	30	s1.4520	s4.4809	39	s1.4178	s2.4878	50	s1.4514	s4.4779	72
s1.4950	s2.4907	37	s1.4522	s4.4807	67	s1.4179	s2.4881	40	s1.4518	s4.4810	38
s1.4514	s4.4779	79	s1.4735	s4.4787	51	s1.4183	s2.4899	30	s1.4520	s4.4809	31
s1.4818	s4.4833	38	s1.4818	s4.4847	84	s1.4514	s4.4779	86	s1.4521	s4.4807	34
s1.4822	s4.4847	84	s1.4822	s4.4847	99	s1.4518	s4.4810	39	s1.4522	s4.4807	77
s1.4822	s4.4826	78	s1.4822	s4.4825	78	s1.4520	s4.4809	53	s1.4804	s4.4840	30
s1.4863	s4.4860	52	s1.4874	s4.4868	72	s1.4752	s4.4766	75	s1.4863	s4.4856	60
s1.4863	s4.4863	52	s1.4874	s4.4872	60	s1.4818	s4.4847	88	s1.4874	s4.4762	69
s1.4874	s4.4872	47	s1.4906	s4.4183	59	s1.4863	s4.4856	32	s2.4183	s3.4903	87
s1.4874	s4.4875	52	s2.4779	s3.4514	34	s1.4874	s4.4872	94	s2.4185	s3.4899	82
s1.4874	s4.4868	68	s2.4810	s3.4518	51	s1.4874	s4.4875	53	s2.4763	s3.4874	47
s1.4880	s4.4183	40	s2.4809	s3.4520	48	s1.4881	s4.4875	73	s2.4766	s3.4874	36
s1.4881	s4.4879	69	s2.4807	s3.4521	44	s1.4899	s4.4955	40	s2.4766	s3.4875	56
s1.4882	s4.4183	71	s2.4786	s3.4557	37	s1.4162	s4.4624	47	s2.4779	s3.4514	92
s1.4885	s4.4183	71	s2.4787	s3.4558	50	s1.4162	s4.4625	61	s2.4809	s3.4520	56
s1.4903	s4.4950	38	s2.4856	s3.4863	73	s2.4623	s3.4157	47	s2.4838	s3.4805	55
s2.4183	s3.4885	41	s2.4763	s3.4874	57	s2.4623	s3.4162	53	s2.4840	s3.4805	57
s2.4185	s3.4899	97	s2.4766	s3.4877	46	s2.4625	s3.4162	45	s2.4847	s3.4822	69
s2.4779	s3.4514	88	s2.4765	s3.4877	36	s2.4808	s3.4521	48	s2.4856	s3.4863	41
s2.4807	s3.4522	45	s3.4183	s4.4906	41	s2.4779	s3.4737	40	s2.4868	s3.4874	53
s2.4809	s3.4520	74	s3.4619	s4.4166	40	s2.4801	s3.4800	72	s2.4949	s3.4900	67
s2.4829	s3.4822	53	s3.4763	s4.4874	31	s2.4800	s3.4800	38	s2.4950	s3.4959	65
s2.4834	s3.4803	45	s3.4766	s4.4751	73	s2.4832	s3.4801	57	s2.4952	s3.4959	57
s2.4847	s3.4818	54	s3.4766	s4.4878	41	s2.4833	s3.4818	34	s2.4956	s3.4960	39
s2.4860	s3.4863	49	s3.4779	s4.4514	83	s2.4824	s3.4823	58	s3.4186	s4.4903	31
s2.4863	s3.4863	70	s3.4787	s4.4735	45	s2.4868	s3.4874	61	s3.4623	s4.4497	83
s2.4868	s3.4874	71	s3.4847	s4.4822	97	s2.4872	s3.4874	46	s3.4624	s4.4166	44
s2.4872	s3.4874	63	s3.4847	s4.4818	92	s2.4960	s3.4899	30	s3.4765	s4.4878	46
s2.4875	s3.4874	61	s3.4856	s4.4863	66	s3.4182	s4.4899	30	s3.4766	s4.4874	52
s2.4895	s3.4896	30	s3.4868	s4.4874	41	s3.4618	s4.4166	74	s3.4779	s4.4514	76
s2.4955	s3.4900	61				s3.4618	s4.4161	36	s3.4810	s4.4518	30
s3.4182	s4.4903	30				s3.4619	s4.4166	66	s3.4833	s4.4801	53
s3.4779	s4.4570	89				s3.4619	s4.4170	54	s3.4847	s4.4822	55
s3.4787	s4.4735	58				s3.4765	s4.4878	52	s3.4856	s4.4863	70
s3.4807	s4.4578	51				s3.4766	s4.4877	61	s3.4868	s4.4874	43
s3.4809	s4.4576	55				s3.4779	s4.4737	44			
s3.4810	s4.4574	48				s3.4807	s4.4521	56			
s3.4826	s4.4822	83				s3.4808	s4.4521	56			
s3.4829	s4.4822	44				s3.4847	s4.4818	53			
s3.4836	s4.4803	43				s3.4847	s4.4822	52			
s3.4847	s4.4822	72				s3.4860	s4.4863	67			
s3.4868	s4.4874	62				s3.4868	s4.4874	97			
s3.4872	s4.4874	48				s3.4872	s4.4874	63			
s3.4875	s4.4874	48				s3.4875	s4.4874	43			
s3.4879	s4.4881	49									

TABLE S3. Inter-subunit high percent occupancy interactions (prior page). High percent occupancy interactions are provided for the 4C, 4O, 1C3O, and 1C3O-open-S4S5L systems. The residue of the subunit is indicated by "res. #1" while its binding partner on an adjacent subunit is indicated by "res. #2". The subunit that the residue belongs to is indicated using a prefix such that s3.4868 refers to residue 4868 on subunit 3, for instance. The percent occupancy over the last 100 frames (100 ns) of the 1 μs MD trajectory is given. High percent occupancy interactions are defined as those interactions which had a percent occupancy ≥ 30%. Interactions with a percent occupancy < 30% were omitted from the table.

1C3O-HID			1C3O-open-S4S5L-HID			1C3O-H4762P			1C3O-open-S4S5L-H4762P		
res. #1	res. #2	% occ.	res. #1	res. #2	% occ.	res. #1	res. #2	% occ.	res. #1	res. #2	% occ.
s1.4613	s2.4157	37	s1.4762	s2.4878	55	s1.4623	s2.4497	65	s1.4765	s2.4875	32
s1.4624	s2.4166	36	s1.4763	s2.4874	84	s1.4624	s2.4166	59	s1.4765	s2.4876	30
s1.4779	s2.4736	57	s1.4766	s2.4874	35	s1.4655	s2.4124	65	s1.4779	s2.4514	59
s1.4840	s2.4801	51	s1.4787	s2.4735	38	s1.4779	s2.4514	60	s1.4787	s2.4735	31
s1.4847	s2.4818	72	s1.4807	s2.4522	58	s1.4807	s2.4522	64	s1.4807	s2.4521	44
s1.4847	s2.4822	77	s1.4807	s2.4521	58	s1.4809	s2.4520	61	s1.4829	s2.4822	74
s1.4860	s2.4863	69	s1.4809	s2.4520	52	s1.4810	s2.4518	31	s1.4856	s2.4863	87
s1.4863	s2.4863	75	s1.4825	s2.4822	84	s1.4824	s2.4822	38	s1.4868	s2.4874	37
s1.4868	s2.4874	85	s1.4843	s2.4803	41	s1.4825	s2.4822	62	s1.4143	s2.4899	56
s1.4872	s2.4874	52	s1.4847	s2.4818	39	s1.4847	s2.4818	79	s1.4184	s2.4881	43
s1.4946	s2.4130	32	s1.4847	s2.4822	50	s1.4847	s2.4822	71	s1.4514	s4.4779	67
s1.4182	s2.4885	51	s1.4856	s2.4863	75	s1.4856	s2.4863	75	s1.4518	s4.4810	44
s1.4183	s2.4885	44	s1.4868	s2.4874	81	s1.4141	s2.4111	71	s1.4520	s4.4809	52
s1.4514	s4.4779	43	s1.4893	s2.4959	82	s1.4143	s2.4963	60	s1.4522	s4.4807	89
s1.4518	s4.4810	68	s1.4896	s2.4959	56	s1.4942	s2.4120	34	s1.4523	s4.4786	99
s1.4520	s4.4809	40	s1.4897	s2.4962	45	s1.4950	s2.4903	47	s1.4523	s4.4790	52
s1.4522	s4.4807	62	s1.4955	s2.4900	78	s1.4236	s4.4779	67	s1.4558	s4.4787	74
s1.4809	s4.4840	42	s1.4956	s2.4962	72	s1.4240	s4.4810	30	s1.4795	s4.4833	47
s1.4822	s4.4829	41	s1.4183	s2.4906	42	s1.4242	s4.4809	35	s1.4795	s4.4840	67
s1.4822	s4.4847	59	s1.4514	s4.4779	77	s1.4244	s4.4807	60	s1.4822	s4.4829	49
s1.4899	s4.4887	32	s1.4520	s4.4809	36	s1.4752	s4.4765	87	s1.4874	s4.4868	75
s1.4906	s4.4183	44	s1.4522	s4.4807	61	s1.4822	s4.4847	83	s1.4874	s4.4872	47
s2.4765	s3.4882	31	s1.4803	s4.4836	44	s1.4822	s4.4828	31	s1.4877	s4.4763	37
s2.4779	s3.4514	65	s1.4805	s4.4836	36	s1.4863	s4.4855	75	s1.4881	s4.4875	67
s2.4806	s3.4558	47	s1.4822	s4.4833	55	s1.4874	s4.4868	61	s2.4779	s3.4514	76
s2.4808	s3.4521	45	s1.4822	s4.4847	66	s1.4877	s4.4766	33	s2.4836	s3.4800	56
s2.4809	s3.4520	55	s1.4874	s4.4868	40	s1.4882	s4.4183	38	s2.4837	s3.4801	71
s2.4825	s3.4822	33	s1.4874	s4.4872	57	s1.4885	s4.4183	34	s2.4833	s3.4818	61
s2.4847	s3.4822	77	s1.4874	s4.4875	63	s1.4903	s4.4183	76	s2.4833	s3.4822	61
s2.4868	s3.4874	53	s1.4899	s4.4895	30	s1.4903	s4.4184	32	s2.4856	s3.4863	81
s2.4872	s3.4874	46	s1.4900	s4.4956	37	s1.4959	s4.4950	47	s2.4868	s3.4874	30
s2.4956	s3.4962	71	s2.4656	s3.4120	30	s1.4959	s4.4143	30	s2.4763	s3.4874	43
s3.4141	s4.4111	65	s2.4779	s3.4514	42	s1.4960	s4.4143	32	s3.4143	s4.4993	56
s3.4143	s4.4897	78	s2.4809	s3.4520	36	s2.4779	s3.4514	50	s3.4185	s4.4899	48
s3.4183	s4.4885	66	s2.4847	s3.4818	31	s2.4810	s3.4518	67	s3.4613	s4.4124	33
s3.4183	s4.4906	55	s2.4856	s3.4863	34	s2.4807	s3.4521	34	s3.4657	s4.4117	70
s3.4619	s4.4162	69	s2.4766	s3.4873	54	s2.4808	s3.4521	40	s3.4659	s4.4108	40
s3.4651	s4.4120	36	s2.4765	s3.4875	35	s2.4807	s3.4522	87	s3.4762	s4.4882	39
s3.4652	s4.4120	35	s2.4766	s3.4875	33	s2.4836	s3.4799	55	s3.4763	s4.4874	35
s3.4656	s4.4123	31	s3.4143	s4.4963	31	s2.4847	s3.4818	100	s3.4765	s4.4882	65
s3.4763	s4.4874	42	s3.4144	s4.4963	42	s2.4829	s3.4822	60	s3.4766	s4.4874	83
s3.4779	s4.4514	45	s3.4779	s4.4514	50	s2.4847	s3.4822	99	s3.4779	s4.4514	75
s3.4807	s4.4522	88	s3.4807	s4.4522	34	s2.4856	s3.4863	70	s3.4807	s4.4522	39
s3.4807	s4.4521	58	s3.4809	s4.4520	46	s2.4766	s3.4874	58	s3.4807	s4.4521	48
s3.4808	s4.4521	38	s3.4810	s4.4518	33	s2.4868	s3.4874	41	s3.4810	s4.4518	85
s3.4829	s4.4822	47	s3.4824	s4.4818	68	s2.4763	s3.4874	43	s3.4829	s4.4822	43
s3.4847	s4.4818	65	s3.4856	s4.4863	57	s2.4765	s3.4878	38	s3.4847	s4.4822	100
s3.4847	s4.4822	69	s3.4868	s4.4874	36	s2.4963	s3.4949	40	s3.4868	s4.4874	66
s3.4860	s4.4863	70	s3.4955	s4.4899	52	s3.4183	s4.4906	30	s3.4892	s4.4900	51
s3.4863	s4.4863	63	s3.4956	s4.4959	93	s3.4779	s4.4514	64			
s3.4868	s4.4874	49				s3.4805	s4.4562	37			
s3.4872	s4.4874	43				s3.4807	s4.4521	74			
s3.4936	s4.4111	34				s3.4810	s4.4518	75			
						s3.4838	s4.4800	40			
						s3.4838	s4.4801	73			
						s3.4868	s4.4874	64			
						s3.4872	s4.4874	42			
						s3.4954	s4.4900	53			

TABLE S4. Inter-subunit high percent occupancy interactions (prior page). High percent occupancy interactions are provided for the 1C3O-HID, 1C3O-open-S4S5L-HID, 1C3O-H4762P, and 1C3O-open-S4S5L-H4762P systems. The residue of the subunit is indicated by “res. #1” while its binding partner on an adjacent subunit is indicated by “res. #2”. The subunit that the residue belongs to is indicated using a prefix such that s3.4868 refers to residue 4868 on subunit 3, for instance. The percent occupancy over the last 100 frames (100 ns) of the 1 μs MD trajectory is given. High percent occupancy interactions are defined as those interactions which had a percent occupancy ≥ 30%. Interactions with a percent occupancy < 30% were omitted from the table.

4099-4206		
**Mutation**	**Closest inter-sub. high % occ. (>30%) contact**	**# of inter-sub high % occ. contacts**
*N4104I	4111	2
*N4104K	4111	2
*L4105F	4111	2
H4108N	4111	2
H4108Q	4111	2
S4124G	4123	1
R4144C	4143	1
E4146K	4143	1
*Y4149S	4143	1
R4157Q	4157	2
T4158P	4157	2
Q4159P	4157	2
N4178S	4178	1
E4178Q	4178	1
*T4196A	4185	1
*Q4201R	4185	1
**4485-4963**		
**Mutation**	**Closest inter-sub. high % occ. (>30 %) contact**	**# of inter-sub. high % occ. contacts**
*R4497C	4514	7
*R4499C	4514	7
*M4504I	4514	7
A4510T	4514	7
F4511L	4514	7
A4556T	4556	1
S4565R	4570	1
*A4607P	4613	1
E4611K	4613	1
*W4645R	4651	1
K4650E	4651	1
V4653F	4652	1
Ins EY-4657	4656	1
Ins-EY-4658	4656	1
*G4662S	4656	1
*G4671R	4656	1
N4736 Del	4736	2
H4762P	4763	1
V4771I	4766	2
R4790Q	4787	2
K4805R	4806	1
R4822H	4822	17
I4848V	4847	15
F4851C	4847	15
A4860G	4860	6
I4867M	4868	10
V4880A	4880	1
N4895D	4895	1
P4902L	4903	2
P4902S	4903	2
G4936R	4936	1
E4950K	4950	3
E4959Q	4960	1

TABLE S5. Comparison of known CPVT and LQTS mutations to inter-subunit high percent occupancy interactions from the closed state systems (prior page). A comparison between known CPVT and LQTS mutations and high percent occupancy interactions (> 30% occupancy) found in our three closed state RyR2 systems (4C, 1C3O, and 1C3O-HID). Mutations marked with an asterisk (*) are greater than 5 residues away from an identified inter-subunit high percent occupancy interaction. Mutation data was taken from a study by Medeiros-Domingo et al.[6]
